# Dual‐Ionic Weakly Solvating Electrolyte Design Enables Efficient Fast‐Cycling of High‐Voltage Anion Shuttle Batteries

**DOI:** 10.1002/advs.202505982

**Published:** 2025-07-11

**Authors:** Jieun Kang, Inhui Lee, Gwonho Yu, Jin Jun Heo, Yuri Choi, Sangyeop Lee, Sungho Kim, Dongjoo Kim, Jungki Ryu, Seoin Back, Soojin Park, Jaegeon Ryu

**Affiliations:** ^1^ Department of Chemistry and Department of Battery Engineering Pohang University of Science and Technology (POSTECH) Pohang 37673 Republic of Korea; ^2^ Department of Chemical and Biomolecular Engineering Sogang University Seoul 04107 Republic of Korea; ^3^ Institute of Energy and Environment Sogang University Seoul 04107 Republic of Korea; ^4^ KU‐KIST Graduate School of Converging Science and Technology Korea University Seoul 02841 Republic of Korea; ^5^ School of Energy and Chemical Engineering Ulsan National Institute of Science and Technology (UNIST) Ulsan 44919 Republic of Korea; ^6^ Department of Integrative Energy Engineering Korea University Seoul 02841 Republic of Korea

**Keywords:** colloid, dual‐ion batteries, electrolyte, nano graphene oxide, weakly solvating electrolyte

## Abstract

Electrolytes shape solvation structures that govern ionic transport, stability, and interfacial properties in energy storage systems. Sodium‐based dual‐ion shuttling systems offer high‐voltage and fast‐charging potential but face challenges such as solvent co‐intercalation, electrolyte decomposition, and low Coulombic efficiency, partly due to limited anion‐focused electrolyte design. Herein, a low‐concentration dual‐ionic weakly solvating electrolyte (DWSE) is introduced, leveraging functionalized nano‐graphene oxide additives to modulate the solvation environments of Na^+^ and PF_6_^−^. While a conventional cationic weakly solvating electrolyte (CWSE) enhances Na^+^ transport, DWSE simultaneously addresses anion and cation transport for a more balanced approach. DWSE prevents solvent co‐intercalation, stabilizes interfaces with NaF‐rich layers, and enhances ionic transport. It achieves a reversible capacity of 82.0 mAh g^−1^ at 50 C and retains 96.2% capacity after 1500 cycles at 10 C. This study offers a robust framework for advancing dual‐ion shuttling systems with optimized cation and anion dynamics.

## Graphical abstract

A dual‐ionic weakly solvating electrolyte is developed to simultaneously regulate cation and anion transport in sodium‐based anion shuttle batteries. Functionalized nano‐graphene oxide additives reshape solvation environments, enabling high‐rate performance and long‐term stability. The study reveals a balanced electrolyte design that prevents solvent co‐intercalation and forms robust interphases, advancing dual‐ion energy storage systems.

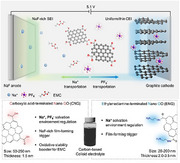

## 1 Introduction

Electrolytes are indispensable in advancing electrochemical energy storage systems, as they govern the formation of solvation structures through intricate molecular‐level interactions between cations, anions, and solvent molecules.^[^ ^1^ ^]^ These solvation structures exert a profound influence on ionic transport, electrochemical stability, and interfacial dynamics. Consequently, the rational design of solvation environments tailored to the unique requirements of specific systems and their charge carriers is paramount. In conventional rocking‐chair batteries, exemplified by lithium‐ion batteries (LIBs), where cations act as the primary charge carriers, significant advancements have been achieved in understanding and optimizing cation solvation.^[^ ^2^ ^]^ However, the critical role of counterions, particularly anions, has received comparatively limited attention. Recent investigations into anion chemistry have demonstrated that leveraging anions effectively can unlock new possibilities for enhancing electrochemical performance, enabling high‐voltage operation, and ultrafast charging.^[^ ^3^, ^4^, ^5^ ^]^

Anion shuttle batteries (or dual‐ion batteries (DIBs)), which operate via simultaneous anion intercalation into graphite cathodes and cation insertion or plating at the anode, have emerged as promising candidates for next‐generation energy storage.^[^ ^6^, ^7^ ^]^ These systems store energy by shuttling anions between electrodes, rather than relying solely on cation migration. Among these, sodium (Na)‐based anion shuttle batteries are especially attractive due to the natural abundance, low cost, and favorable redox potential of Na. These batteries demonstrate high power density (e.g., 42.9 kW kg^−1^ based on graphite mass), ultrafast charge capability (up to 100 C), and high‐voltage operation exceeding 5 V.^[^ ^8^, ^9^, ^10^ ^]^ However, despite these promising attributes, critical challenges hinder their practical implementation.^[^ ^11^ ^]^

On the cathode side, anion intercalation into graphite occurs at voltages exceeding the oxidative stability window of conventional carbonate‐based electrolytes. This results in irreversible electrolyte decomposition and the formation of resistive cathode electrolyte interphases (CEIs), which impede anion transport and reduce capacity retention. Furthermore, solvent co‐intercalation is often observed at low current densities, especially when anions are strongly solvated. This leads to severe structural degradation of the graphite through layer exfoliation and pore formation.^[^ ^12^ ^]^ Simultaneously, the Na metal anode, though offering high theoretical capacity, is highly reactive with conventional carbonate electrolytes, leading to the formation of unstable and fragile solid electrolyte interphases (SEIs).^[^ ^13^ ^]^ These SEIs are prone to mechanical failure and dendrite formation during repeated cycling, resulting in low Coulombic efficiency (CE), limited cycle life, and potential safety hazards such as short‐circuiting and thermal runaway.

To address these issues, many efforts have focused on developing highly concentrated electrolytes (HCEs), where the increased salt concentration facilitates the inclusion of anions in the primary solvation shell.^[^ ^14^, ^15^, ^16^, ^17^, ^18^ ^]^ This promotes interphase formation and suppresses solvent activity, thereby improving the oxidative stability of the electrolyte. However, HCEs suffer from limitations such as high viscosity, low ionic conductivity, poor wettability, and elevated cost.^[^ ^19^, ^20^ ^]^ To overcome these shortcomings, localized high‐concentration electrolytes (LHCEs) have been proposed, which dilute HCEs with inert cosolvents (e.g., hydrofluoroethers) to lower viscosity while preserving favorable solvation structures.^[^ ^21^ ^]^ Yet, LHCEs still face challenges such as high cost, limited electrochemical windows, and reliance on excessive anion populations to maintain interfacial stability.^[^ ^22^ ^]^ Additionally, the strong electrostatic interactions present in contact ion pairs (CIPs) and aggregated ion clusters (AGGs) found in HCEs and LHCEs impose high desolvation energy penalties.^[^ ^23^ ^]^ This impairs the ability of anions to effectively intercalate into graphite, and may even result in the intercalation of solvated complexes—leading to irreversible damage.

Conversely, weakly solvating electrolytes (WSEs) have been successfully applied in cation‐dominant systems like LIBs.^[^ ^24^, ^25^, ^26^, ^27^, ^28^ ^]^ By reducing the interaction between cations and solvents, WSEs lower desolvation barriers and stabilize electrode interfaces. However, their poor salt solubility and limited oxidative stability render them unsuitable for high‐voltage anion shuttle batteries. Therefore, a novel electrolyte design is needed that simultaneously addresses the stability of both electrodes and enables efficient dual‐ion (Na^+^ and PF_6_^−^) transport under low‐concentration salt conditions.

**Figure 1:**
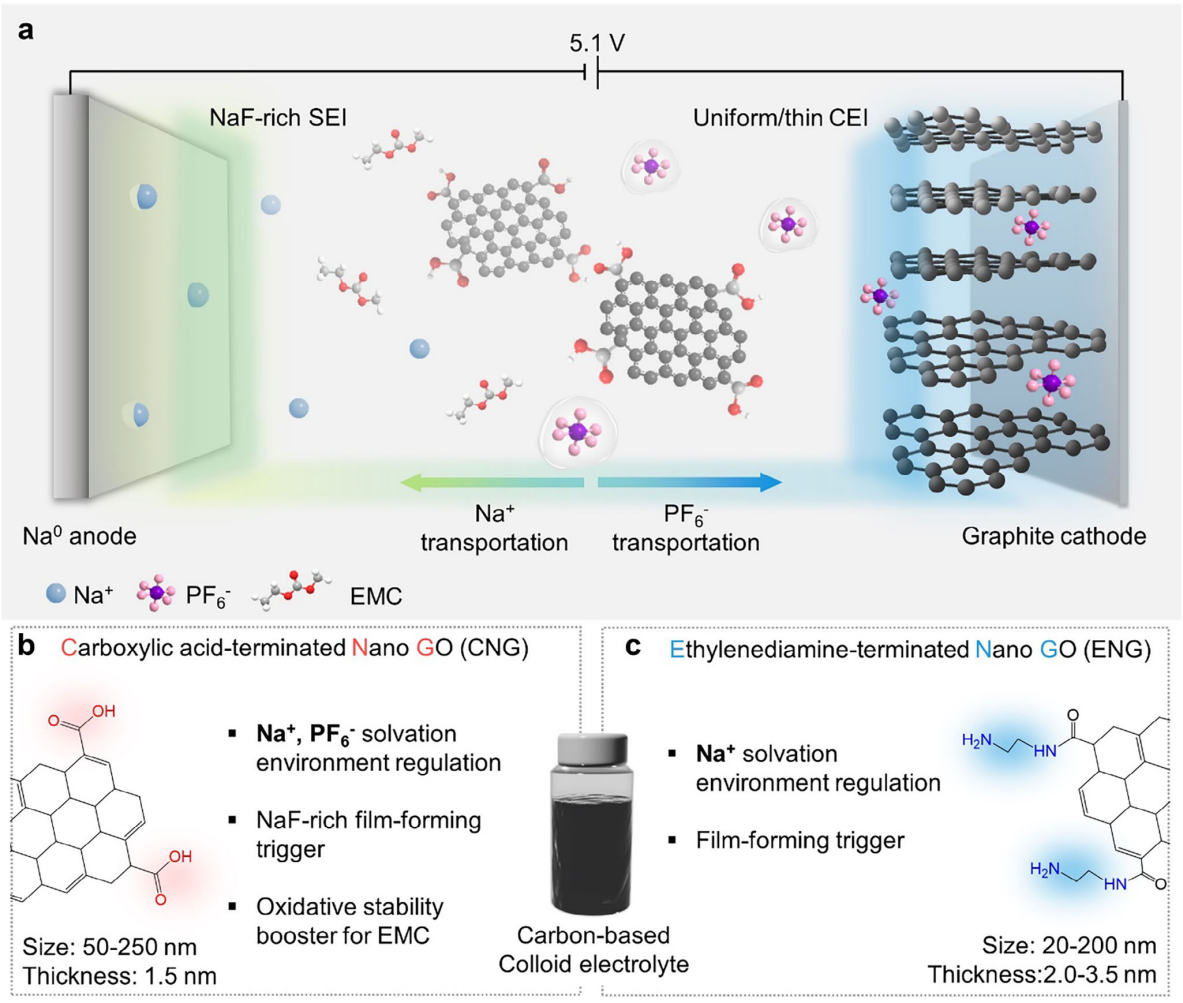
Electrolyte design principles. a) Schematic of the electrolyte design strategy of dual‐ionic weakly solvating electrolytes using carbonaceous colloids for anion shuttle batteries. b) Carboxylic acid‐functionalized NGO (CNG) for dual‐ionic weakly solvating electrolytes (DWSE) and c) ethylenediamine‐functionalized NGO (ENG) for cationic weakly solvating electrolytes (CWSE).

Herein, we present a dual‐ionic weakly solvating electrolyte (DWSE) strategy that simultaneously modulates the solvation environments of both cations and anions in the electrolyte (**Figure** **1**a). By incorporating colloidal electrolytes prepared with nano‐graphene oxides (NGOs) functionalized with distinct surface functionalities, we disrupt the conventional solvation structures of Na^+^ and PF_6_^−^ ions in low concentration electrolytes (LCEs). The effects of these additives on solvation environments, interface evolution at electrodes, and the resultant electrochemical performance of dual‐ion storage systems were comprehensively investigated. Ethylenediamine‐passivated NGOs (ENGs) strongly interact with Na^+^, weakening the solvation power of ethyl methyl carbonate (EMC) and forming a cationic weakly solvating electrolyte (CWSE) that enhances Na^+^ transport (Figure 1c). In contrast, carboxylic acid‐functionalized NGOs (CNGs) engage effectively with Na^+^ and PF_6_^−^, establishing a DWSE that optimizes the transport dynamics of dual ions (Figure 1b). Additionally, CNGs act as film‐forming triggers by promoting the interfacial decomposition of fluoroethylene carbonate (FEC), forming NaF‐rich interphases, which significantly enhance the cycling stability and CE of Na metal anodes and graphite cathodes. Consequently, dual‐ion Na metal batteries incorporating DWSE achieved 82.0 mAh g^−1^ at 50 C (1 C = 100 mA g^−1^) and retained 96.2% capacity after 1500 cycles at 10 C. This study maintains low salt concentrations while effectively optimizing the solvation environments of oppositely charged ions. More importantly, it offers valuable insights into electrolyte design for systems utilizing both cations and anions as charge carriers.

## 2 Results and Discussion

### 2.1 Preparation of DWSE and CWSE Electrolytes by Carbon‐Based Additives

NGO colloids (i.e., GO nanocolloids) have garnered significant attention in biomedical fields such as drug delivery due to their exceptional properties, including a large surface area, high chemical and mechanical stability, and tunable electrical and optical characteristics.^[^ ^29^, ^30^ ^]^ The oxygenated surface groups of NGOs allow for further chemical modification or functionalization, greatly expanding their structural and chemical versatility. While the sp^3^‐based insulating characteristics of NGOs limit their applicability as active materials in batteries, their potential as electrolyte additives was explored in this study. We hypothesized that the tunable surface functional groups of NGOs could significantly alter the solvation environment in the electrolyte, thereby improving the electrochemical performance of the system.

CNG and ENG were selected for their distinct chemical properties, which were expected to influence their interactions with the electrolyte components (EMC, FEC, Na^+^, and PF_6_^−^). Carboxylic acid groups on CNG are well‐known for their ability to form strong hydrogen bonds and interact with a wide range of molecules, including polar species. These characteristics suggest that CNG would interact significantly with both Na^+^ and PF_6_^−^, potentially modifying the solvation structures of both ions. In contrast, the secondary amine groups in ENG, with their strong coordination ability, were anticipated to primarily alter the Na^+^ solvation structure while having a weaker influence on PF_6_^−^. Given the importance of enhancing the mobility of both Na^+^ and PF_6_^−^ in anion shuttle batteries, we aimed to investigate how the different functional groups in CNG and ENG influence the solvation environment and ion transport. Thereby, we sought to identify the role of tailored surface functionalization in optimizing electrolyte performance for dual‐ion storage applications.

Functionalized NGOs were synthesized through a two‐step oxidation of carbon nanofibers, yielding CNGs.^[^ ^31^, ^32^ ^]^ Subsequent modification with ethylenediamine produced ENGs (see Methods for details). The successful synthesis and functionalization of these NGOs were verified through comprehensive material characterizations (Figures S1–S5, Supporting Information). DWSE and CWSE were prepared by dispersing CNG and ENG, respectively, into a base electrolyte (BE) comprising 1.0 m NaPF_6_ in EMC with 5 wt% FEC, followed by vigorous magnetic stirring to achieve colloidal stability (Figure S6, Supporting Information). The BE was chosen for its optimal balance of low concentration, high ionic conductivity, cost‐effectiveness, and compatibility with commercially relevant electrolyte systems. Top‐view and cross‐sectional scanning electron microscopy (SEM) images confirmed that the porous structure of the separator remained unchanged after soaking in CWSE and DWSE containing NGO additives (Figure S7, Supporting Information).

### 2.2 Solvation Environments in DWSE and CWSE

**Figure 2:**
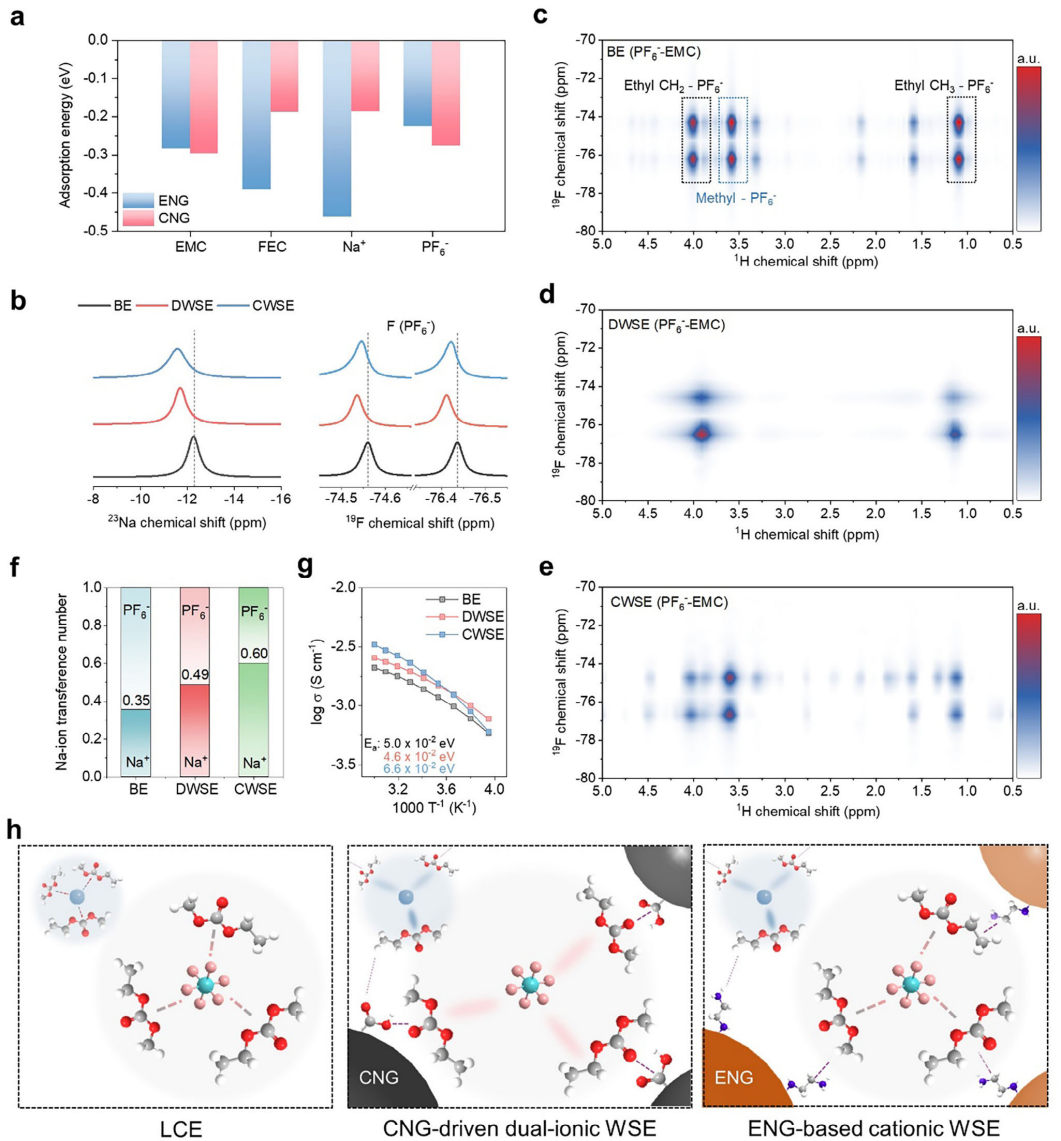
Na^+^ and PF_6_^−^ solvation environment. a) Adsorption energies for electrolyte components on CNG and ENG. b) ^23^Na and ^19^F NMR spectra of BE, DWSE, and CWSE. 2D ^1^H‐^19^F HOESY spectra of c) BE, d) DWSE, and e) CWSE. f) Na^+^ transference number of BE, DWSE, and CWSE. g) Arrhenius plots for the ionic conductivities at various temperatures ranging from −20 to 60 °C. h) Schematic of the solvation environments of BE, DWSE, and CWSE.

To investigate the interactions between NGOs and the electrolyte components, we performed density functional theory (DFT) calculations to determine adsorption energies (**Figure** **2**a; Figure S8, Supporting Information). For ENG, the adsorption energy for Na^+^ (−0.462 eV) was stronger than that for PF_6_^−^ (−0.226 eV), suggesting that ENG predominantly interacts with Na^+^, likely due to the strong coordination capability of the ethylenediamine functional groups. These interactions are expected to destabilize the Na^+^ solvation sheath by attracting both Na^+^ and EMC. In contrast, CNG exhibited the strongest adsorption for EMC (−0.296 eV), followed by PF_6_^−^ (−0.276 eV), demonstrating its effectiveness in disrupting the solvation structure of PF_6_^−^. Notably, CNG exhibited more uniform adsorption behavior compared to ENG. The difference in adsorption energies between Na^+^ and PF_6_^−^ was 0.089 eV, significantly smaller than that observed for ENG (0.236 eV). This suggests that CNG is capable of modulating the solvation structures of both cationic and anionic species, potentially balancing their transport properties within the electrolyte. Additionally, we note that adsorption energies are stronger on the functionalized NGOs (ENG and CNG) compared to the bare NGO, emphasizing the importance of functional group design in tailoring electrolyte interactions (Figure S9, Supporting Information). Zeta potential measurements further confirmed the preferential interactions of the NGO additives (Figure S10, Supporting Information). Upon dispersing CNG and ENG in EMC and BE, CNG exhibited a more negative surface potential, consistent with PF_6_^−^ adsorption, while ENG showed a positive shift, indicating Na^+^ adsorption. These findings highlight the preferential interactions of PF_6_^−^ with the carboxylic acid groups of CNG and Na^+^ with the amine groups of ENG, further validating their role in modulating solvation structures within the electrolytes.

To investigate the changes in the solvation structures of Na^+^ and PF_6_^−^ in DWSE and CWSE, 1D and 2D nuclear magnetic resonance (NMR) analyses were conducted. In the LCE, Na^+^ and PF_6_^−^ are fully solvated by EMC and are dissociated. The ^23^Na chemical shift showed a downfield shift in the order of CWSE (−11.57 ppm) > DWSE (−11.70 ppm) > BE (−12.29 ppm), indicating significantly weakened Na^+^‐EMC interactions in CWSE (Figure 2b). This observation is consistent with the computational results, which showed a strong adsorption energy of Na^+^ on ENG. In DWSE, Na^+^‐EMC interactions are also weakened, but to a lesser extent than CWSE. The ^19^F chemical shift of PF_6_^−^ exhibited a downfield shift in the order of DWSE (−74.54 ppm, −76.41 ppm) > CWSE (−74.55 ppm, −76.42 ppm) > BE (−74.56 ppm, −76.44 ppm), suggesting that PF_6_^−^‐EMC interactions are most weakened in DWSE. Thus, CWSE predominantly weakens the solvation structure of Na^+^, while DWSE disrupts the solvation structures of Na^+^ and PF_6_^−^, with a particularly strong effect on PF_6_^−^. Raman spectroscopy revealed only subtle peak shifts in DWSE, supporting that the solvation structure is moderately weakened but does not promote the formation of contact ion pairs or aggregated ions (Figure S11, Supporting Information).

The change in the solvation environment of PF_6_^−^ is further explained using heteronuclear Overhauser effect spectroscopy (HOESY), which is a powerful tool for probing spatial binding strength. 2D ^1^H‐^19^F HOESY clearly revealed the interactions between PF_6_^−^ and the hydrogen atoms on the methyl and ethyl groups of EMC. In the BE, PF_6_^−^ showed uniform cross‐peaks with the methyl and ethyl hydrogens of EMC, indicating that a uniform solvation structure was formed (Figure 2c). Interestingly, in the DWSE, the interactions between PF_6_^−^ and the methyl hydrogens of EMC completely disappeared, while those with the ethyl hydrogens became rather weak, suggesting that the anion solvation structure was reconfigured (Figure 2d). This can be attributed to the preferential adsorption configuration of the methyl group in EMC on CNG, which causes PF_6_^−^ to primarily interact with the ethyl group on the opposite side (Figure S12, Supporting Information). In contrast, CWSE retained the cross‐peaks observed in BE, but with reduced intensity, particularly for interactions between PF_6_^−^ and the methyl group of EMC (Figure 2e). This is likely due to ENG interacting with both the methyl and ethyl groups of EMC, with a relatively stronger binding to the ethyl group. In summary, CWSE selectively weakens the solvation structure of Na^+^, whereas DWSE disrupts the solvation structures of Na^+^ and PF_6_^−^, with a particularly pronounced effect on PF_6_^−^. These results provide key insights into the role of functionalized NGOs in modulating the solvation environments of dual‐ion electrolytes, highlighting their potential for tailoring ionic interactions to optimize electrochemical performance.

In anion shuttle systems, achieving a balance between cation and anion mobility is critical for optimizing performance.^[^ ^33^ ^]^ The Bruce‐Vincent method was employed to measure the Na^+^ transference number as a quantitative indicator of ion transport. In CWSE, the selective weakening of the cation solvation structure resulted in a significant increase in the Na^+^ transference number, from 0.35 in BE to 0.60 (Figure 2f). This reduction in Na^+^–solvent interactions facilitates faster Na^+^ transport. In contrast, DWSE exhibited a comparable weakening of Na^+^ and PF_6_^−^ solvation structures, resulting in a Na^+^ transference number of 0.49. This value aligns closely with the ideal balance required for anion shuttle systems^[^ ^34^ ^]^ where similar transport properties of both ions minimize concentration polarization and enhance overall ionic conductivity (Figure 2f; Figure S13, Supporting Information). These results highlight the role of solvation structure modulation by functionalized NGO additives. While CWSE promotes rapid Na^+^ transport by selectively destabilizing its solvation sheath, DWSE achieves a balanced transport of Na^+^ and PF_6_^−^, facilitating ionic equilibrium and enhancing electrochemical performance in anion shuttle systems. Despite the dispersed nature of the electrolytes, both CWSE and DWSE maintained ionic conductivity comparable to that of the conventional LCE. Notably, DWSE exhibited the lowest activation energy for ion transport among the electrolytes, indicating its ability to effectively facilitate ion migration by minimizing resistance through the modulation of solvation structures (Figure 2g; Figure S14, Supporting Information). A schematic of solvation structure modifications in LCE, CWSE, and DWSE is shown in Figure 2h. CWSE primarily weakens the cation solvation structure, significantly enhancing cation mobility. In contrast, DWSE achieves a balanced weakening of cation and anion solvation structures, simultaneously optimizing ionic conductivity and reducing migration resistance.

### 2.3 Electrochemical Performance of Na||Graphite Cells

To determine the optimal concentration, various DWSE formulations were tested by adjusting the concentration of CNG (Figure S15, Supporting Information). Even at a low concentration of 0.1 mg mL^−1^, CNG significantly improved rate performance; however, it provided limited enhancement in long‐term cycling stability. Conversely, a high concentration of CNG (50 mg mL^−1^) led to excessive decomposition of CNG (discussed later) and disrupted ion transport pathways, resulting in poor rate performance. A concentration of 10 mg mL^−1^ was identified as optimal for CNG, and this concentration was subsequently used for ENG in the preparation of CWSE.

**Figure 3:**
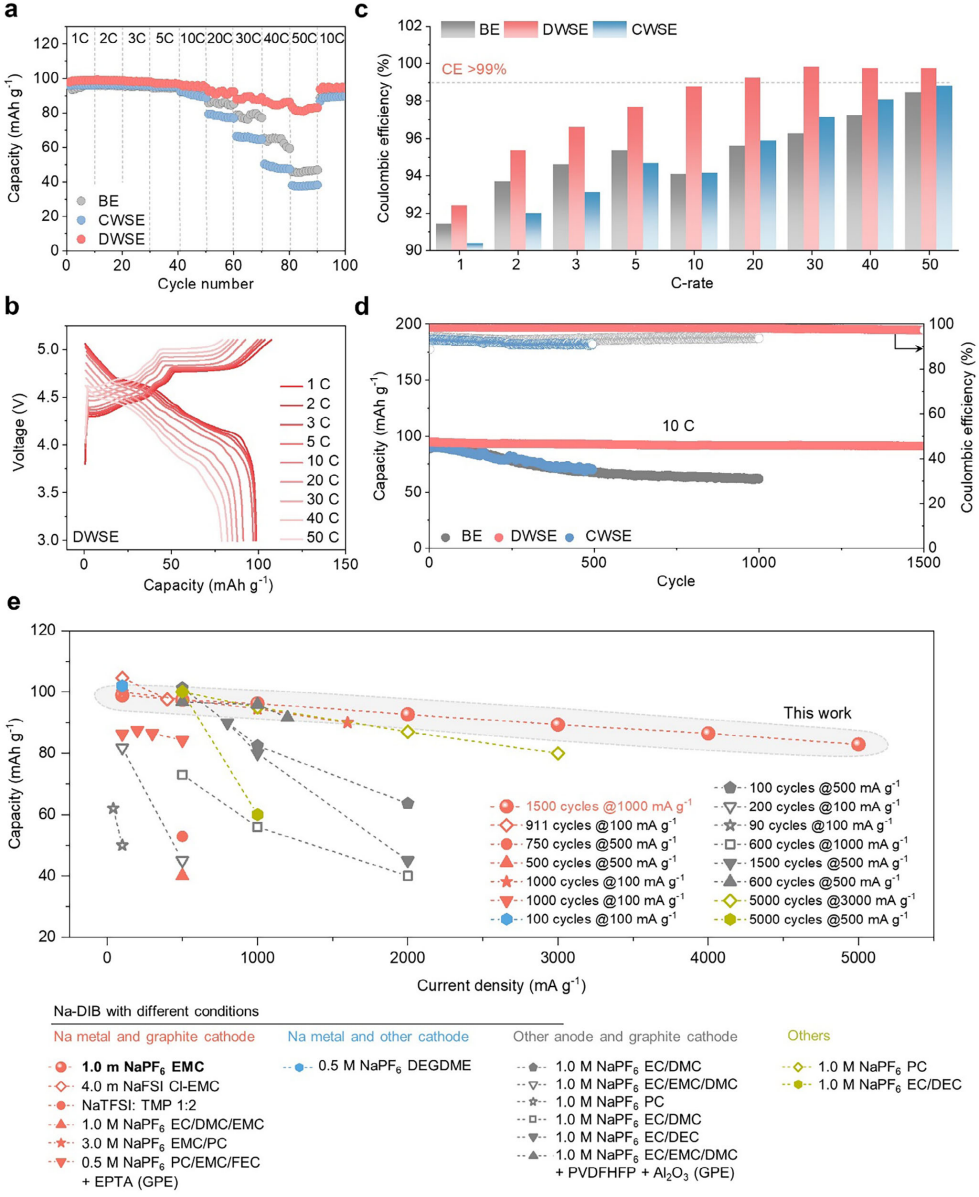
Electrochemical performance of Na||Graphite batteries in various electrolytes. a) Rate performance at 3.0–5.1 V from 1 C to 50 C. b) Galvanostatic voltage profiles of DWSE‐based cell at different C‐rates. c) CE comparison for the three electrolytes. d) Long‐term cycling stability of DIB cells using the three electrolytes at 10 C. e) Performance plot with previous studies (Graph annotations indicate cell lifespan at specific current density; corresponding references are listed in Table S1, Supporting Information).

DWSE demonstrated superior performance under ultrafast charging conditions of up to 50 C in DIBs (**Figure** **3**a,b; Figure S16, Supporting Information). DWSE‐based DIB exhibited discharge capacities of 95.5, 91.8, 88.3, 85.6, and 82.0 mAh g^−1^ at 10, 20, 30, 40, and 50 C, respectively. In contrast, CWSE showed lower performance, attributed to ENG obstructing PF_6_^−^ transport pathways rather than enhancing its mobility. DWSE significantly improved CE (Figure 3c). CE is typically low in Na metal‐based DIBs due to high‐voltage electrolyte decomposition, interfacial instability at the Na metal anode, and solvent co‐intercalation into the graphite cathode. The oxidative decomposition of BE was confirmed through linear sweep voltammetry and leakage current tests (Figure S17, Supporting Information). DWSE mitigates these issues, resulting in a substantial enhancement in CE. Voltage profiles at 1 and 10 C highlight the unique advantages of DWSE (Figure S18, Supporting Information). The observed decrease in charge capacity and increase in discharge capacity suggest near‐theoretical anion intercalation into graphite with reduced oxidative decomposition of the electrolyte at high voltages. This also indicates that anions are reversibly deintercalated during discharge, contributing to improved cycling stability. In contrast, CWSE and BE show reduced discharge capacity, likely due to oxidative electrolyte decomposition and solvent co‐intercalation.^[^ ^23^ ^]^ Overall, DWSE demonstrates its ability to optimize anion mobility and minimize side reactions, highlighting its potential for high‐rate DIB systems. The initial three CV cycles reveal a slight shift in the anion intercalation peak during the first cycle when NGO is present (Figure S19, Supporting Information). From the second cycle onward, DWSE stabilizes rapidly, showing the most pronounced and sharp redox peaks among the electrolytes.

In long‐term cycling stability tests, the DWSE‐based cell operated stably for over 1500 cycles at 10 C, highlighting its extended cycling performance (Figure 3d). In contrast, the CWSE‐based cell exhibited similar performance to BE, indicating limited enhancements. Accumulated Coulombic inefficiency during cycling was markedly lower in DWSE compared to BE (Figure S20, Supporting Information). Differences in coin cell swelling after 500 cycles further suggest that DWSE effectively mitigates gas generation, a common issue in BE (Figure S21, Supporting Information). Electrochemical impedance spectroscopy (EIS) analysis revealed that DWSE rapidly stabilized resistance within 10 cycles, underscoring its ability to maintain low internal resistance over extended cycling (Figure S22, Supporting Information). These findings demonstrate that DWSE is a highly effective strategy for DIBs, where balanced cation and anion transport is critical. In contrast, CWSE, which has shown advantages in LIBs, appears unsuitable for DIBs due to its inability to facilitate efficient anion transport. Additionally, DWSE maintained stable cycling and excellent rate performance even under high mass loading conditions (15 mg cm^−2^), further validating its practicality for application‐relevant DIB configurations (Figure S23, Supporting Information).

The performance of DWSE was thoroughly compared with previously reported Na‐based anion shuttle batteries (Figure 3e). Na‐based anion shuttle batteries can be categorized into four groups based on the types of anodes and cathodes used: i) Na metal anode and graphite cathode,^[^ ^11^, ^16^, ^18^, ^35^, ^36^ ^]^ ii) Na metal anode with non‐graphite cathodes,^[^ ^37^, ^38^, ^39^, ^40^, ^41^, ^42^ ^]^ iii) non‐Na metal anodes with graphite cathodes,^[^ ^43^ ^]^ and iv) other combinations.^[^ ^44^, ^45^ ^]^ Most studies on Na‐based anion shuttle batteries have focused on improving the anode while using graphite as the cathode. For anodes other than Na metal, conventional electrolytes typically consist of 1.0 m NaPF_6_ in carbonate solvents. In contrast, Na metal anodes, which face compatibility issues with carbonate electrolytes, often employ high‐concentration electrolytes based on imide salts such as NaFSI or NaTFSI. Anion shuttle batteries with Na metal anodes (pink plots) exhibit particularly low performance under high current densities, likely due to interfacial instability, Na dendrite formation, and slow ion transport in the electrolyte. DWSE achieves high reversible capacities and long‐term cycling stability (>1500 cycles) even under high current densities. These improvements are attributed to key advancements in both the Na metal anode and the graphite cathode, which will be discussed in detail. Performance metrics are provided in Table S1 (Supporting Information) for further comparison.

Additionally, the DWSE‐based cell demonstrated excellent cycling stability, maintaining over 400 stable cycles at 60 °C (Figure S24, Supporting Information). This performance is attributed to the formation of a stable electrode‐electrolyte interface and the mitigation of degradation pathways that are typically accelerated under high voltage and high‐temperature conditions. For instance, the reduction in free EMC population near the electrode interface is likely a contributing factor to the improved stability.

### 2.4 NGO as a Trigger for SEI Formation on Na Metal Anodes

**Figure 4:**
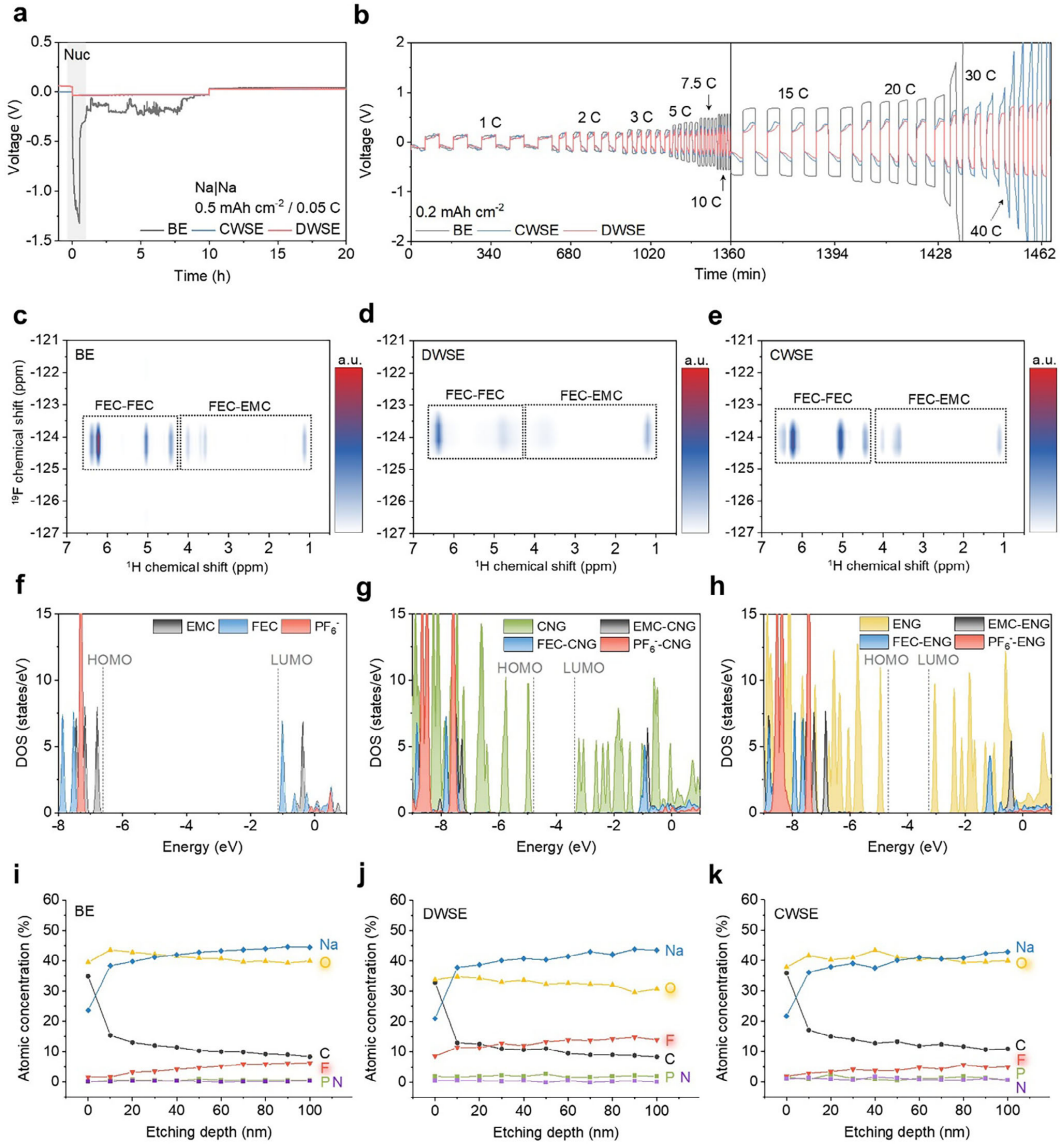
SEI chemistry for the Na metal anode. a) Voltage profiles of Na|Na symmetric cells during the first cycle at 0.05 C at 0.5 mAh cm^−2^. b) Galvanostatic cycling performance of Na|Na symmetric cells at 0.2 mAh cm^−2^ from 1 to 40 C. 2D ^1^H‐^19^F HOESY spectra showing the FEC region for c) BE, d) DWSE, and e) CWSE. The projected DOS of electrolyte components for f) BE, g) DWSE, and h) CWSE. For interacting electrolyte components with CNG or ENG, only the DOS of the electrolyte components was plotted. Depth profiles of atomic concentrations in Na metal anodes after 30 cycles, obtained through XPS analysis for i) BE, j) DWSE, and k) CWSE.

NaPF_6_‐carbonate solvent‐based LCE systems exhibit poor compatibility with Na metal due to interfacial instability, Na dendrite formation, and insufficient ionic conductivity at the anode interface. To evaluate the potential of the designed electrolytes for Na metal anodes, Na|Na symmetric cells were assembled without graphite cathodes for performance comparison. BE exhibited a significantly larger Na nucleation overpotential, reflecting its higher energy barrier for forming Na^0^ nuclei (**Figure** **4**a). Testing across C‐rates from 1 to 40 C at a capacity of 0.2 mAh cm^−2^ showed consistently high overpotentials for BE. DWSE exhibited the lowest overpotentials, confirming its suitability for Na metal anode applications (Figure 4b). The interfacial stability of the electrolytes was further evaluated through symmetric Na|Na cell cycling.^[^ ^46^, ^47^ ^]^ As shown in Figure S25a (Supporting Information), at a lower areal capacity of 0.2 mAh cm^−2^ and 1 C, the DWSE‐based cell exhibited the lowest and most stable overpotential over 200 h, highlighting its ability to suppress dendritic growth and maintain a stable SEI even under soft cycling conditions. In addition, long‐term cycling at a higher capacity of 0.5 mAh cm^−2^ and 1 C (Figure S25b, Supporting Information) further confirmed the superior interfacial compatibility of DWSE, with the cell remaining stable for over 400 h, whereas BE and CWSE cells failed within 150 and 300 h, respectively. These results underscore the role of DWSE in forming a robust and uniform NaF‐rich SEI that sustains Na plating/stripping over extended durations.

Temperature‐dependent EIS measurements, combined with the Arrhenius equation, revealed that DWSE achieved the lowest R_SEI_ and R_ct_ values among the electrolytes (Figure S26, Supporting Information). Charge transfer performance evaluated through Tafel plots also demonstrated that DWSE and CWSE outperformed BE (Figure S27, Supporting Information). Post‐cycling analysis of Na metal anodes after 500 cycles in DIBs revealed non‐uniform surface morphology and the presence of inactive Na (dead Na) in BE. DWSE and CWSE exhibited more homogeneous and stable surfaces (Figure S28, Supporting Information). These effects are strongly associated with the SEI chemistry on the Na metal anode.

2D ^1^H‐^19^F HOESY analysis showed that in BE, the 5 wt% FEC interacted primarily with other FEC molecules and EMC in the electrolyte (Figure 4c). A similar interaction was observed in CWSE (Figure 4e), but DWSE exhibited weakened FEC‐FEC and FEC‐EMC interactions (Figure 4d). CNG interacts with FEC, modifying its solvation environment and creating more free FEC molecules (Figure 2a). Specifically, the finely tuned interactions between CNG and electrolyte components are hypothesized to promote a more favorable distribution of FEC around the Na metal anode. To investigate the energy levels of electrolyte components, we performed the density of states (DOS) calculations. The energy levels of the BE components (Figure 4f), CNG and its interacting electrolyte components (Figure 4g), as well as ENG and its corresponding interacting components (Figure 4h), were calculated. Among the electrolyte constituents, CNG and ENG exhibited low LUMO levels, suggesting that they could be preferentially reduced at the Na metal anode surface during the initial cycles. Their reduced forms facilitate electron transfer to nearby electrolyte components, primarily FEC, which has a low LUMO level, thereby initiating the SEI formation process. Cyclic voltammetry measurements of the initial three cycles of the electrolyte showed high current peaks of DWSE and CWSE during the first cycle, corresponding to the SEI (and CEI) formation triggered by NGOs. As cycling progressed, the SEI stabilized, leading to a significant decrease in peak current (Figure S29, Supporting Information). This observation implies that once the SEI and CEI layers are formed in the early stages of cycling, they act as protective interphases that suppress further electrochemical decomposition of the NGO additives. As a result, voltage‐induced degradation of NGOs is effectively prevented, which is consistent with the high CE maintained throughout extended cycling. In DWSE, the higher probability of free FEC being present at the Na metal anode surface, combined with continuous Na‐ion flux during charging, facilitates the efficient formation of NaF. The formation of NaF‐rich SEI on the Na metal anode is particularly advantageous due to its high ionic conductivity and superior chemical and thermal stability. NaF effectively mitigates dendrite growth and interfacial side reactions, which are major challenges in Na metal anodes. Ar^+^ sputter‐assisted X‐ray photoelectron spectroscopy (XPS) depth profiling revealed that, despite being an LCE, DWSE formed a NaF‐rich SEI layer with a structure and composition rivaling those achieved by HCEs and LHCEs (Figure 3i–k; Figure S30, Supporting Information). This underscores the exceptional ability of DWSE to stabilize Na metal anodes under challenging high‐rate and high‐temperature conditions.

### 2.5 CEI Chemistry for Graphite Cathodes

**Figure 5:**
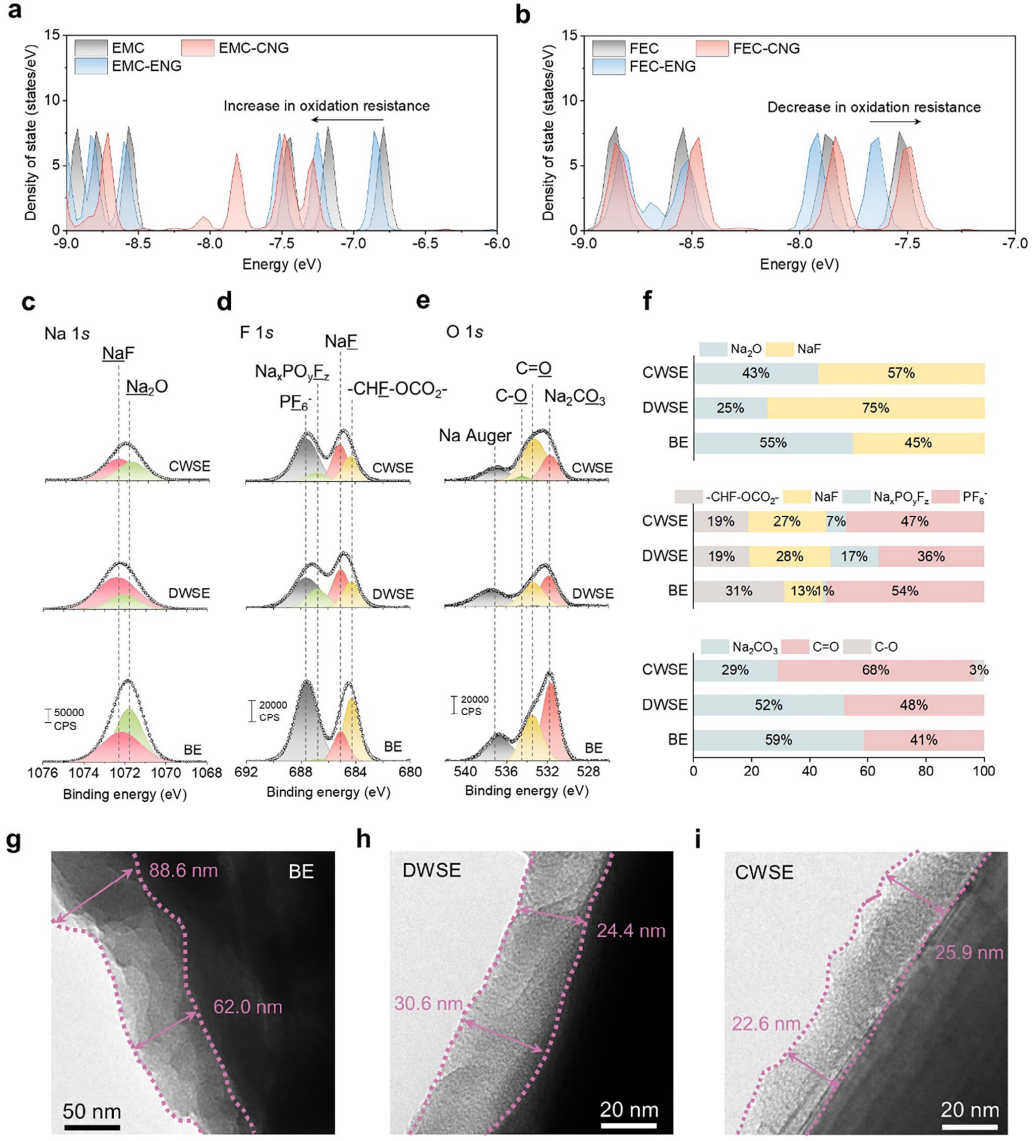
CEI chemistry for graphite cathodes. The DOS near the HOMO levels of a) EMC and b) FEC, showing changes when interacting with CNG and ENG. XPS analysis of graphite cathodes after 30 cycles in BE, DWSE, and CWSE‐based cells: c) Na 1*s*, d) F 1*s*, and e) O 1*s* spectra. f) Calculated proportions of CEI components based on XPS data. TEM images of graphite cathodes from cells using g) BE, h) DWSE, and i) CWSE.

CEI chemistry on the graphite cathode acting as an anion host was analyzed after cycling. The DOS calculations of NGOs in Figure 4g,h indicate that CNG and ENG are probable to be preferentially oxidized at the graphite cathode surface due to their low oxidative stability, as suggested by their high HOMO levels. This initial oxidation triggers further reactions with other electrolyte components, facilitating the formation of the CEI layer. Additionally, the DOS calculations reveal that the interaction between EMC and CNG lowers the HOMO level of EMC, thereby increasing its oxidative stability (**Figure** **5**a). This stabilization suppresses decomposition reactions under high‐voltage conditions, which is critical for maintaining performance in anion shuttle systems. Similarly, the interaction between FEC and CNG raises the HOMO level of FEC, reducing its oxidative stability (Figure 5b). This interaction promotes the preferential oxidation of FEC, resulting in the formation of NaF as a key component of the CEI, which enhances the chemical stability of the graphite cathode under high‐voltage conditions. These findings suggest that DWSE incorporating CNG enhances high‐voltage stability and facilitates the formation of a robust FEC‐driven CEI.

XPS analysis was conducted to investigate the CEI components in the different electrolytes. BE showed stronger peaks in Na 1*s*, F 1*s*, and O 1*s* spectra compared to DWSE and CWSE, indicating thicker CEI layers and excessive side reactions in BE. As shown in the Na 1*s* (Figure 5c) and F *1s* (Figure 5d) spectra, the CEI layer formed in DWSE contained higher NaF content and fewer trapped PF_6_^−^ ions compared to BE. The relative composition of CEI components for each electrolyte is presented in Figure 5f. BE exhibited a high Na_2_O content due to solvent decomposition, along with an increased formation of ─CHF‐OCO_2_─ and a lower proportion of NaF. Significant accumulation of unintercalated PF_6_^−^ ions was also observed in the CEI layer of BE. In contrast, DWSE formed a CEI with relatively higher NaF content, fewer organic species, and reduced PF_6_^−^ trapping. The O 1*s* spectra further revealed substantial solvent‐derived byproducts in BE, such as C─O/C═O bonds and Na_2_CO_3_ (Figure 5e).

High‐resolution transmission electron microscopy (HR TEM) analysis revealed significant differences in the CEI layers depending on the electrolyte. BE formed a thick and non‐uniform CEI layer ranging from 62.0 to 88.6 nm (Figure 5g). This was attributed to continuous oxidative decomposition of the electrolyte and structural changes in graphite at high voltages, leading to complex chain reactions. In contrast, DWSE (Figure 5h) and CWSE (Figure 5i) formed thinner and more uniform CEI layers. This improvement is attributed to the prompt formation of a stable SEI by NGOs, which effectively suppresses further side reactions during cycling. Therefore, DWSE effectively facilitates the formation of a thin and stable NaF‐rich CEI layer, which minimizes side reactions and enhances the performance of graphite cathodes under high‐voltage conditions.

### 2.6 Structural Evolution of Graphite Cathodes

Designing electrolytes that prevent the co‐intercalation of anions and solvents into graphite in the form of anionic complexes is critical for enhancing the performance and lifespan of anion shuttle batteries. When anions are strongly solvated by solvents, they encounter high energy barriers during desolvation, resulting in incomplete desolvation and intercalation into graphite as anionic complexes. These large complexes reduce the capacity of anion shuttle batteries by limiting the number of anions that can be accommodated in a single‐stacking mode within the graphene layers and cause structural degradation of the graphite.^[^ ^23^ ^]^ To address these challenges, it is essential to create a favorable environment for anion desolvation and to monitor the anion intercalation process through real‐time analysis.

**Figure 6:**
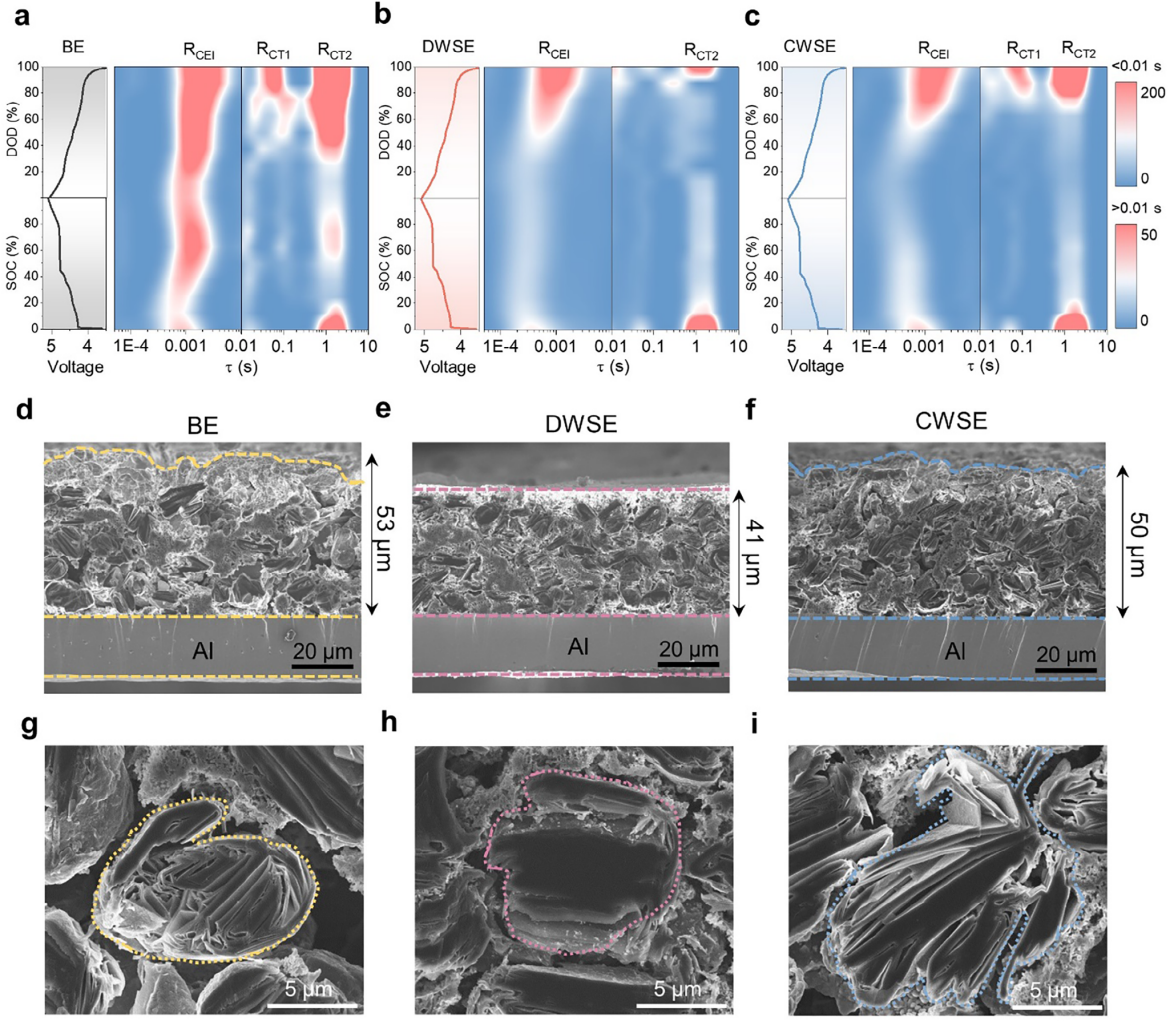
Structural evolution of graphite cathodes. Contour plots of DRT analysis for the first cycle of DIB cells using a) BE, b) DWSE, and c) CWSE. Cross‐sectional SEM images of graphite cathodes after 500 cycles for d) BE, e) DWSE, and f) CWSE‐based cells, with g–i) magnified views of the corresponding cathodes.

In operando EIS (Figure S31, Supporting Information) combined with distribution of relaxation times (DRT) analysis, was employed to investigate the resistances associated with anion CEI migration and anion (de‐)intercalation during the first charge‐discharge cycle. This analysis serves as a crucial tool for understanding the real‐time mechanisms of anion intercalation and deintercalation. Peaks in the 0.0001–0.01 s range represent the CEI resistance (R_CEI_), while peaks in the 0.01–10 s range correspond to the charge transfer process resistance (R_CT_). DIB cells using BE exhibited significantly high R_CEI_ and two distinct R_CT_ peaks (R_CT1_ and R_CT2_) during the first charge–discharge cycle (**Figure** **6**a). R_CT1_ is interpreted as the resistance associated with the intercalation of anionic complexes, while R_CT2_ reflects the resistance of free anion intercalation. This interpretation stems from the tendency of anionic complexes, which strongly interact with solvents, to intercalate into graphite without desolvation due to their lower energy barrier. In contrast, free anions require desolvation, which involves higher energy barriers and thus occurs over a longer timescale.

DWSE (Figure 6b) and CWSE (Figure 6c) exhibited lower R_CEI_ values during the first charge‐discharge cycle due to the formation of thin and uniform CEI layers. However, differences were observed in the R_CT_ range between DWSE and CWSE. DWSE showed only R_CT2_, indicating that it effectively reduced the desolvation energy of anions, enabling the formation of free anions and ensuring that only free anions were intercalated into graphite. On the other hand, CWSE exhibited both R_CT1_ and R_CT2_, suggesting that it could not entirely prevent the co‐intercalation of solvents and anions into graphite. Overall, BE formed a thick and unstable CEI layer, resulting in high R_CEI_ values and failing to suppress solvent co‐intercalation. CWSE formed a stable CEI but did not sufficiently modulate the solvation structure of anions, allowing solvent co‐intercalation to persist. In contrast, DWSE formed a stable and uniform CEI while effectively modifying the solvation structure of anions, enabling the intercalation of only free anions and preventing solvent co‐intercalation. These properties significantly improved the performance and stability of anion shuttle battery cells.

Graphite cathodes exhibit a distinctive staging mechanism during anion intercalation, balancing between the ionic forces of intercalated anions and the van der Waals forces between graphite layers. This process is characterized by distinct shifts and splits in the graphite (002) peak, reflecting changes in interlayer spacing. To investigate the structural changes in graphite cathodes under different electrolytes, in operando synchrotron X‐ray diffraction (XRD) was employed to monitor real‐time structural evolution during charge‐discharge cycles (Figure S32, Supporting Information). To clearly observe the differences in electrolyte behavior, a harsh experimental condition was applied by increasing the graphite cathode mass loading to 4 mg cm^−2^ and operating the cell at 5 C. DWSE‐based DIB cell demonstrated a reversible split and shift of the graphite (002) peak from 26.5° to 24° and 32.5° during charging, returning to its original position during discharging. This behavior indicates reversible anion (de‐)intercalation. The large peak at 29.5° corresponds to the Na metal anode, which was penetrated by the synchrotron beam. In contrast, BE exhibited a reduced intensity of the graphite (002) peak at 26.5° with incomplete shifts and a limited shift to 23° during charging. This is attributed to strongly solvated anions failing to achieve sufficient kinetics under harsh conditions and co‐intercalating with solvents into graphite. Under these conditions, free anions intercalated into graphite partially induced (002) peak shifts, while anionic complexes were limited in their intercalation, causing the (002) peak to remain at 26.5°. CWSE exhibited a similar trend to BE.

To evaluate the structural stability of graphite cathodes during cycling, cross‐sectional SEM images were obtained for graphite cathodes subjected to 500 cycles of anion (de‐)intercalation in BE, DWSE, and CWSE. The graphite cathode with BE (Figure 6d,g) exhibited a 220% vertical expansion compared to pristine graphite (Figure S33, Supporting Information), becoming uneven and porous with collapsed spherical graphite particles. In contrast, DWSE (Figure 6e,h) maintained a dense and intact structure, while CWSE (Figure 6f,i) showed structural collapse and exfoliation similar to BE. Ex situ Raman spectroscopy confirmed the effectiveness of the designed electrolytes in preserving the integrity of graphite particles (Figure S34, Supporting Information). After 500 cycles, the I_D_/I_G_ ratios of graphite cathodes in BE, DWSE, and CWSE were 0.59, 0.32, and 0.45, respectively. The lower ratio in DWSE indicates sufficient channels for anion (de‐)intercalation, enabling greater anion storage and preserving graphite integrity. Time‐of‐flight secondary ion mass spectrometry (TOF‐SIMS) analysis further revealed that DWSE exhibited the lowest levels of electrolyte‐derived byproducts, demonstrating its superior ability to maintain structural stability during cycling (Figure S35, Supporting Information). In conclusion, the analysis of structural changes in graphite cathodes highlights the critical role of weakening anion solvation sheaths in enhancing anion mobility and preserving the structural integrity of graphite cathodes. These findings emphasize the necessity of developing weak solvation electrolytes for anion shuttle systems.

## 3 Conclusion

This study developed DWSE and CWSE electrolytes incorporating carbon‐based additives with distinct functional groups: CNG (─COOH groups) and ENG (─NH_2_ groups), to address key challenges in Na‐based anion shuttle batteries. Detailed analysis using 1D and 2D NMR and DFT calculation revealed that DWSE effectively modulated the solvation environment by facilitating interactions between CNG, Na^+^, PF_6_^−^, and EMC. This created a weak solvation structure favorable for anion desolvation and transport, achieving a Na^+^ transference number of 0.49 and balanced ion mobility. In contrast, ENG‐based CWSE primarily influenced cation solvation, resulting in suboptimal performance with persistent anion desolvation and solvent co‐intercalation issues. Electrochemical testing demonstrated that DWSE delivered a reversible capacity of 82.0 mAh g^−1^ at 50 C and maintained stable cycling for over 1500 cycles at 10 C—one of the highest performances reported for Na‐based anion shuttle batteries. The prevention of solvent co‐intercalation in DWSE was due to both the formation of an effective CEI and the provision of a weak solvation environment for anions, enabling reversible (de‐)intercalation and minimizing parasitic reactions. By forming NaF‐rich SEI and CEI layers on Na metal and graphite electrodes, DWSE suppressed structural degradation and improved long‐term stability. These findings highlight the potential of weak solvation electrolytes, like DWSE, for advancing high‐performance, long‐life Na‐based anion shuttle batteries and energy storage technologies.

## 4 Experimental Section

### Materials

Sodium metal and natural spherical graphite were sourced from DAEJUNG and BTR, respectively. Sodium hexafluorophosphate (NaPF_6_), ethyl methyl carbonate (EMC), and fluoroethylene carbonate (FEC) were purchased from Welcos. Carbon nanofibers (20–200 µm) were obtained from Sigma–Aldrich.

### Synthesis of NGOs and Preparation of the Electrolytes

Carbon nanofibers (20–200 µm) were oxidized through a two‐step process.^[^ ^31^, ^32^ ^]^ In the first step, 10 mL of concentrated H_2_SO_4_ was added to a mixture of carbon nanofibers (0.4 g), P_2_O_5_ (0.3 g), and K_2_S_2_O_8_ (0.3 g). The mixture was heated to 80 °C and stirred for 4.5 h. And then, 100 mL of deionized water was slowly added to the mixture. The resulting mixture was filtered using a PVDF membrane (0.45 µm pore size) and dried overnight at 60 °C. In the second step, 50 mL of concentrated H_2_SO_4_ was added to the obtained black powder. KMnO_4_ (2 g) was gradually added in portions to keep the reaction temperature below 20 °C. The mixture was warmed to 35 °C and stirred for 24 h. Afterward, 50 mL of deionized water and 5 mL of 30% H_2_O_2_ were slowly added using a water bath. Following this, 100 mL of 3.4% HCl was added to the mixture, followed by dialysis (SpectraPore MWCO 10 kD) for 2 days to remove the salts and unreacted chemicals. The resulting mixture was freeze‐dried to obtain CNG powder.

CNG was modified with amine functional groups for the ENG. First, 200 mg of CNG was dispersed in deionized water (1 mg mL^−1^). N‐(3‐Dimethylaminopropyl)‐N’‐ethylcarbodiimide hydrochloride (1.2 g) and 10 mL of ethylenediamine were added to the suspension and stirred for 24 h at room temperature. Following the reaction, the suspension was dialyzed (SpectraPore MWCO 10 kD) for 2 days to remove the salts and unreacted chemicals and freeze‐dried to obtain ENG powder.

The synthesized NGO were added to a solution of 1.0 m NaPF_6_ in EMC with 5 wt% FEC under an argon atmosphere at target concentrations of 0.01–50 mg mL^−1^. The mixture was stirred vigorously for 7 days to ensure uniform distribution of the NGO additive.

### Physical Characterization

The physical properties of the synthesized nano graphene oxides (NGOs) were analyzed using various techniques, including atomic force microscopy (AFM, Dimension ICON, Bruker Nano Surface), transmission electron microscopy (TEM, JEM‐2100F, JEOL), X‐ray photoelectron spectroscopy (XPS, K‐alpha, ThermoFisher), Fourier‐transform infrared spectroscopy (FT‐IR, 670/620, Varian), X‐ray diffraction (XRD, SmartLab, RIGAKU), Raman spectroscopy (alpha300R, WITec), and Brunauer–Emmett–Teller (BET) surface area analysis (ASAP2020, Micromeritics Instruments). To investigate bulk electrolyte properties, 1D ^23^Na and ^19^F nuclear magnetic resonance spectroscopy (1D NMR, AVANCE 3 HD, Bruker), 2D ^1^H‐^19^F heteronuclear Overhauser effect spectroscopy (2D HOESY NMR, AVANCE NEO 600, Bruker), and zeta potential measurements (Nano ZS, Malvern) were conducted. All NMR measurements were performed using a coaxial tube setup, with CD_3_CN‐d_3_ in the outer tube serving as the lock solvent. All NGO and electrolyte characterizations were carried out at the UNIST Central Research Facilities (UCRF). Morphological analysis of graphite electrodes was primarily performed using field‐emission scanning electron microscopy (FE‐SEM, S‐4800, Hitachi) operated at 5 kV and high‐resolution field‐emission transmission electron microscopy (HR FE‐TEM, JEM‐2200FS with Image Cs‐corrector, JEOL) operated at 200 kV. Changes in graphite structure were confirmed using Raman spectroscopy (alpha300R, Oxford Instruments). FE‐SEM, HR FE‐TEM, and Raman spectroscopy were conducted at the National Institute for Nanomaterials Technology (NINT). The chemical composition of the cathode electrolyte interphase (CEI) and solid electrolyte interphase (SEI) was determined by X‐ray photoelectron spectroscopy (XPS, K‐alpha, ThermoFisher). The composition of electrodes after cycling was further supported by time‐of‐flight secondary ion mass spectrometry (TOF‐SIMS, TOF‐SIMS 5, ION TOF). In operando X‐ray diffraction (XRD) analysis of graphite electrodes was performed at the Pohang Accelerator Laboratory (PAL) 9A beamline.

### Electrochemical Measurement

Electrochemical properties of the electrolytes, including cyclic voltammetry (CV), ionic conductivity, and transference numbers, were measured using a potentiostat (SP‐300, BioLogic). CV was conducted at a scan rate of 1 mV s^−1^. Ionic conductivity was determined through electrochemical impedance spectroscopy (EIS) over a frequency range of 1 MHz–100 mHz. Transference numbers were calculated by performing EIS measurements before and after chronoamperometry at 0.01 V, over the same frequency range.

For sodium‐graphite full cells, the cathode was prepared using spherical graphite, polyacrylic acid, and Super‐P in an 80:10:10 weight ratio. The slurry was cast onto aluminum foil and dried in a vacuum oven at 120 °C for 8 h. The cathodes were punched into disk shapes with a loading level of 1.5–2.0 mg cm^−2^. The sodium‐graphite full cells were assembled using a sodium metal anode, a polyvinylidene fluoride separator, and a liquid electrolyte. Galvanostatic cycling tests for sodium‐graphite DIBs were performed at a voltage range of 3.0–5.1 V versus Na/Na^+^ using a battery cycler (WBCS 3000Le32, Wonatech). High‐temperature cycling tests were conducted at 60 °C and 20 C under the same voltage range. EIS measurements were carried out between 1 MHz and 100 mHz using a potentiostat (VMP3, BioLogic). All electrodes used in the electrochemical tests were assembled into 2032‐type coin cells inside an argon‐filled glovebox. Operando X‐ray diffraction (XRD) experiments were conducted at a current density of 5 C, within the voltage range of 3.0–5.1 V. The distribution of relaxation times (DRT) analysis was conducted using DRT Tools, developed by the Ciucci Group (GitHub: ciuccislab/DRTtools), within the MATLAB environment. The transformation from frequency to time domain was based on the following general formula:

(1) $$Z\left(\omega \right)=R_{\infty }+\int_{0}^{\infty }\frac{\gamma \left(\tau \right)}{1+j\omega \tau }d\tau$$

where Z(ω) represents the impedance as a function of frequency, R_∞_ is the ohmic impedance of the battery, τ is the specific relaxation time, and γ(τ) is the distribution function of relaxation times.

### Computational Methods

Spin‐polarized density functional theory (DFT) calculations were performed using the Vienna Ab Initio Package (VASP) code version 5.4.4.^[^ ^48^, ^49^ ^]^ The projector augmented wave (PAW) pseudopotential method and the Perdew–Burke–Ernzehof (PBE)^[^ ^50^ ^]^ exchange‐correlation functional were used.^[^ ^51^, ^52^ ^]^ The van der Waals interactions were described using Grimme's D3 method.^[^ ^53^ ^]^ The kinetic energy cutoff was set to 400 eV, and (1 × 1 × 1) gamma‐point centered k‐point grids were sampled for all structures.^[^ ^54^ ^]^ The convergence tolerance of energy and force was set to 10^−4 ^eV and 0.05 eVÅ^−1^, respectively. The solvent environment was modeled as a polarizable continuum using the linearized Poisson–Boltzmann implicit solvation method, as implemented in VASPsol.^[^ ^55^, ^56^ ^]^ A dielectric constant of 2.9 was used to describe the EMC solvent,^[^ ^57^, ^58^ ^]^ and a Debye length of 3 Å was set to simulate a 1 m condition.

To simulate two systems of NGO functionalized with distinct surface functionalities, the CNG and ENG surfaces were modeled. In the CNG model, a carboxylic acid functional group was attached to the edges of NGO, while in the ENG model, an ethylenediamine functional group was attached to the NGO (Figure S8, Supporting Information). At least 15 Å of a vacuum layer was added in the z‐direction to avoid imaginary interactions between the periodic images. The modeled concentrations of CNG and ENC in the 1 m baseline electrolyte were 44.95 and 46.09 mg mL^−1^, respectively. It was noted that calculations performed under more dilute conditions (e.g., ≈10 mg mL^−1^) using larger supercells yielded nearly identical results, with adsorption energy differences of less than 0.02 eV. Therefore, for computational efficiency, the former supercell was employed.

The adsorption energies of EMC, FEC and Na^+^, and PF_6_^−^ were calculated as follows:

(2) $$\Delta E_{ads}=E_{molecule^{*}}-E_{molecule}-E_{*}$$

where $E_{*},E_{molecule^{*}}$ and E_*molecule*_ correspond to the DFT energies of the bare NGO/CNG/ENG, the molecule‐adsorbed NGO/CNG/ENG, and the isolated molecule in the 25 × 25 × 25 Å^3^ cell, respectively. Various adsorption configurations were tested, and the most stable structures were chosen for further analysis. To account for the charges of the molecules, the number of electrons (n_elect_) was adjusted accordingly. Specifically, one electron was removed for Na^+^, while one electron was added for PF_6_^−^.

## Conflict of Interest

The authors declare no conflict of interest.

## Supporting information



Supporting Information

## Data Availability

The data that support the findings of this study are available from the corresponding author upon reasonable request.
