# Supplementary material for: Dual‐Ionic Weakly Solvating Electrolyte Design Enables Efficient Fast‐Cycling of High‐Voltage Anion Shuttle Batteries
Source: Adv Sci (Weinh). 2025 Jul 11;12(39):e05982. doi: 10.1002/advs.202505982 (PMC12533411; doi:10.1002/advs.202505982)
Supplement: Supplementary file 1 — Supporting Information [file ADVS-12-e05982-s001.docx]

Supporting Information

Dual-ionic Weakly Solvating Electrolyte Design Enables Efficient Fast-Cycling of High-Voltage Anion Shuttle Batteries

*Jieun Kang, Inhui Lee, Gwonho Yu, Jin Jun Heo, Yuri Choi, Sangyeop Lee, Sungho Kim, Dongjoo Kim, Jungki Ryu, Seoin Back,* Soojin Park,* and Jaegeon Ryu**


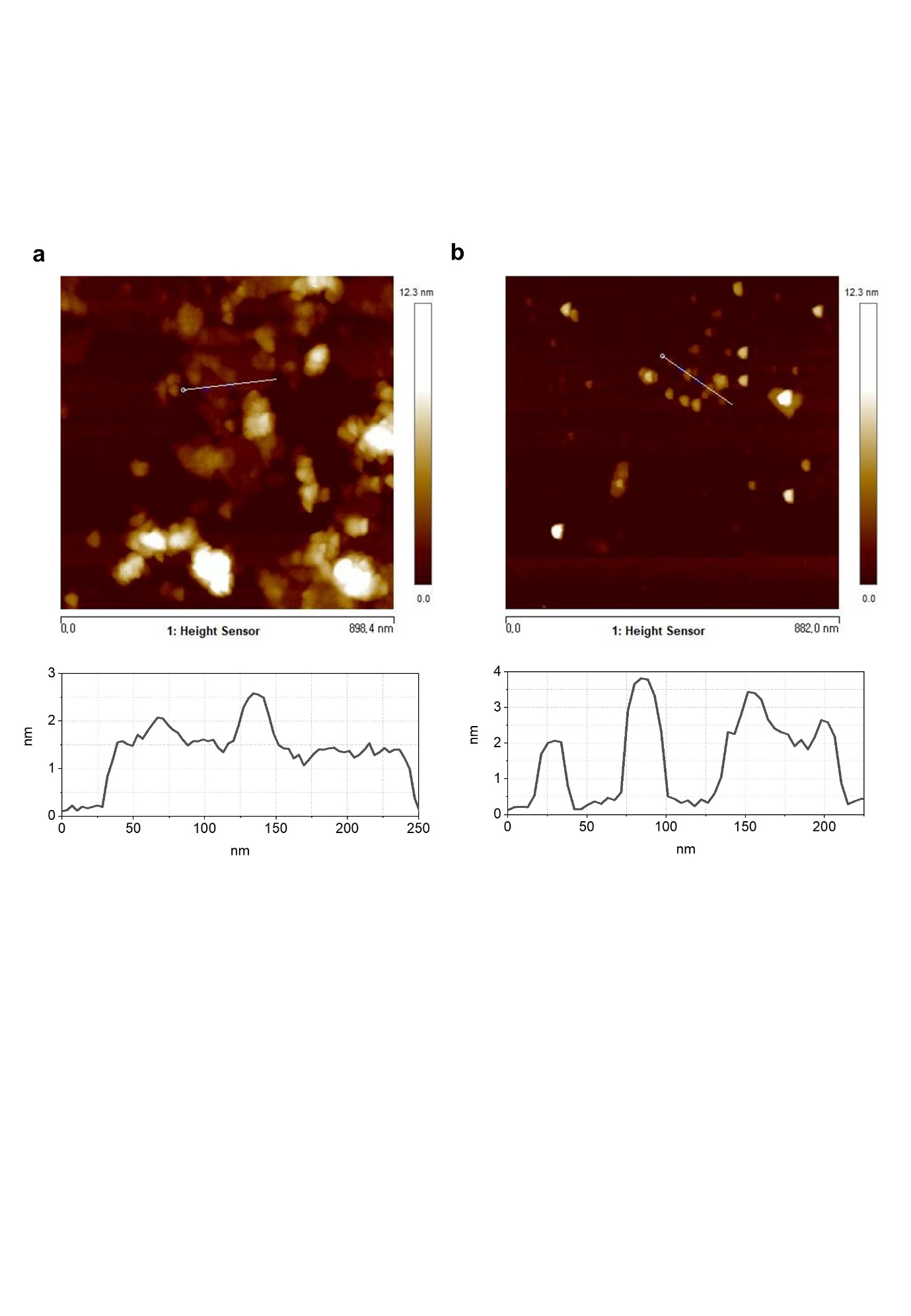


**Figure S1**. Atomic force microscopy (AFM) images of (a) CNG and (b) ENG. The graphs below display the dimensions of the NGOs.


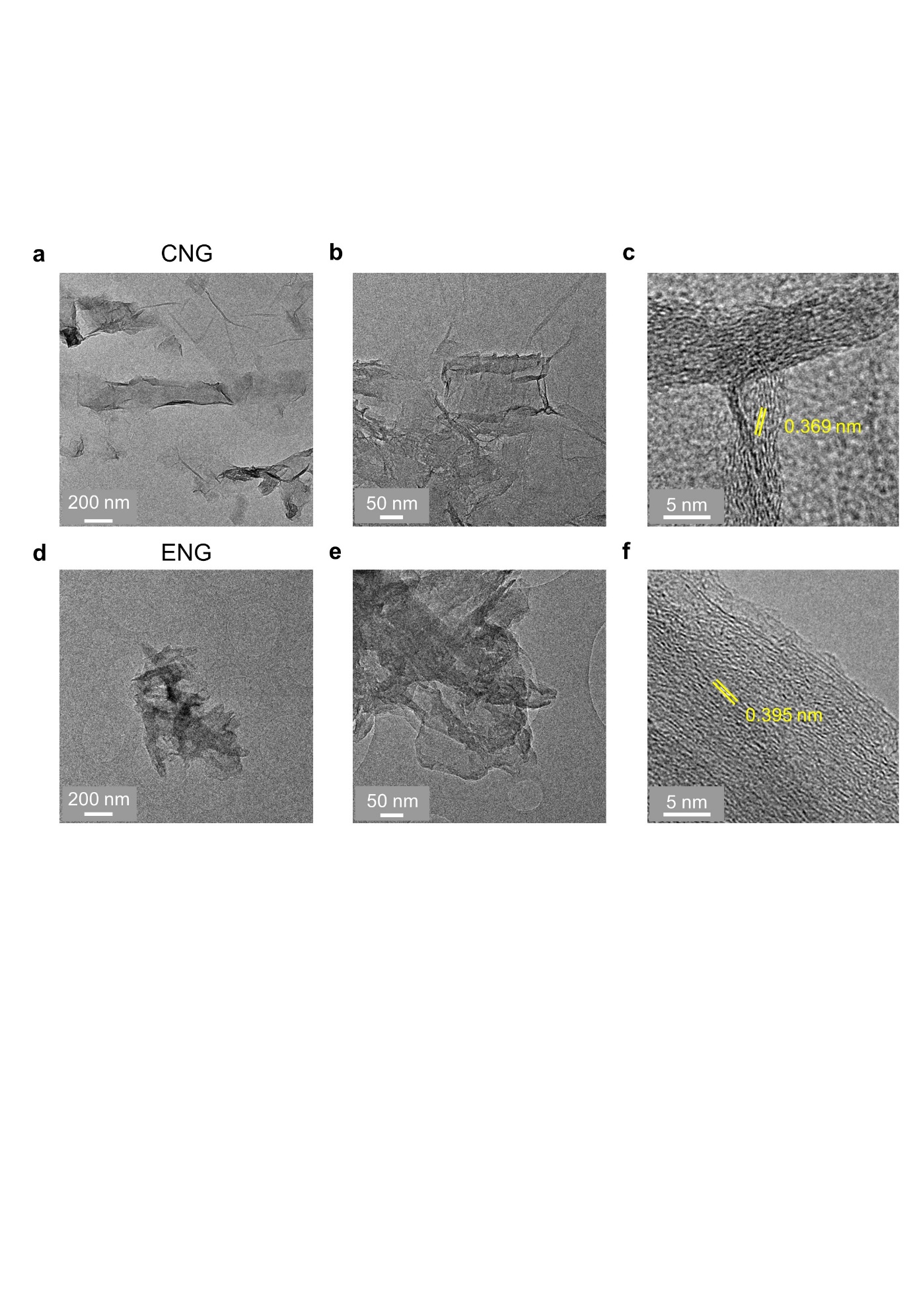


**Figure S2.** Transmission electron microscopy (TEM) images of synthesized (a-c) CNG and (d-f) ENG at various magnifications.


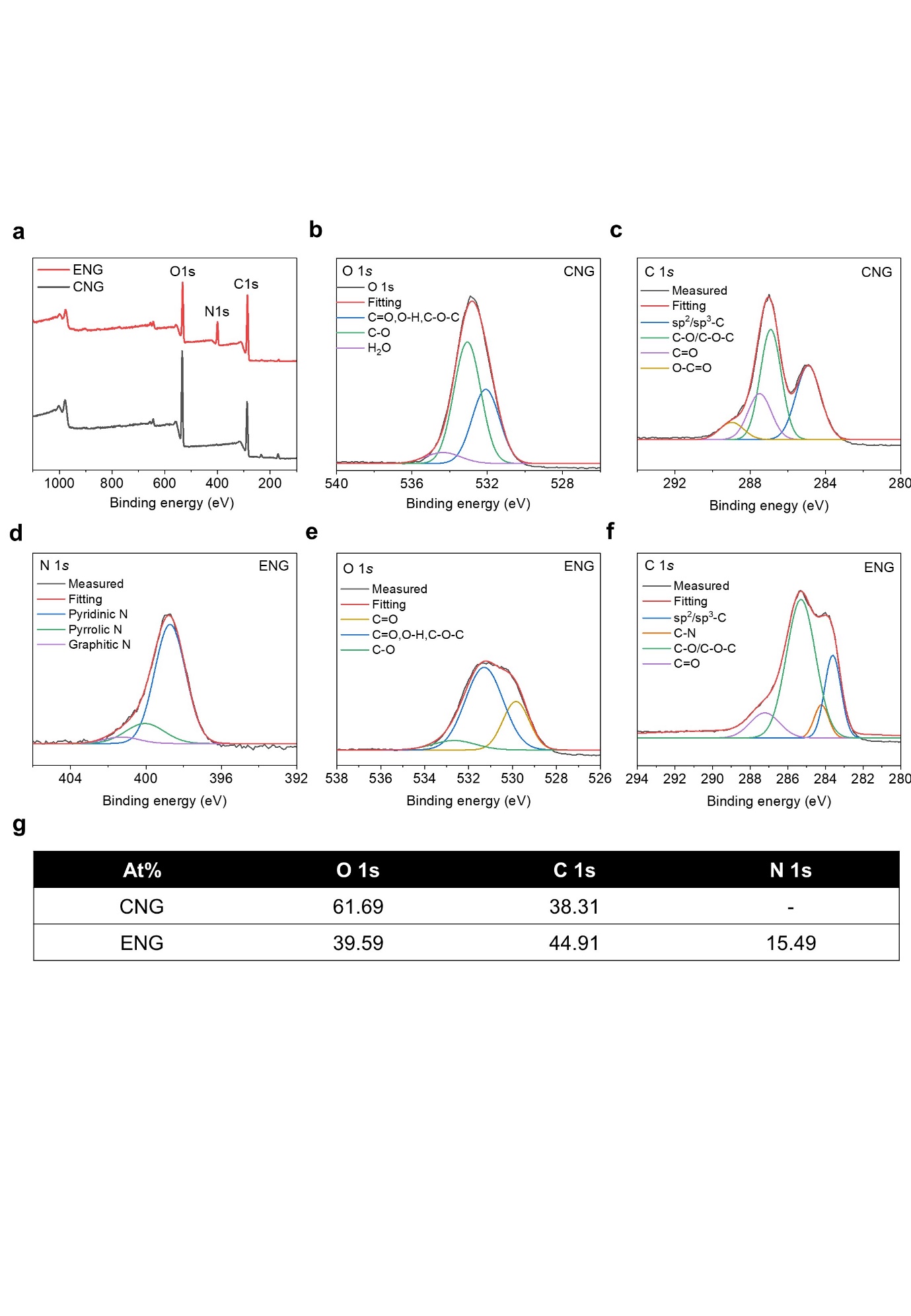


**Figure S3**. (a) XPS profiles of NGOs. High-resolution XPS spectra of (b) O 1s and (c) C 1s for CNG, and (d) N 1s, (e) O 1s, and (f) C 1s for ENG. (g) Atomic composition of NGOs determined by XPS analysis.


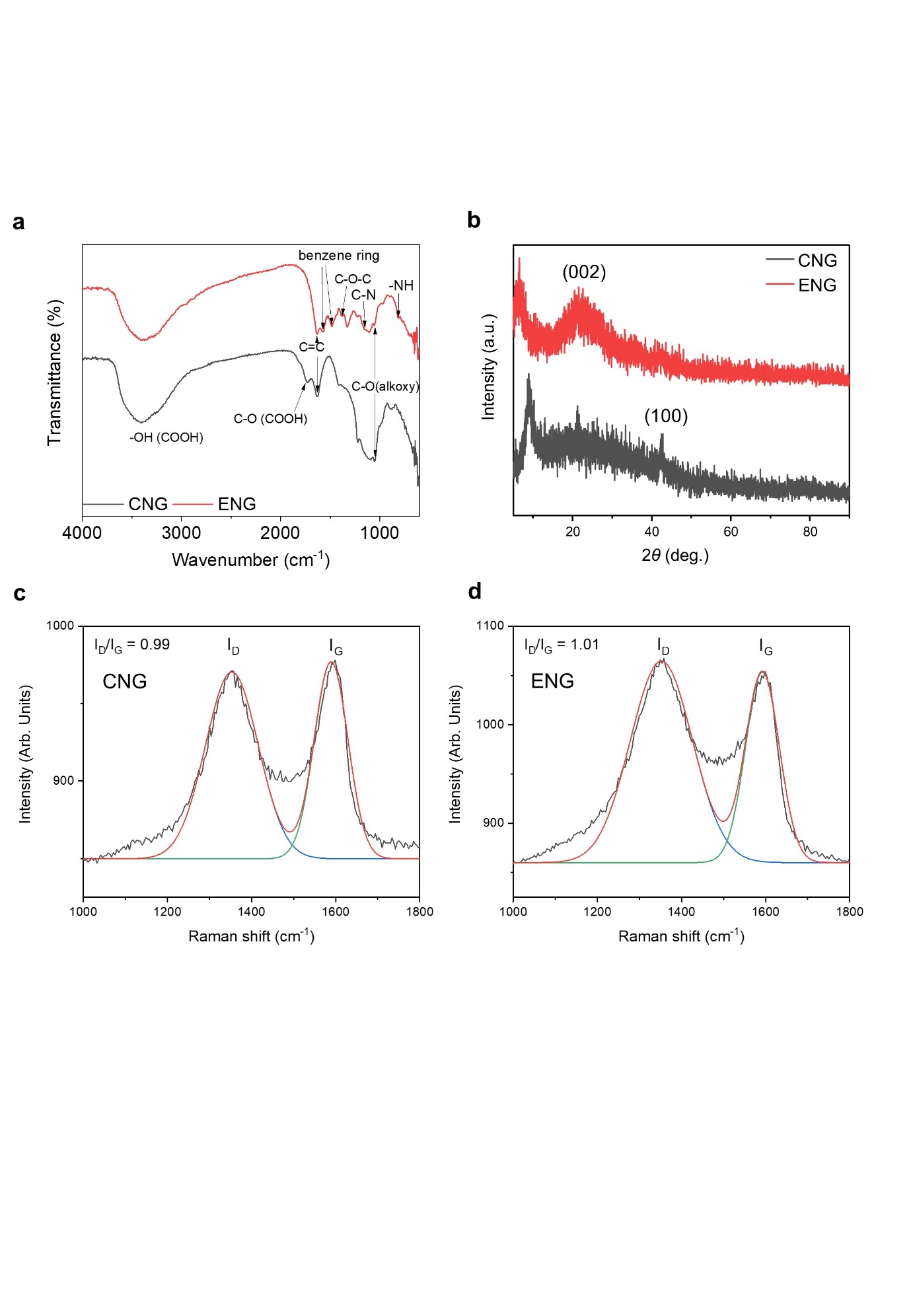


**Figure S4**. (a) FT-IR spectra and (b) XRD patterns of CNG and ENG. Raman spectrum of (c) CNG and (d) ENG.


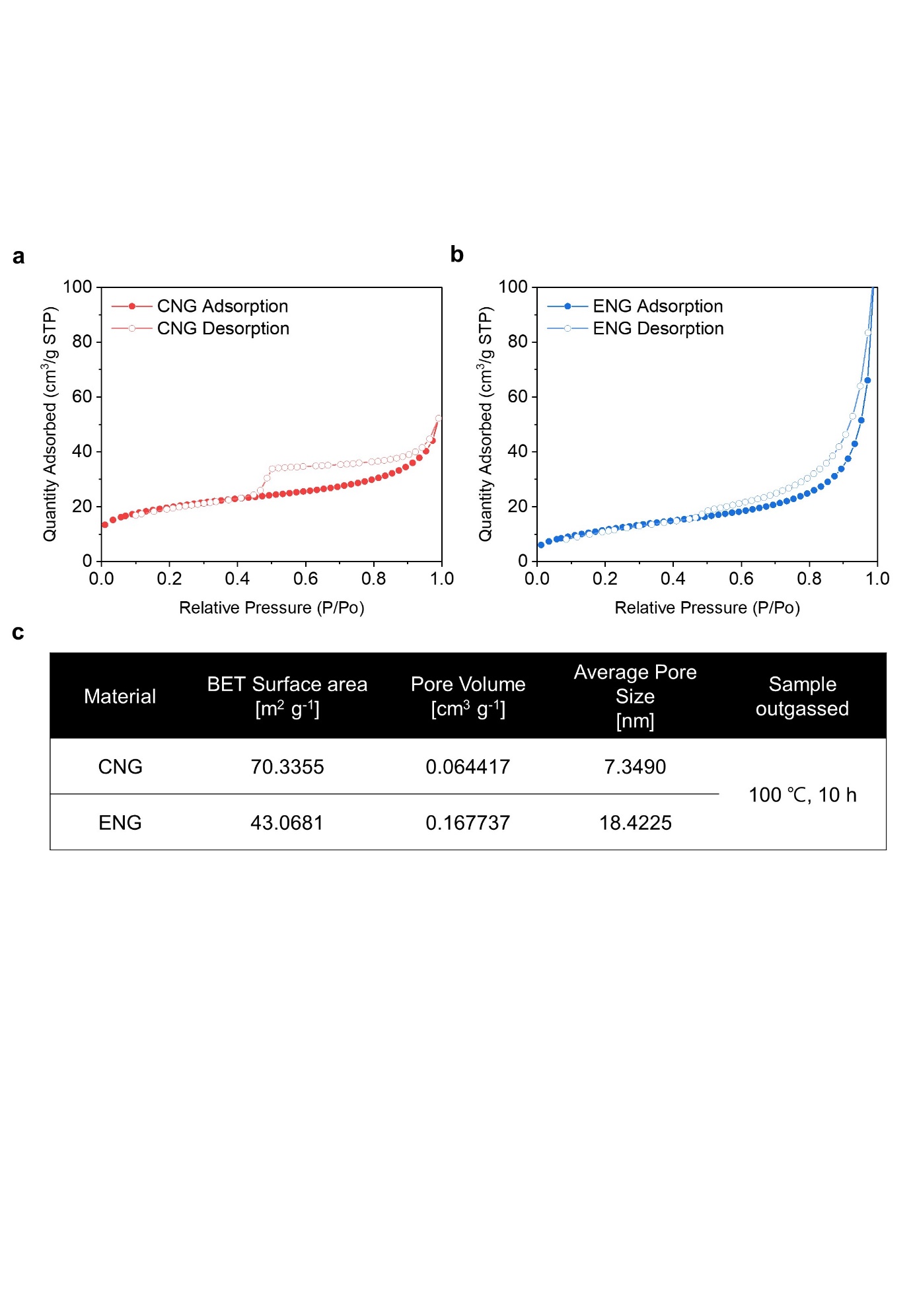


**Figure S5**. BET graphs of (a) CNG and (b) ENG. (c) Summary of BET surface area, pore volume, and average pore size for CNG and ENG.

**
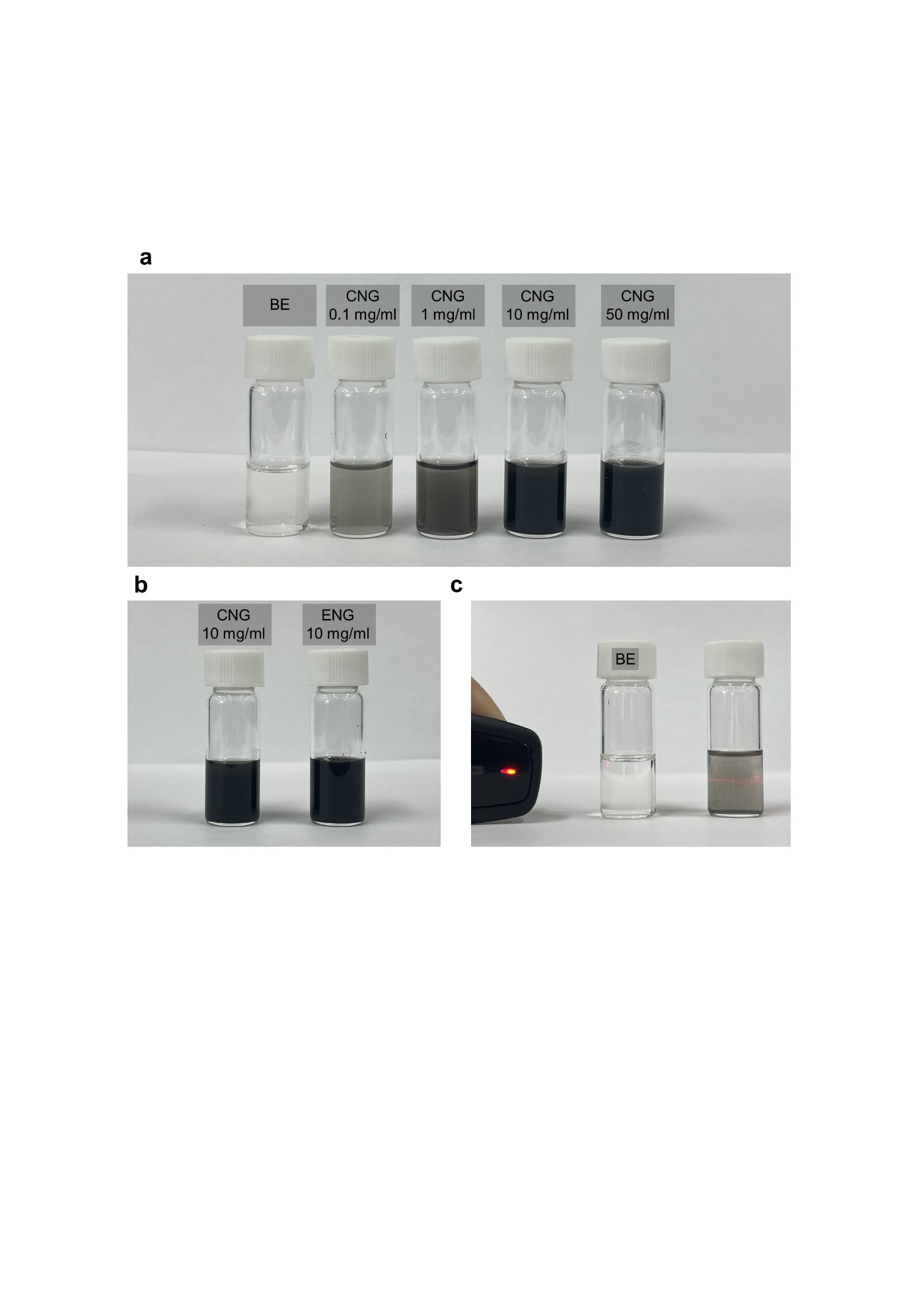
**

**Figure S6**. (a) Photographs of electrolytes with varying CNG concentrations. (b) Comparison of electrolytes based on 10 mg/mL CNG and ENG. (c) Demonstration of the colloidal properties of the CNG-based electrolyte, showing the Tyndall effect under laser illumination.


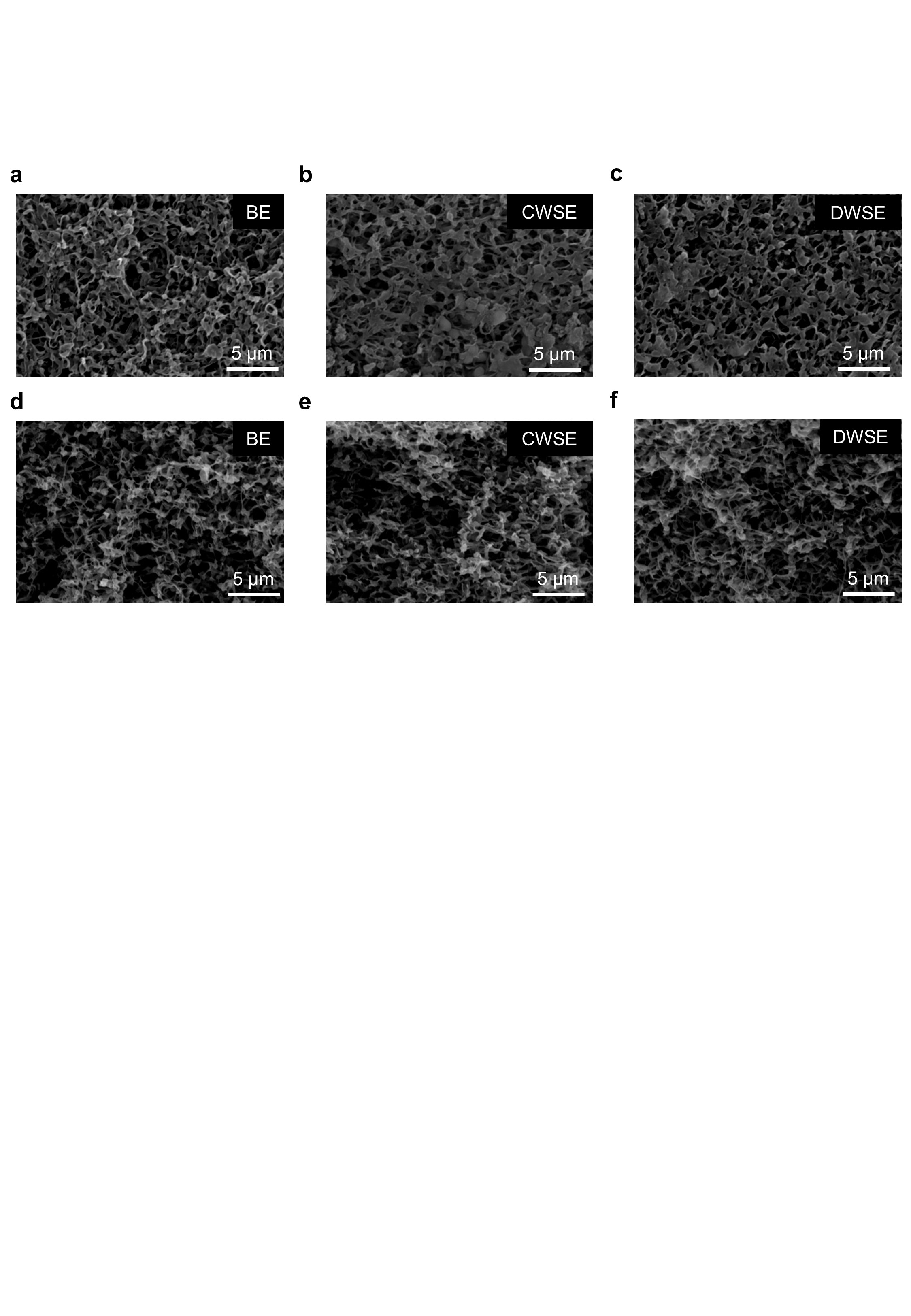


**Figure S7.** Top-view SEM images of separators after soaking in (a) BE, (b) CWSE, and (c) DWSE, followed by drying. Cross-sectional SEM images of separators corresponding to (d) BE, (e) CWSE, and (f) DWSE.


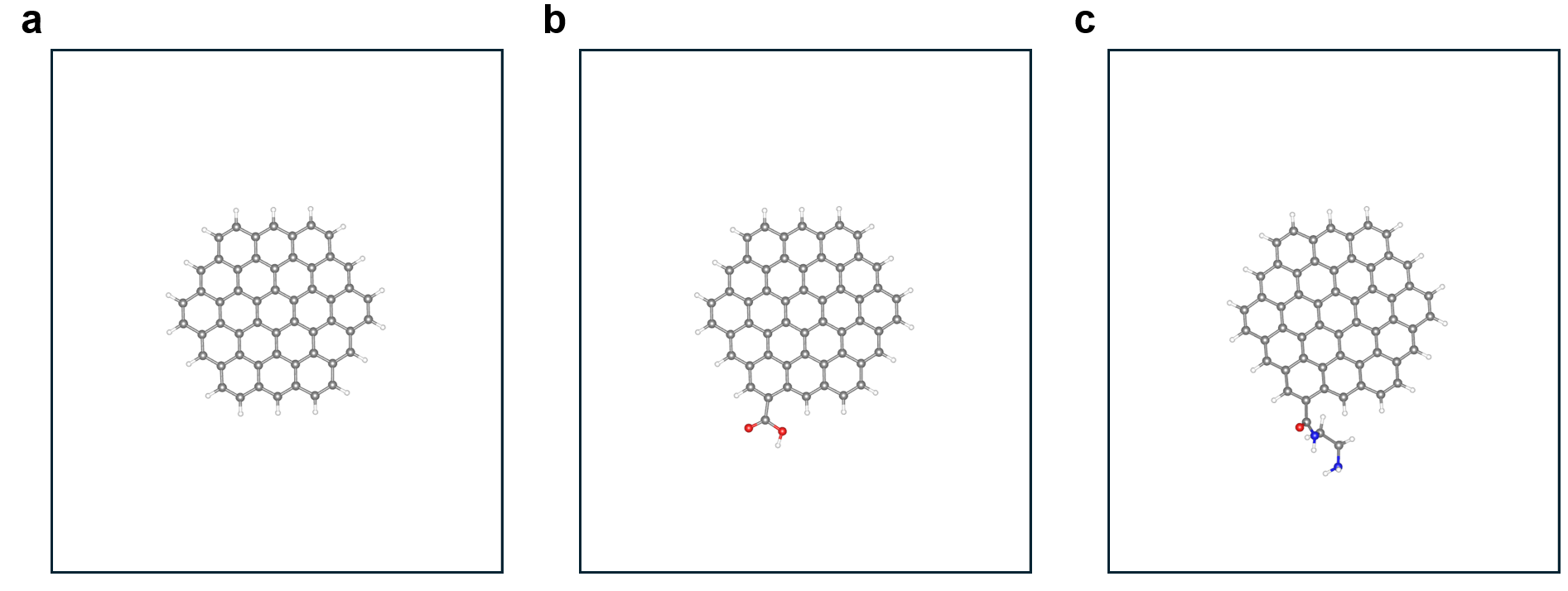


**Figure S8**. The optimized structure of (a) NGO, (b) CNG and (c) ENG. Color codes: red (O), gray (C), white (H) and blue (N).

**
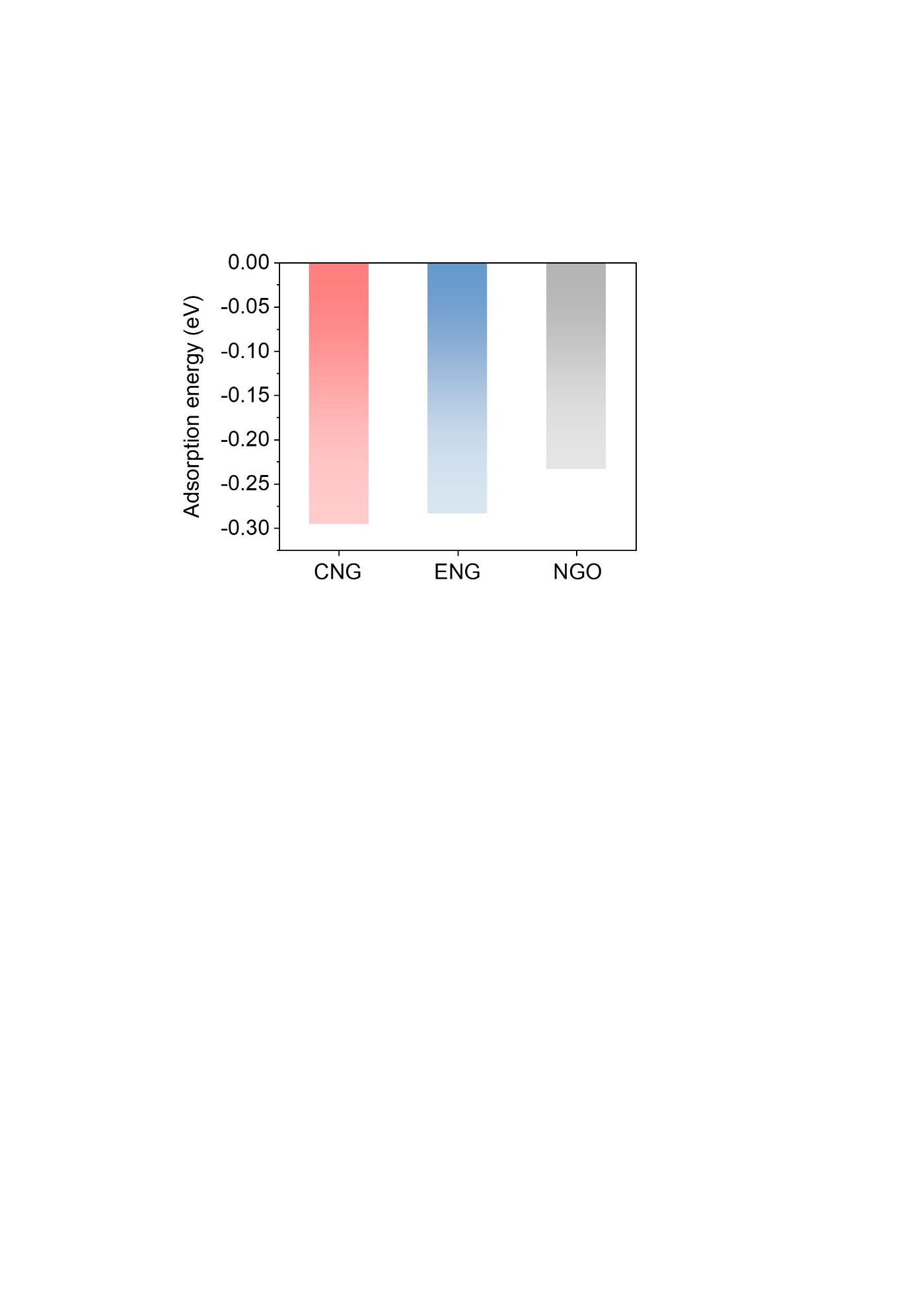
**

**Figure S9**. Binding energy of CNG, ENG, and NGO with EMC, calculated using DFT.


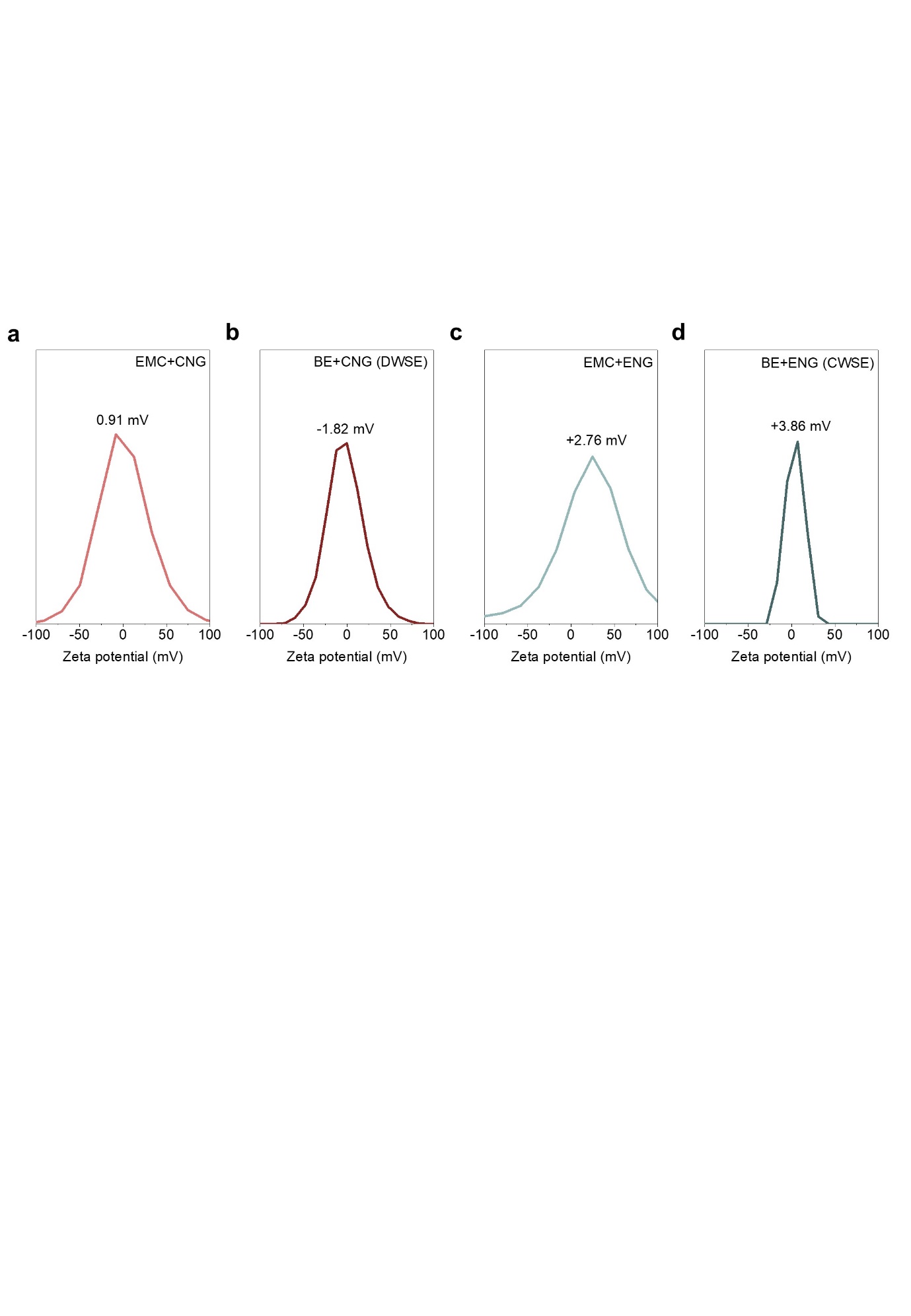


**Figure S10**. Zeta potential measurements of (a) CNG dispersed in EMC, (b) CNG dispersed in BE, (c) ENG dispersed in EMC, and (d) ENG dispersed in BE.


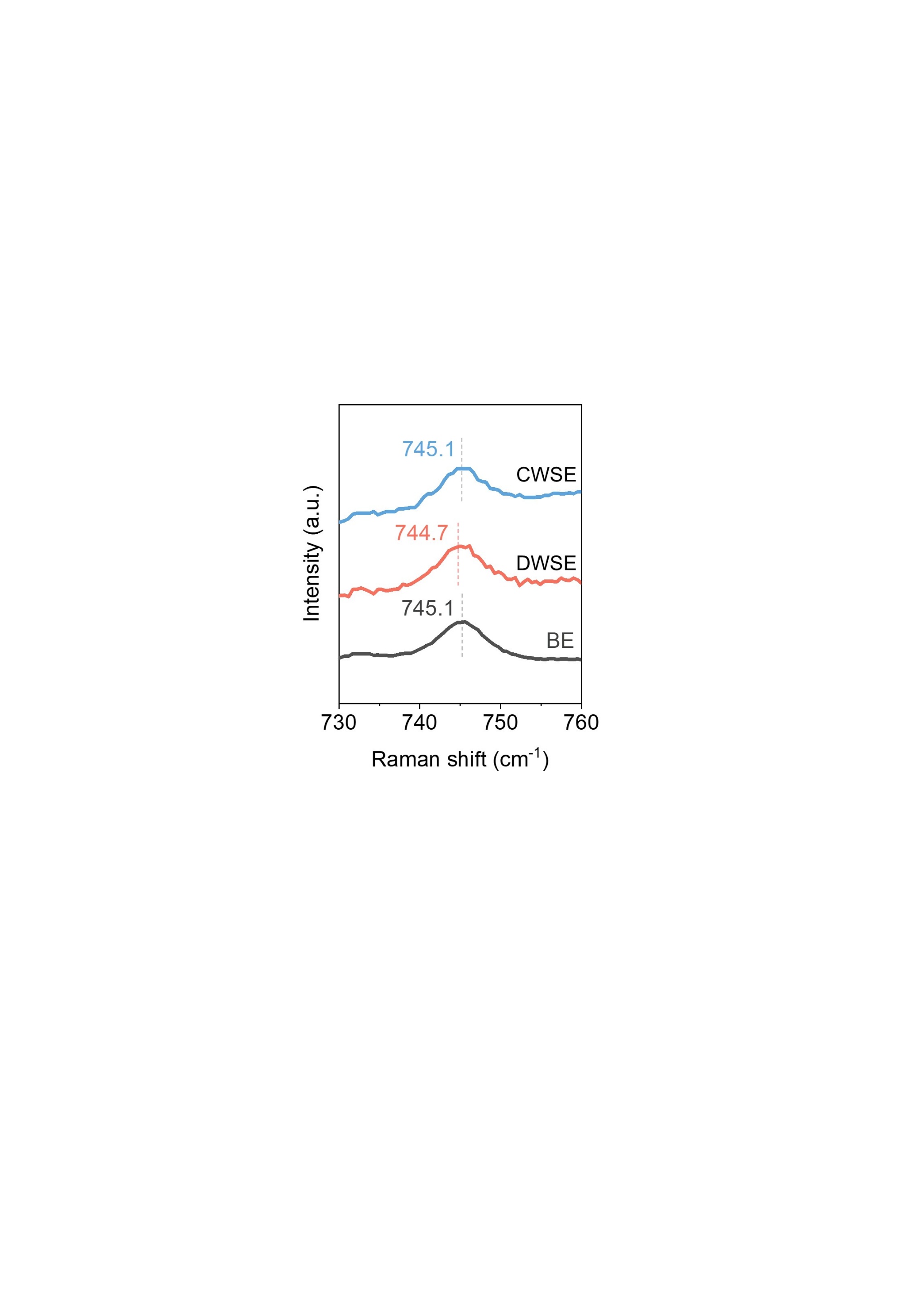


**Figure S11**. Raman spectra of bulk electrolytes.


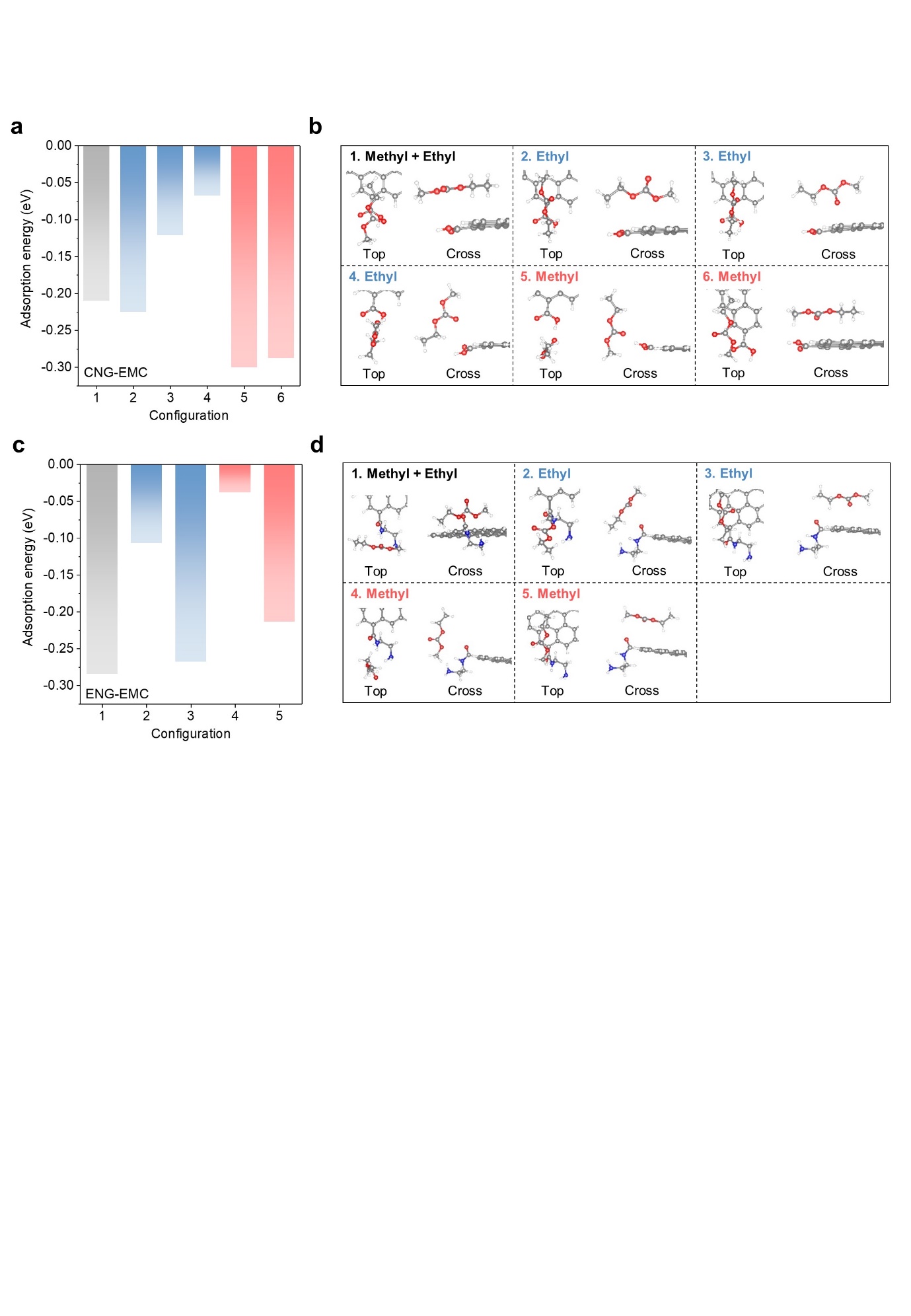


**Figure S12.** Adsorption energy of EMC on (a) CNG and (c) ENG for various adsorption configurations. (b) and (d) show the corresponding adsorption configurations depicted in (a) and (c). For CNG, interactions with the methyl group of EMC exhibit particularly strong adsorption energies (configurations 5 and 6). For ENG, the most stable interaction occurs when both the methyl and ethyl groups of EMC are involved (configuration 1).


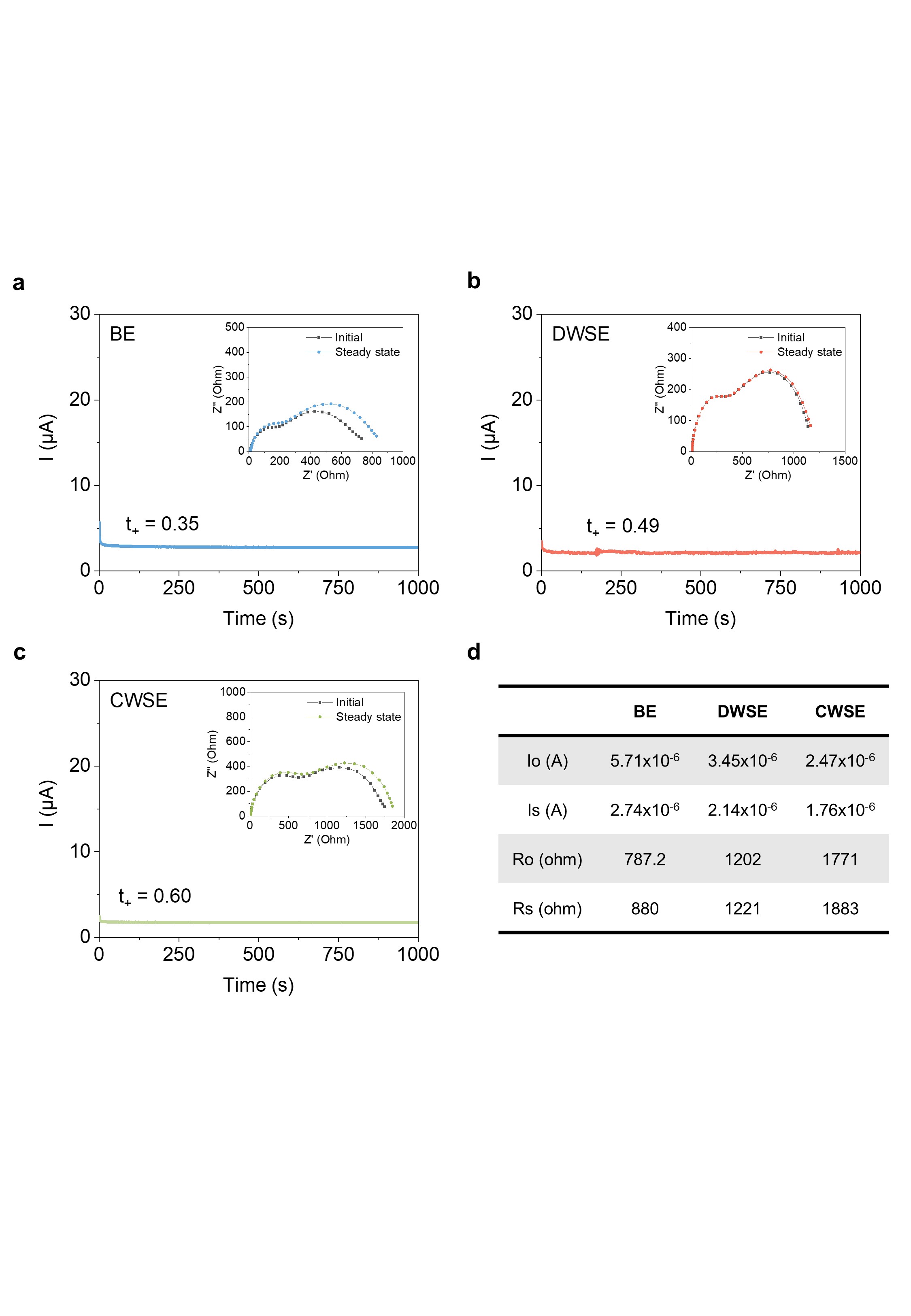


**Figure S13.** Time-dependence current response to 10 mV polarization and impedance spectra of (a) BE, (b) DWSE, and (c) CWSE. (d) Summary table of the parameters obtained from the experiments in (a-c).


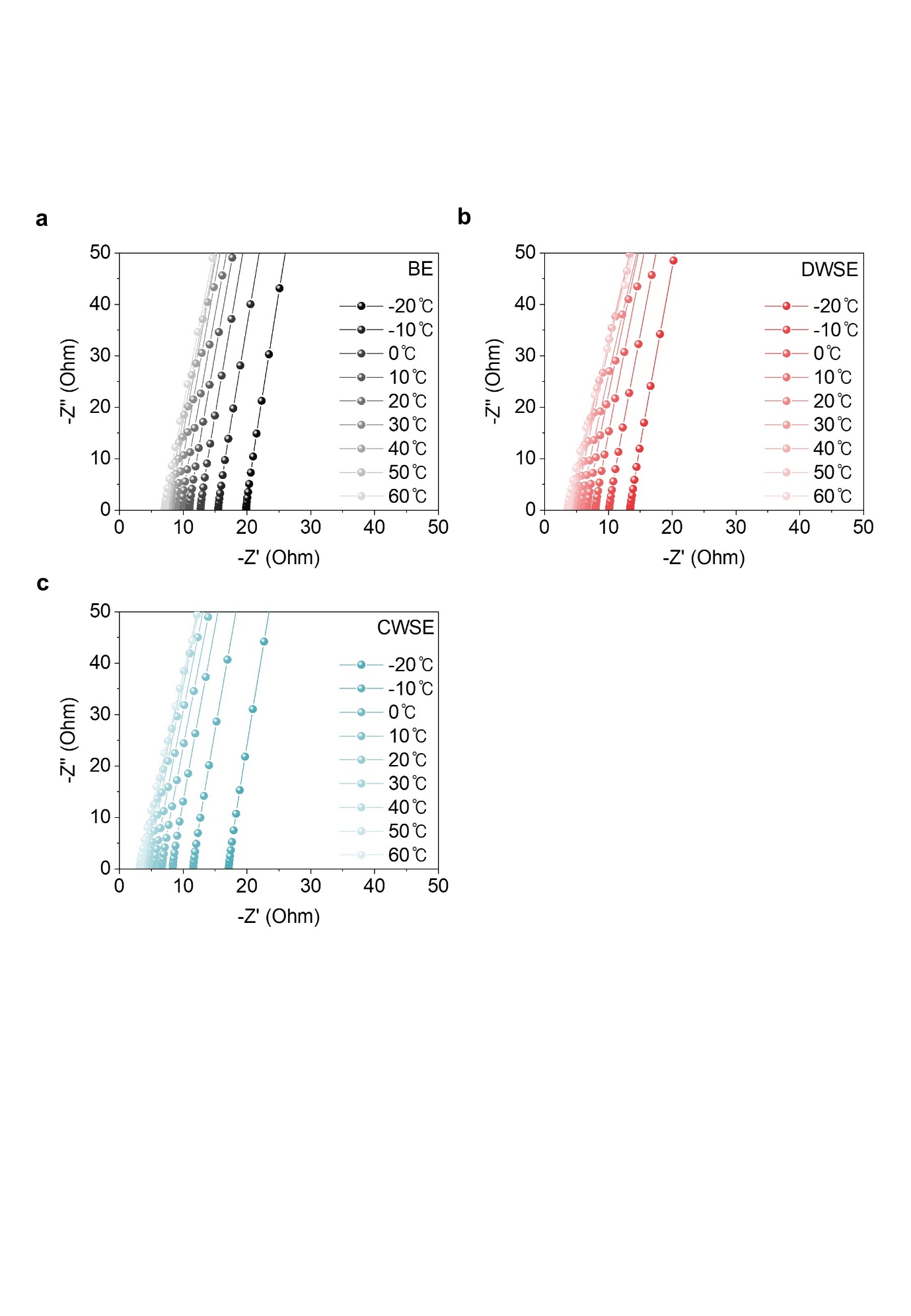


**Figure S14.** EIS spectra of (a) BE, (b) DWSE, and (c) CWSE at varying temperatures from -20 ℃ to 60 ℃.


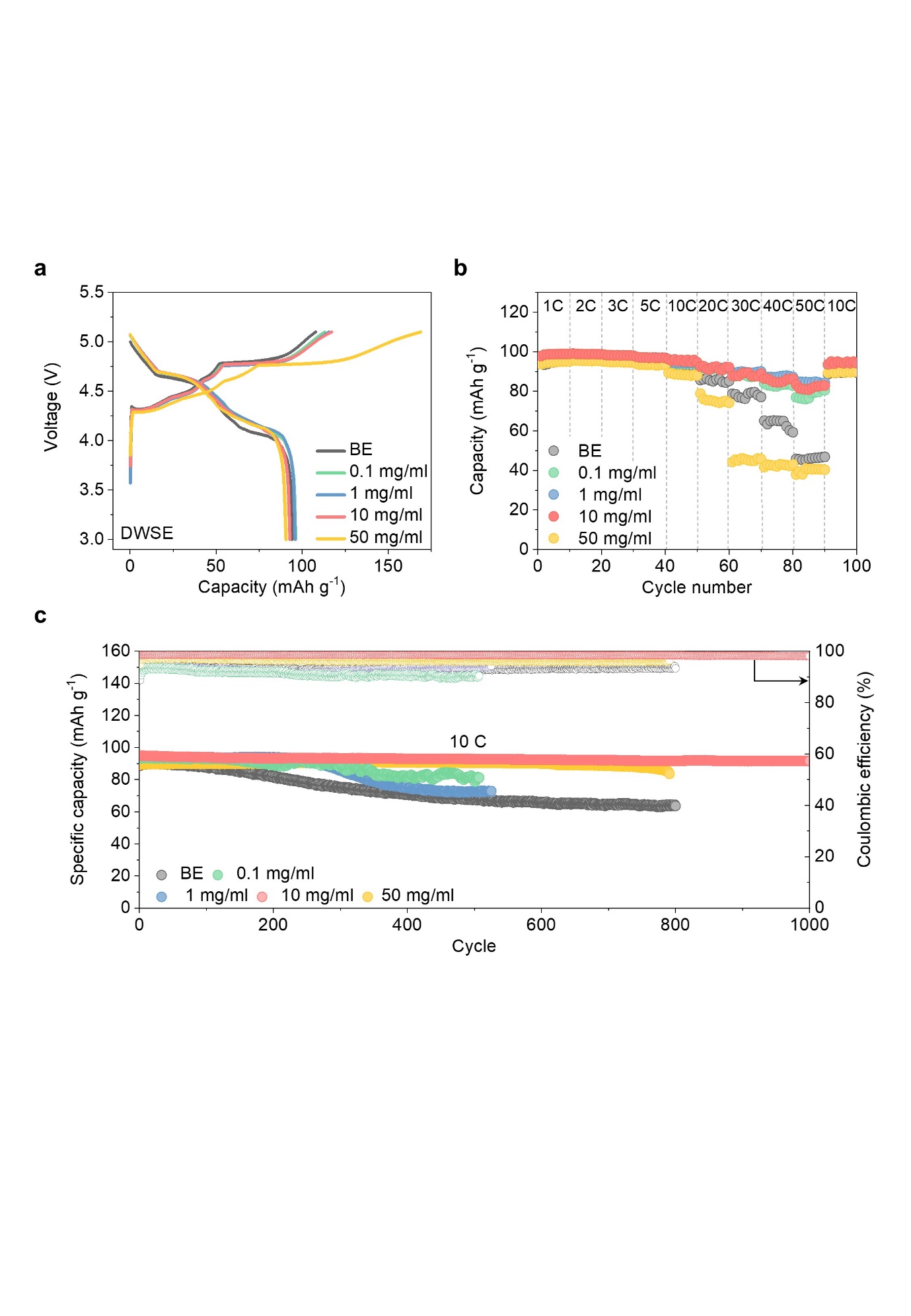


**Figure S15.** DIB cell performances with varying CNG concentrations in the electrolyte (0.1, 1, 10, and 50 mg mL^-1^). (a) Voltage profiles at 1 C. (b) Comparison of reversible capacities from 1 C to 50 C. (c) Long-term cycling stability at 10 C.


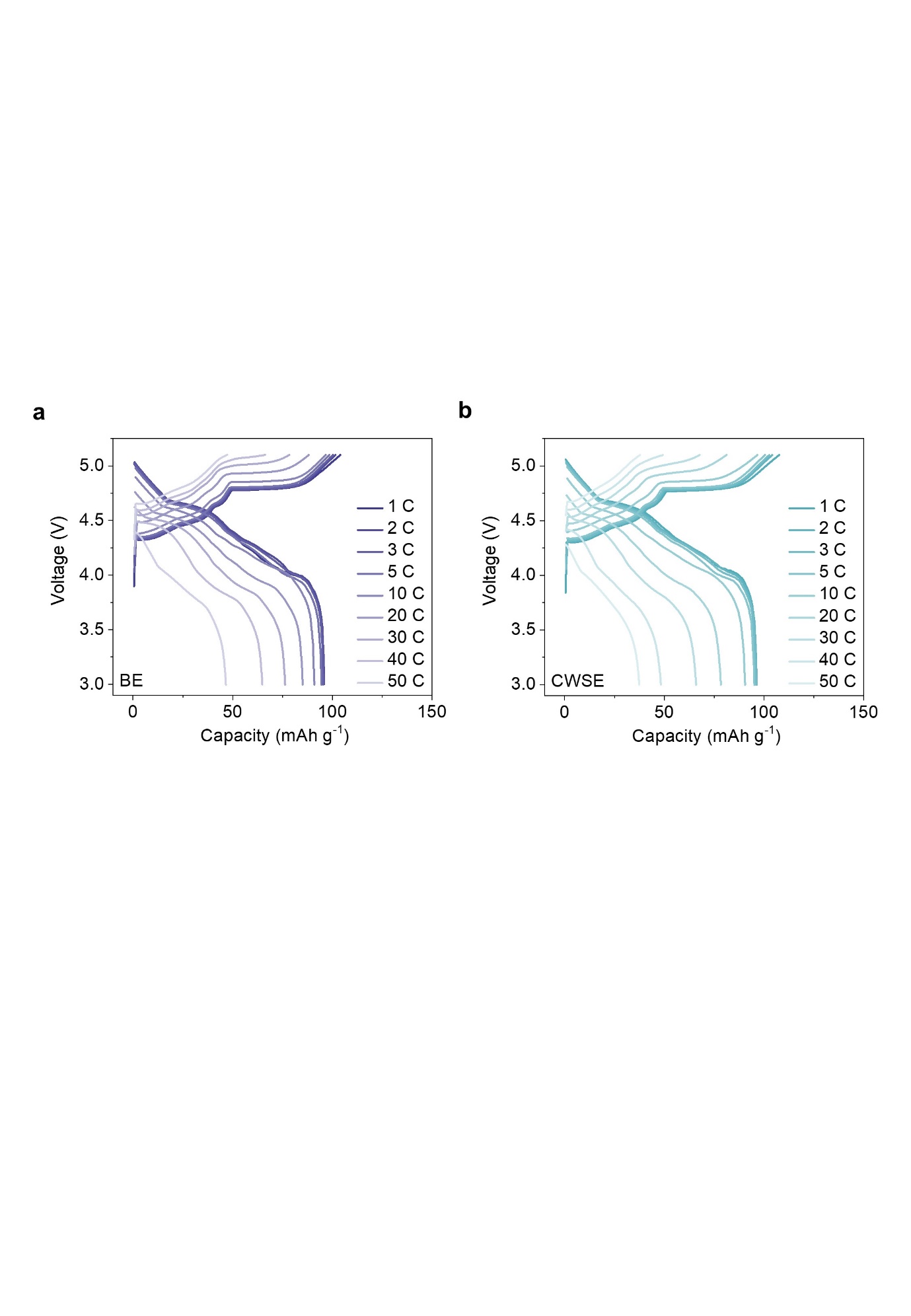


**Figure S16.** Voltage profiles of DIB cells using (a) BE and (b) CWSE electrolytes at current rates ranging from 1 C to 50 C.


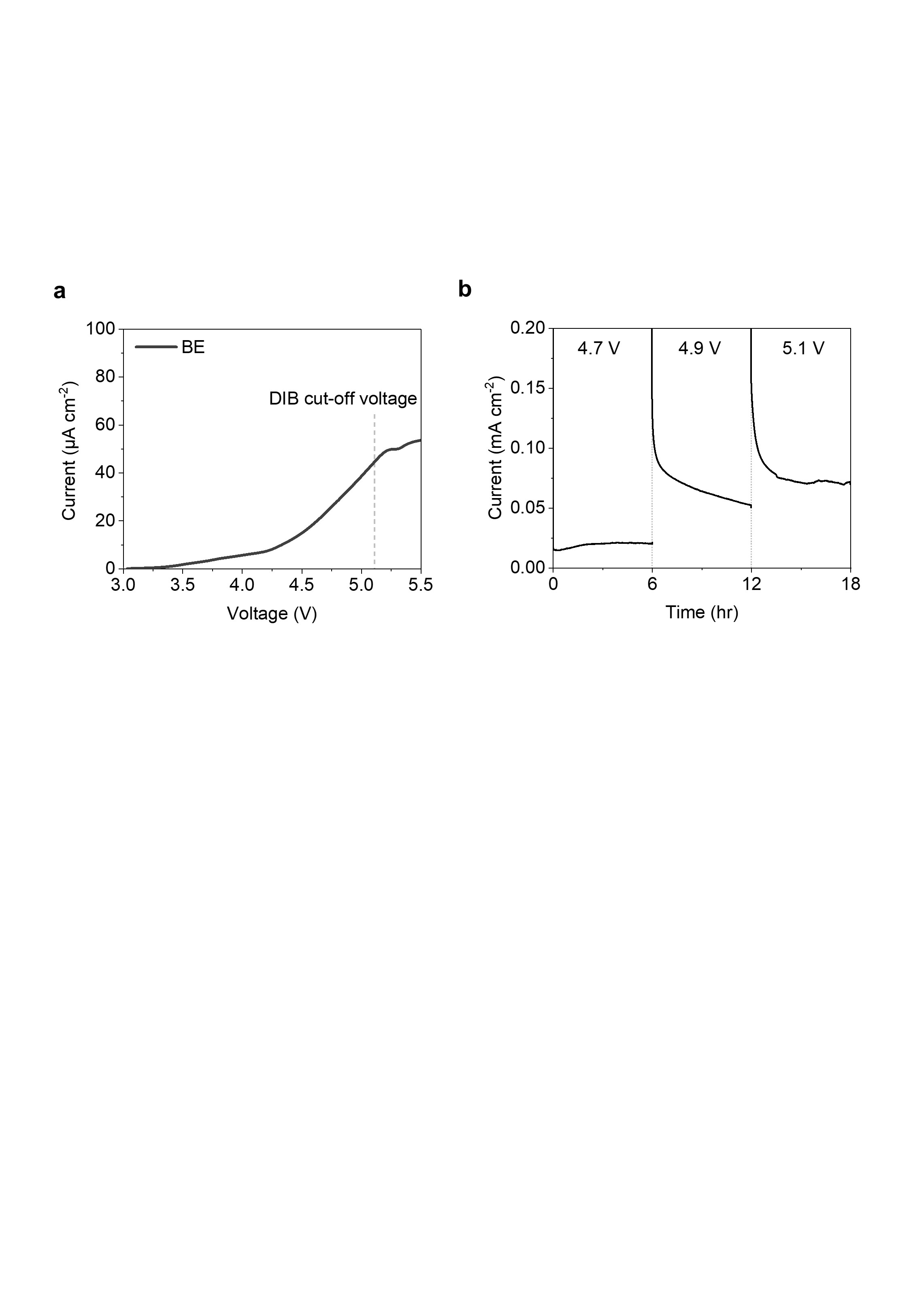


**Figure S17.** (a) Linear sweep voltammetry measurement of BE. The scan rate utilized was 1 mV s^-1^ and the potentials were shifted with reference to Na/Na^+^. (b) Electrochemical floating analysis of BE.


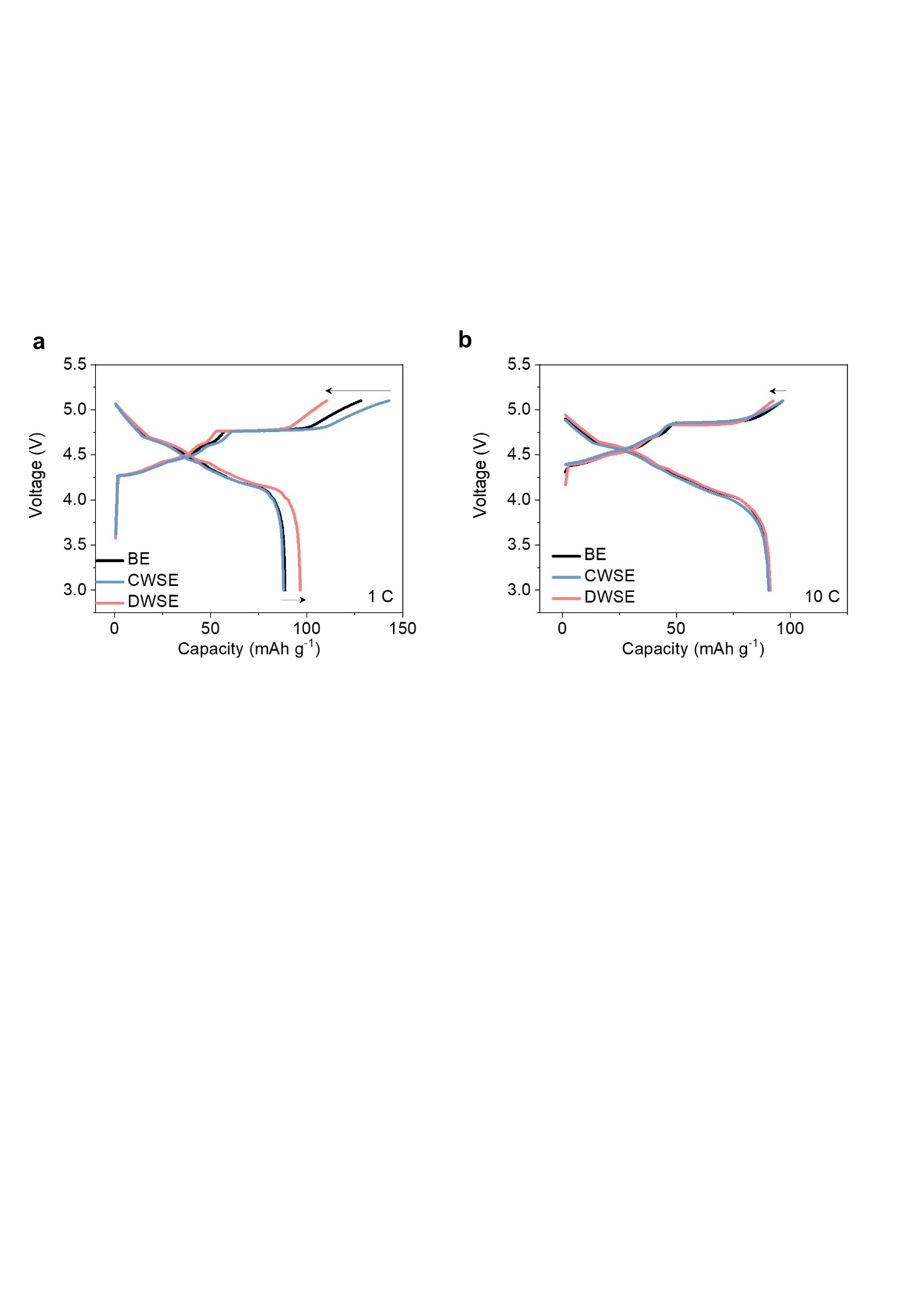


**Figure S18.** Comparison of voltage profiles for DIB cells using BE, CWSE, and DWSE electrolytes at (a) 1 C and (b) 10 C.


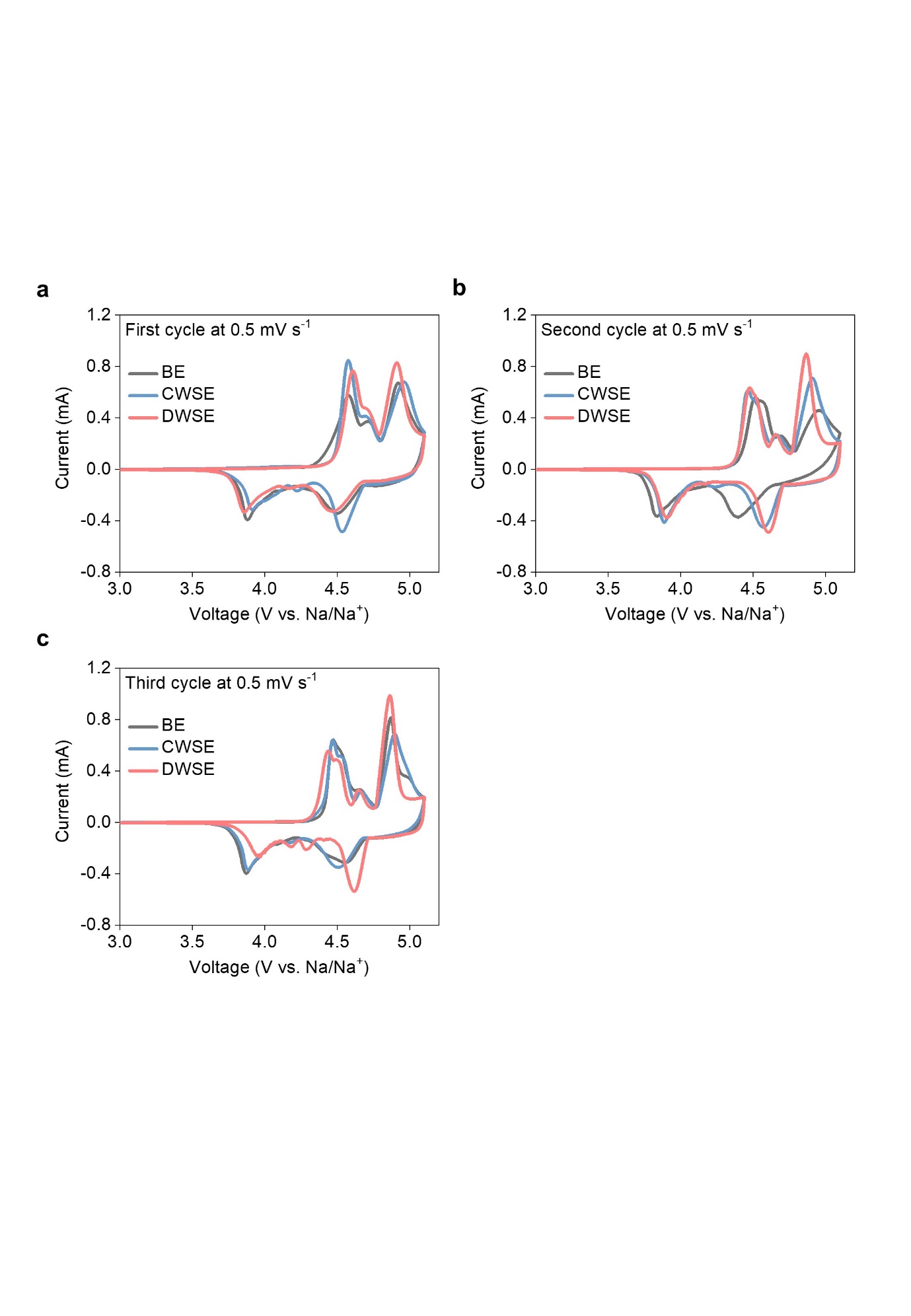


**Figure S19.** Cyclic voltammetry analysis at 0.5 mV s^-1^ for DIB cells using BE, CWSE, and DWSE electrolytes during the (a) first cycle, (b) second cycle, and (c) third cycle.


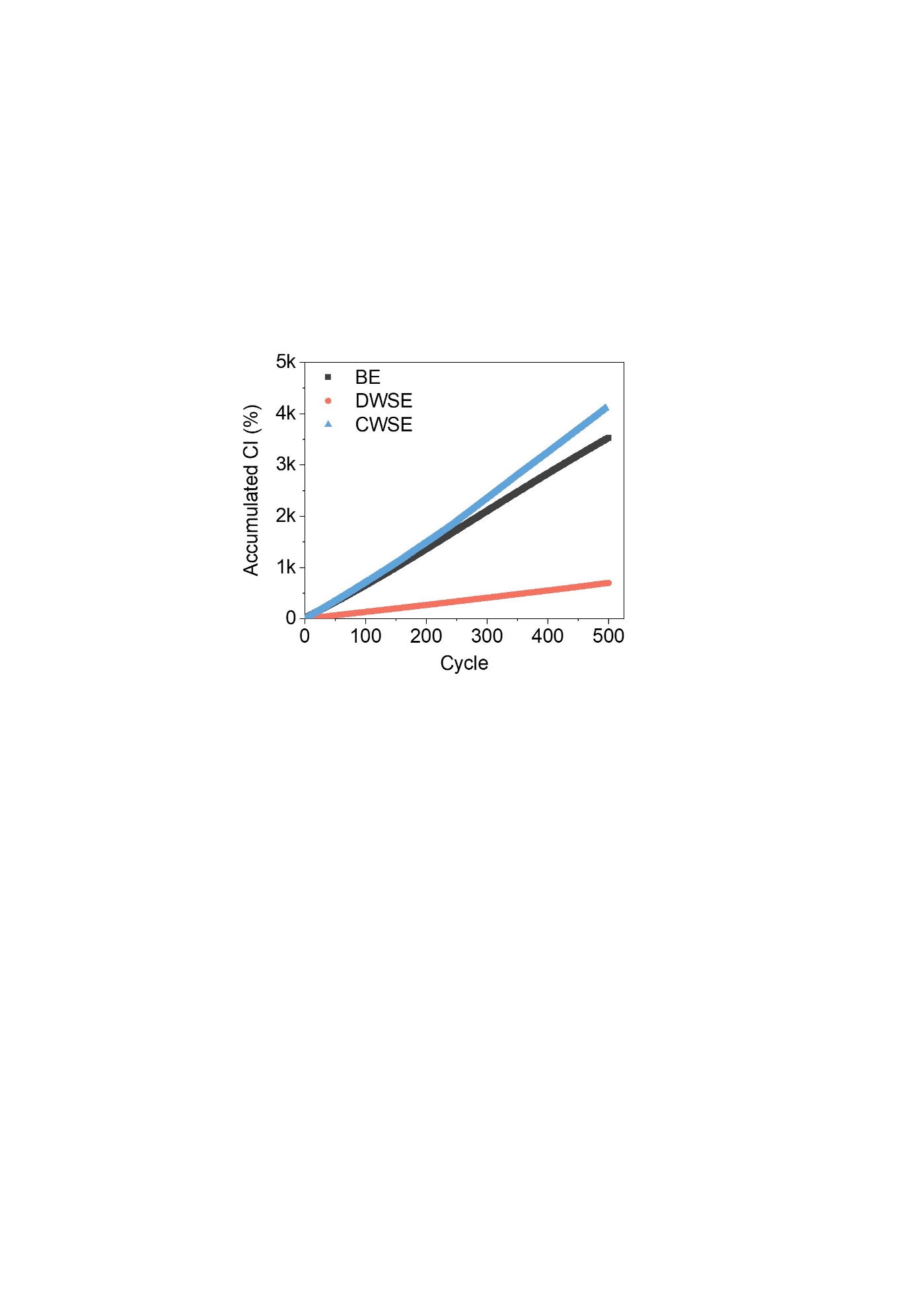


**Figure S20.** Comparison of accumulated Coulombic inefficiency (CI) over cycles for DIB cells using BE, DWSE, and CWSE electrolytes.

**
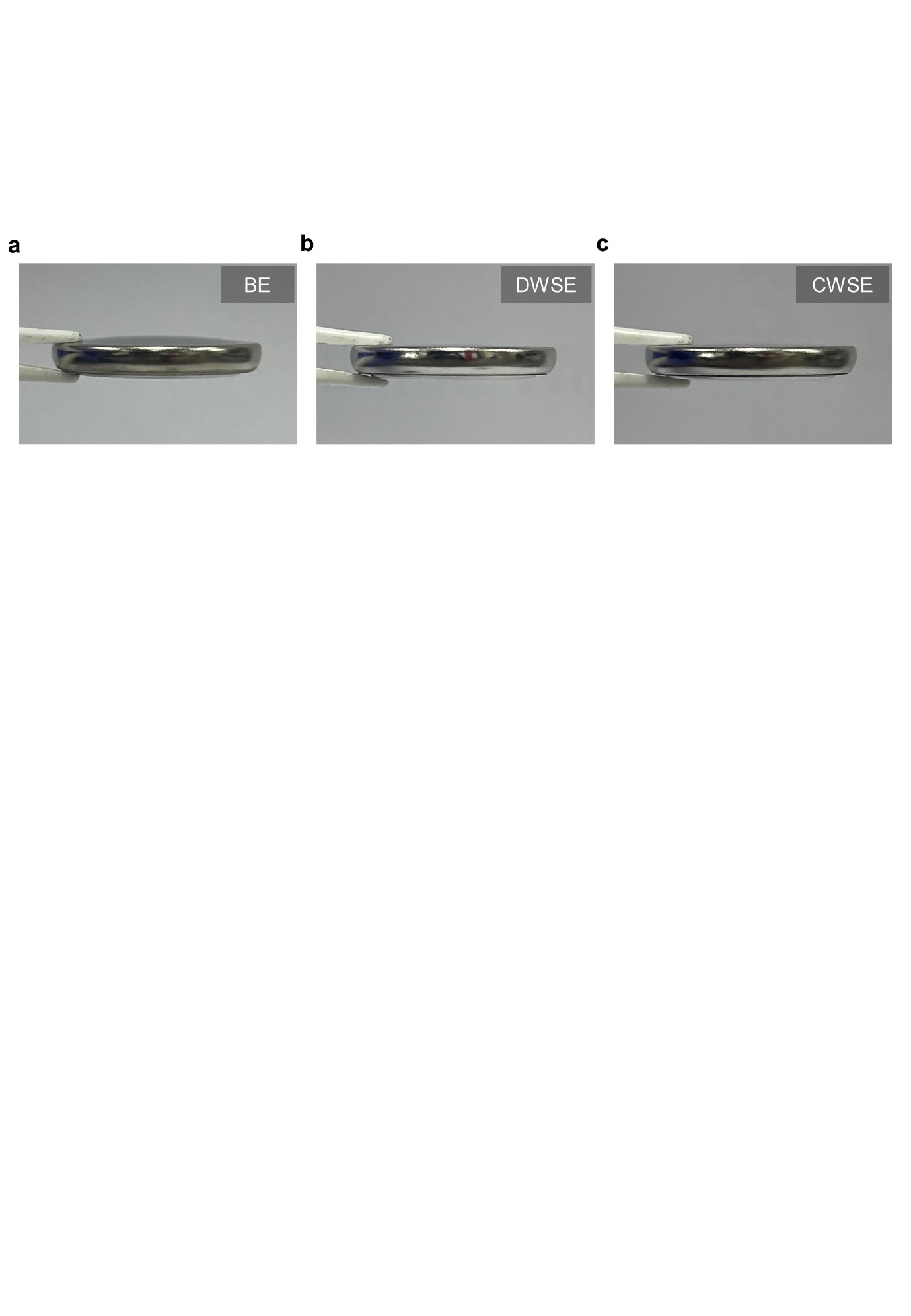
**

**Figure S21.** Photographs showing the extent of swelling in 2032-type coin cells using (a) BE, (b) DWSE, and (c) CWSE electrolytes after 500 cycles.

**
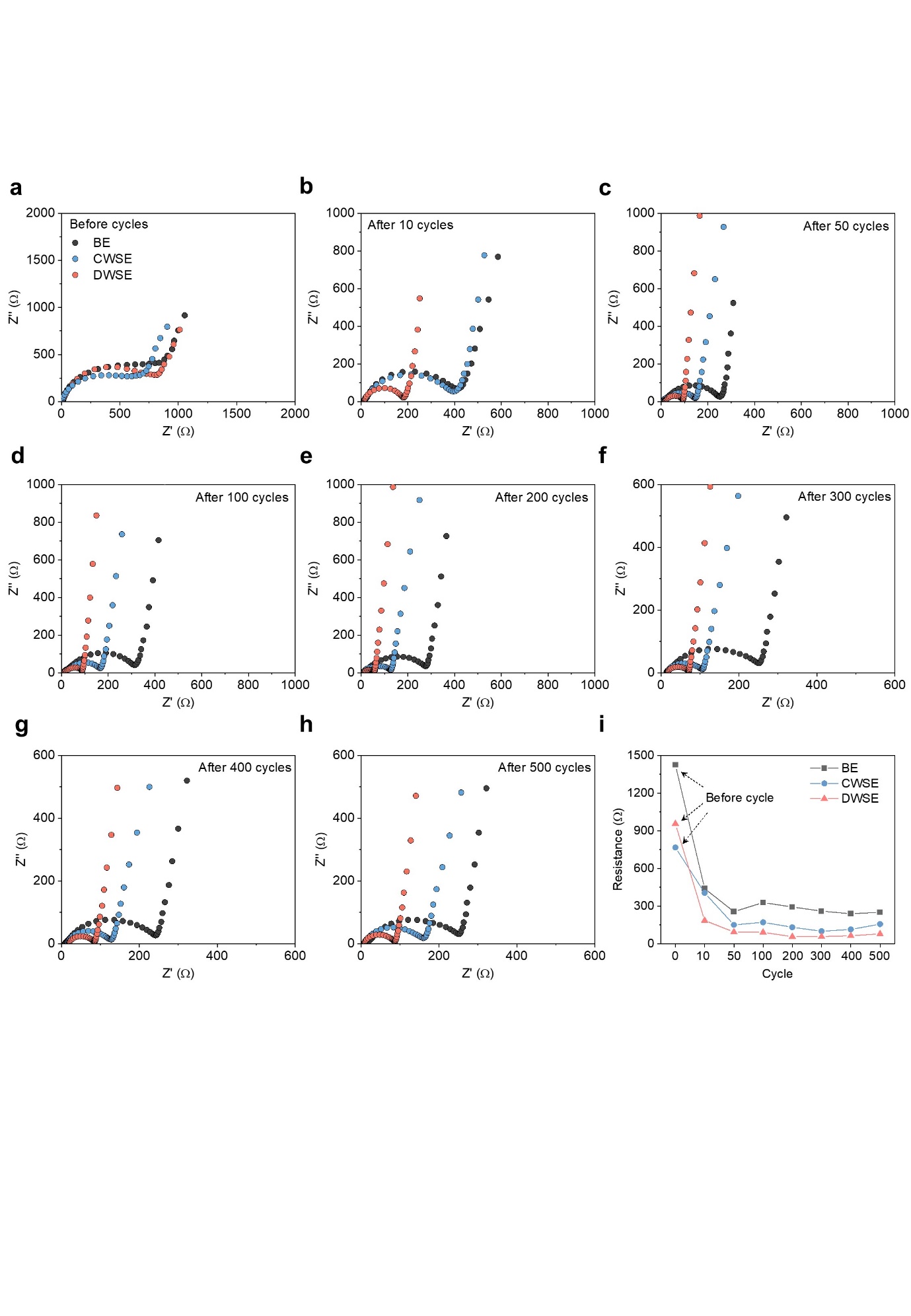
**

**Figure S22.** (a-h) Ex situ EIS spectra of DIB cells using the three electrolytes at different cycling stages. (i) Comparison of resistance values obtained from the EIS spectra as a function of the cycle number.


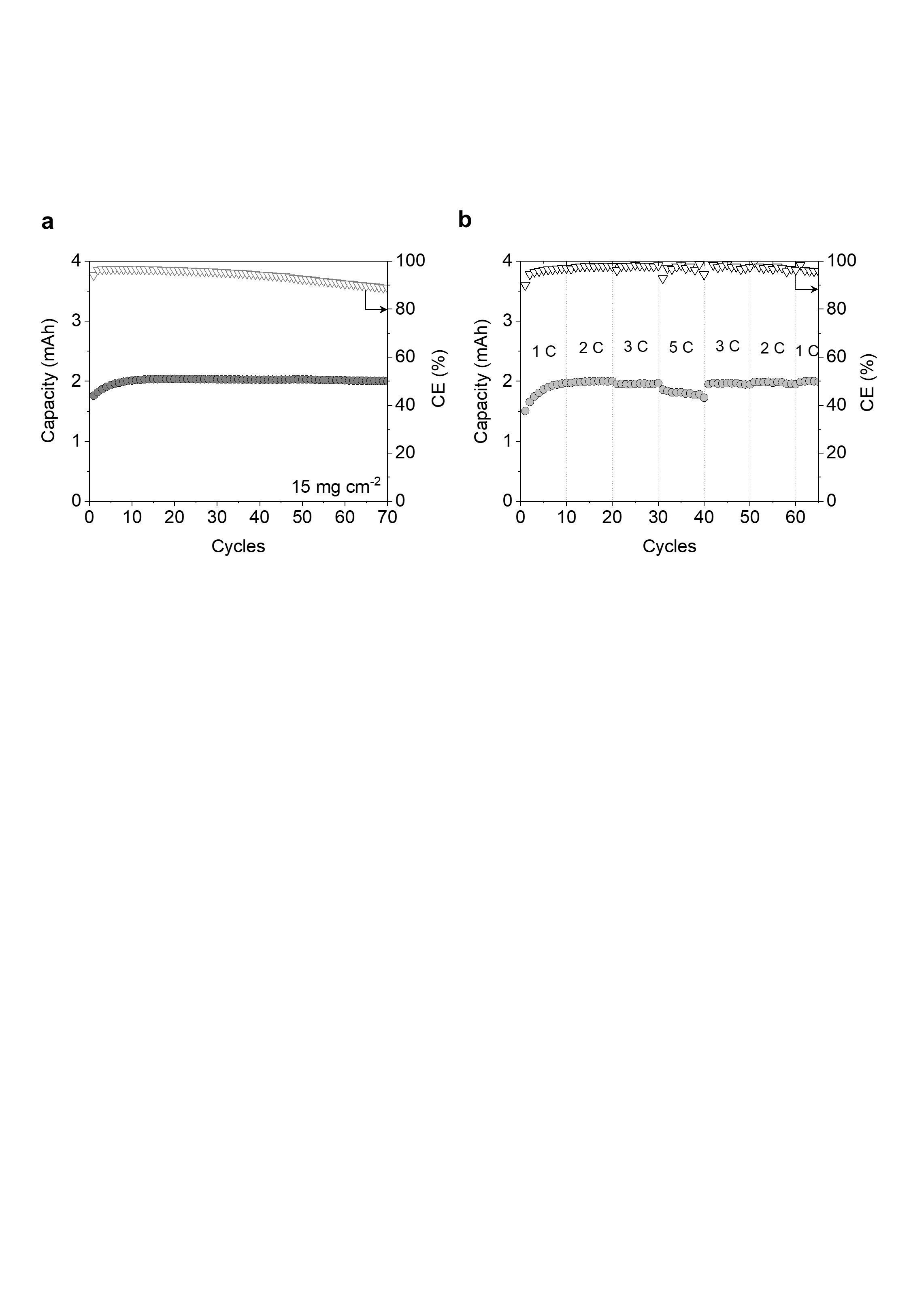


**Figure S23.** (a) Cycling performance at 1 C and (b) rate capability of the DIB cell using DWSE and a high-mass-loading graphite cathode (15 mg cm^-2^).


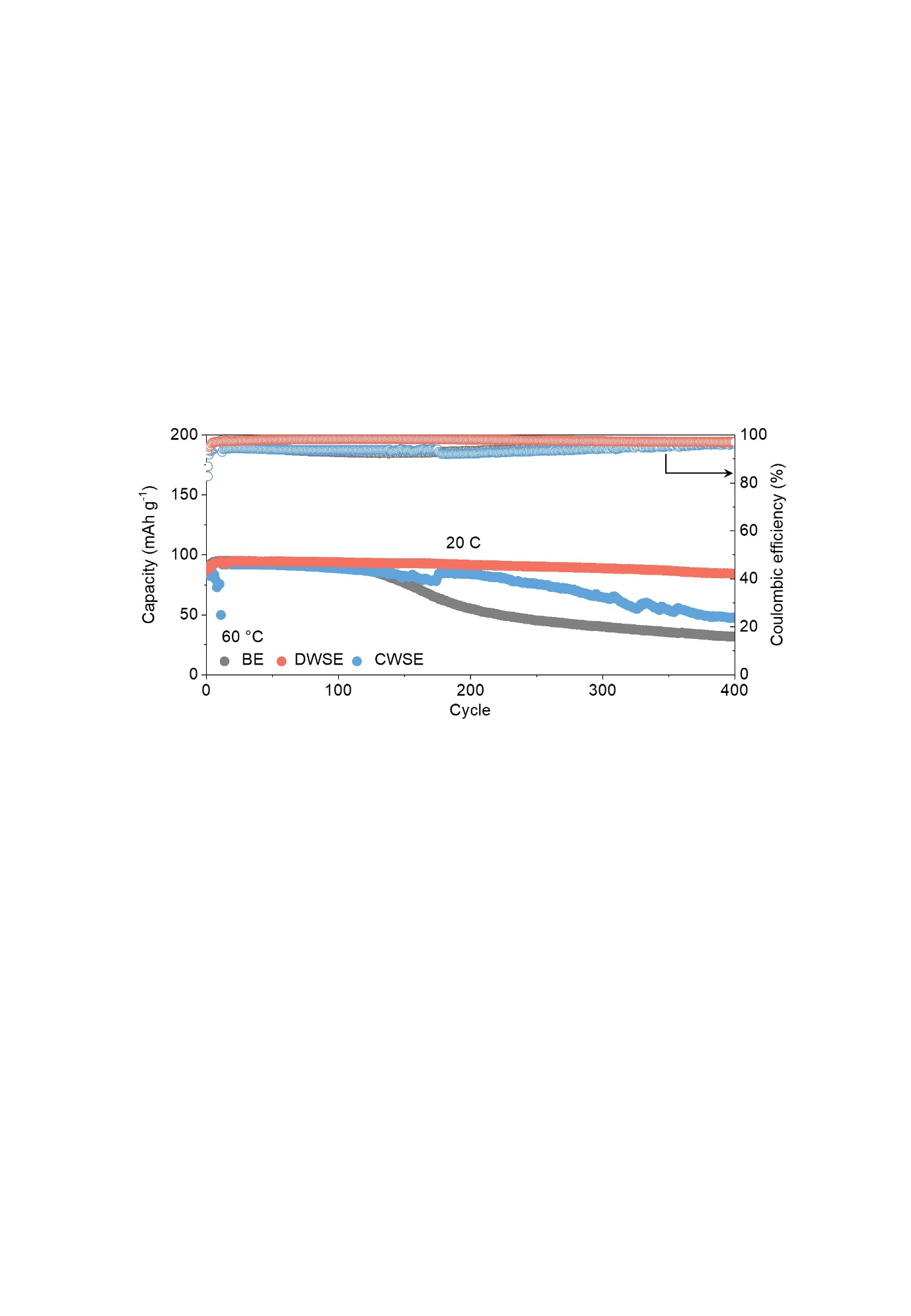


**Figure S24.** High-temperature cycling stability evaluation of DIB cells based on BE, DWSE, and CWSE at 60 ℃ and 20 C.

**
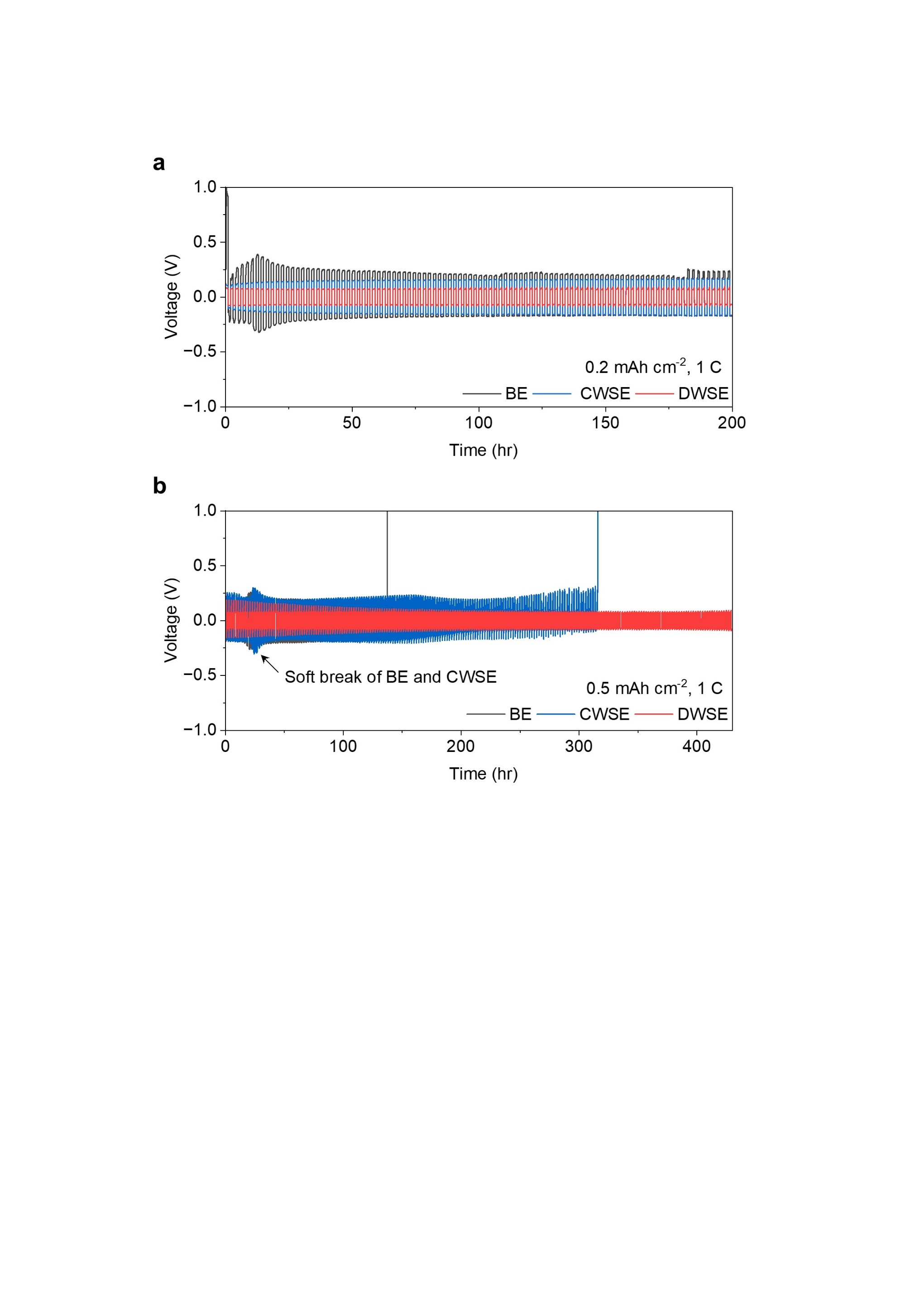
**

**Figure S25.** Cycling performance of Na|Na symmetric cells using three different electrolytes (BE, CWSE, and DWSE) at (a) 0.2 mAh cm^-2^ and (b) 0.5 mAh cm^-2^, both under a current density at 1 C.


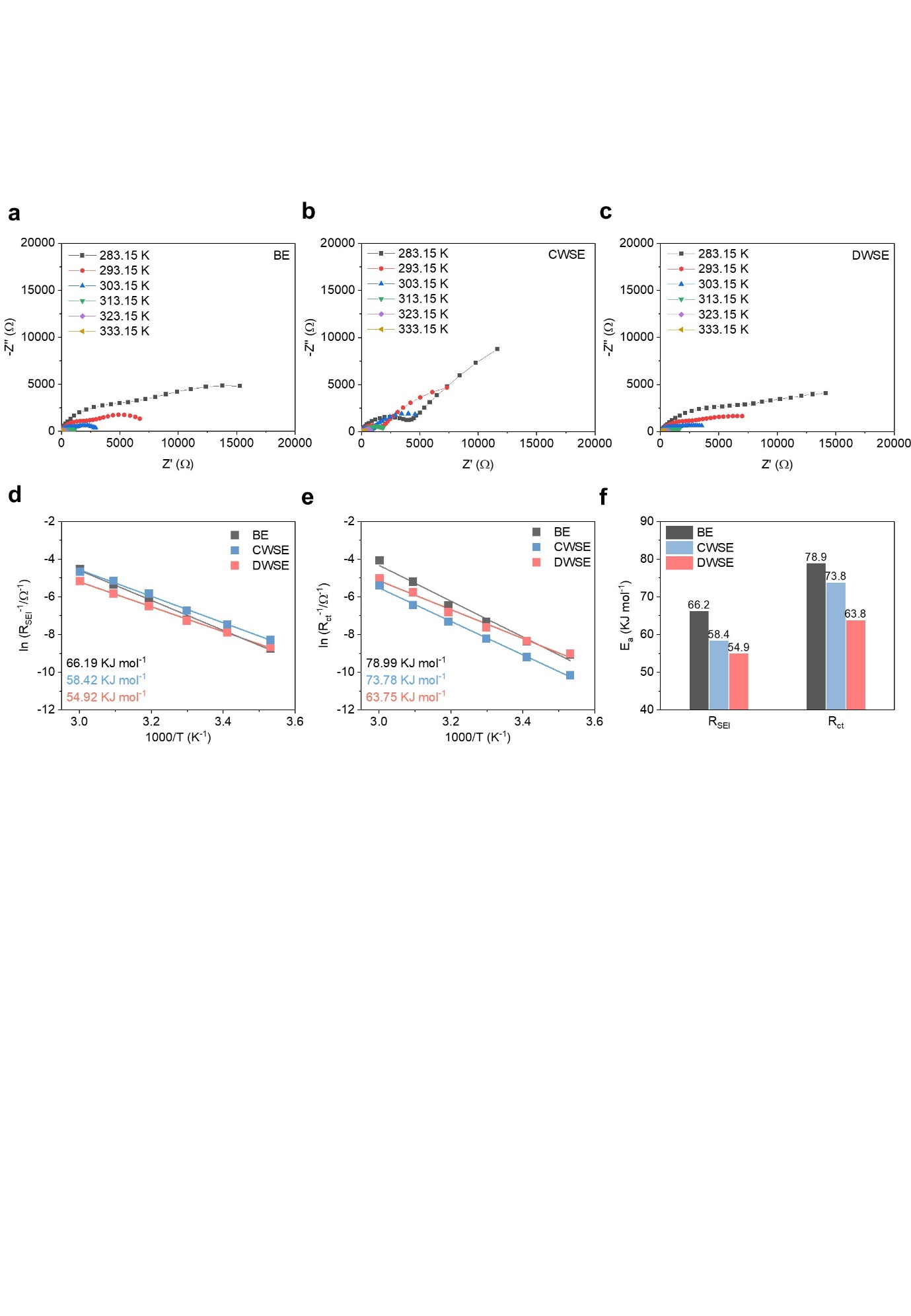


**Figure S26.** Temperature-dependent EIS of Na|Na cells with (a) BE, (b) CWSE, and (c) DWSE. The fitting results of (d) R_SEI_ and (e) R_ct_ based on the Arrhenius equation. (f) Calculated activation energies (E_a_) derived from the Arrhenius equation fitting.


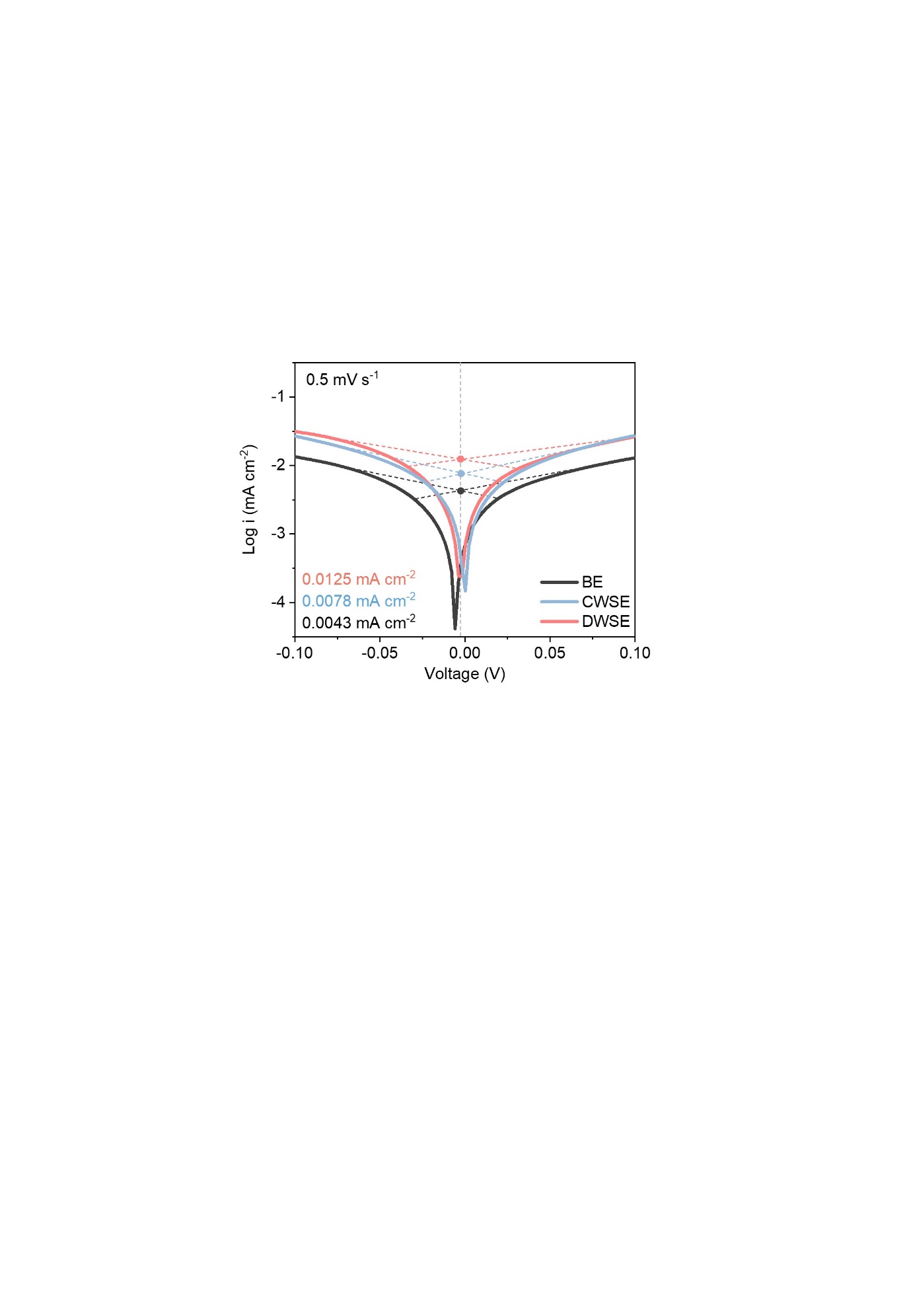


**Figure S27.** The exchange current density of different electrolytes.


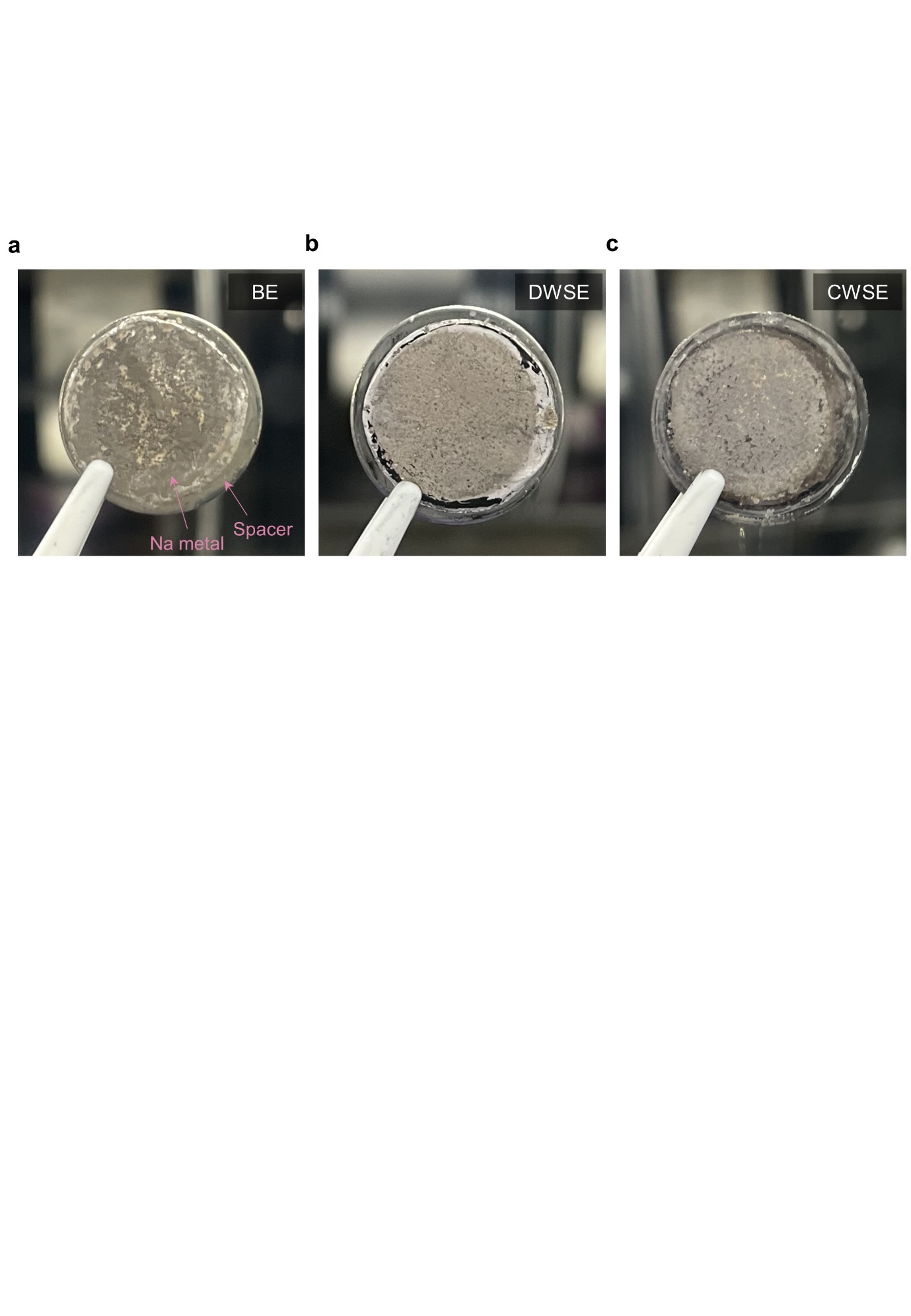


**Figure S28.** Photographs of sodium metal anodes from DIB cells using (a) BE, (b) DWSE, and (c) CWSE after 500 cycles. The images show sodium metal adhered to the spacer.


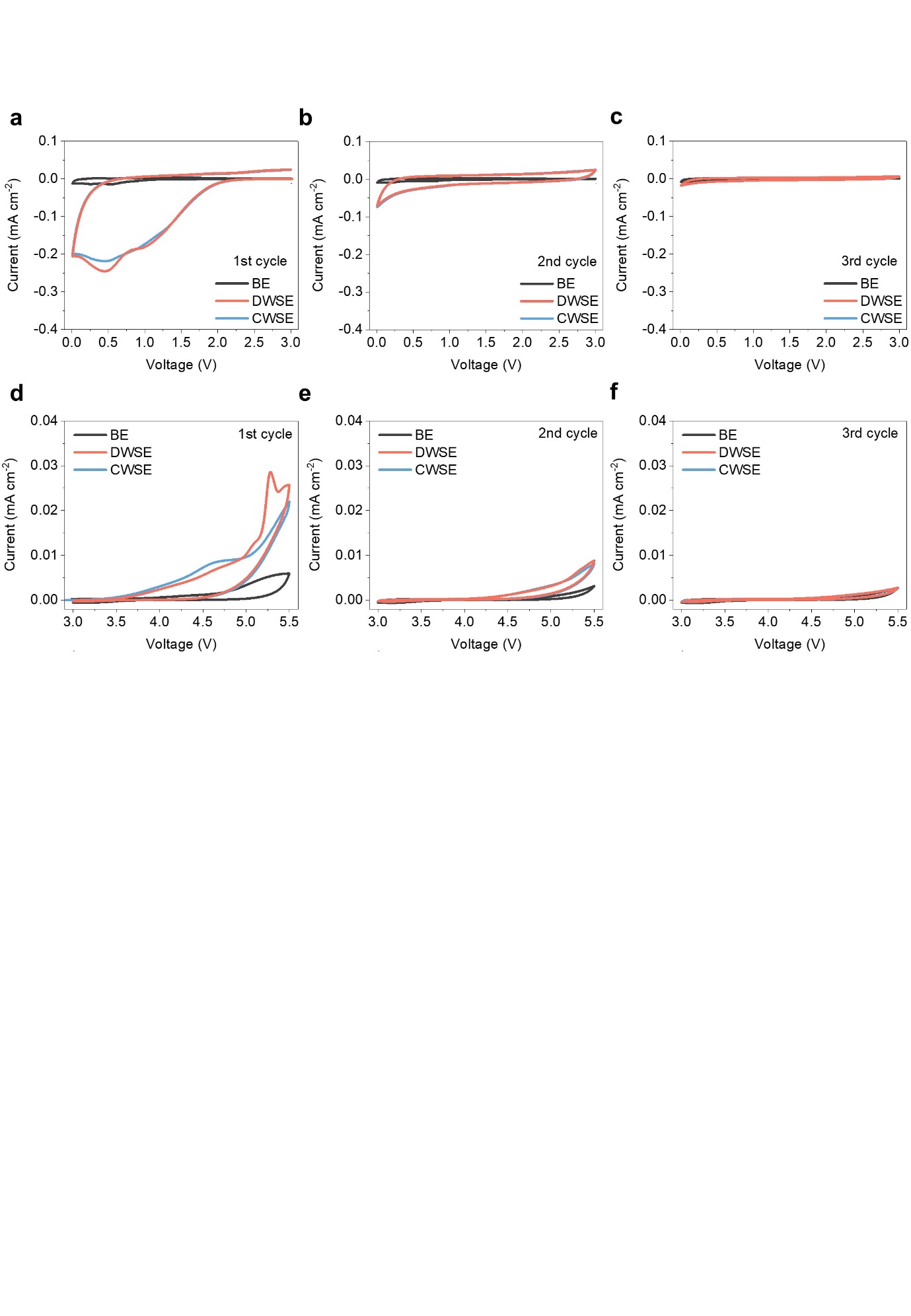


**Figure S29.** Cyclic voltammetry curves for the three electrolytes in the (a-c) 0–3 V region and (d-f) 3–5.5 V region. A significant reduction in peak current is observed over three cycles for DWSE and CWSE, indicating enhanced stability.


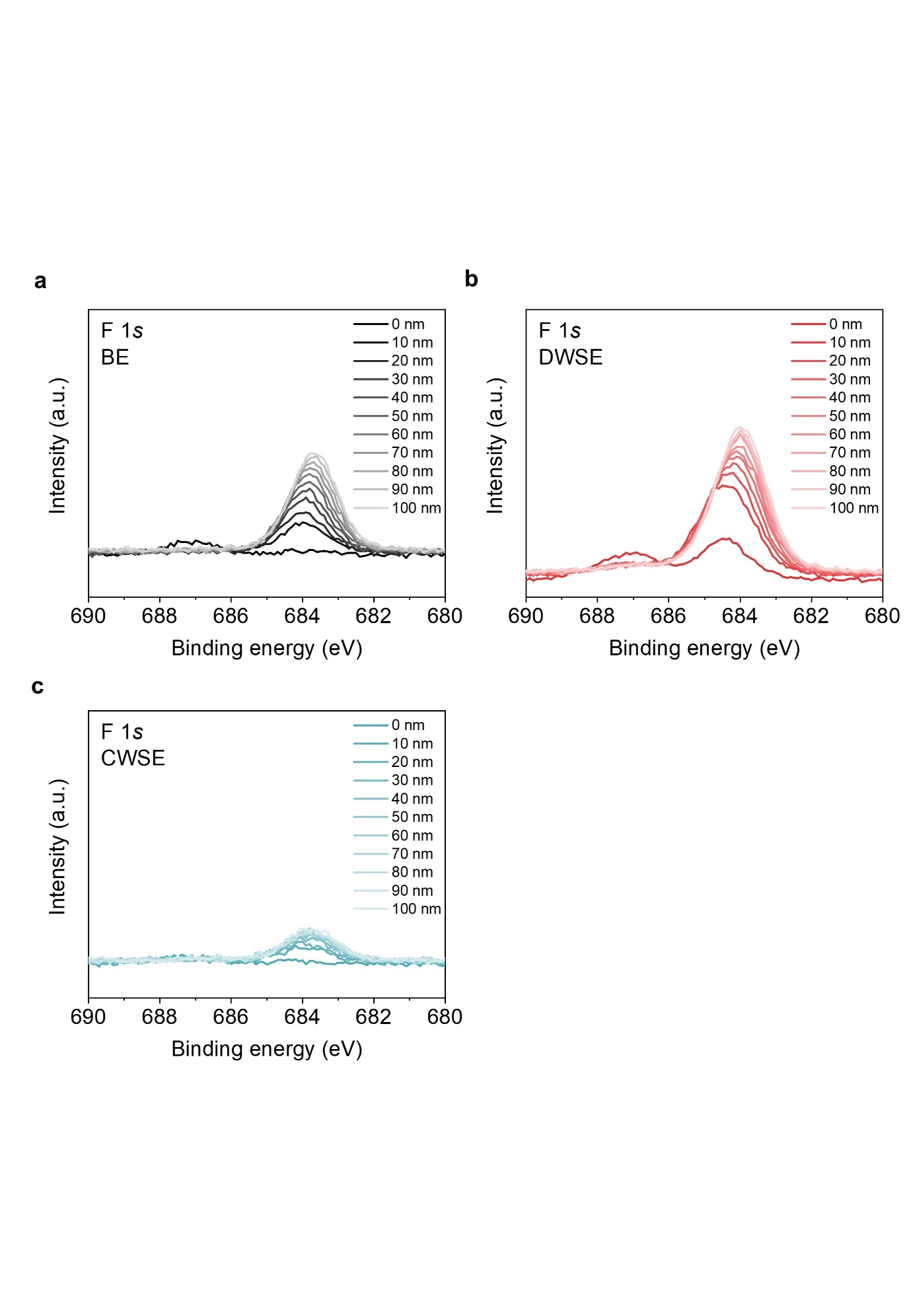


**Figure S30.** XPS depth profiling graphs of the F 1s spectra corresponding to the SEI on the sodium metal anode for (a) BE, (b) DWSE, and (c) CWSE.


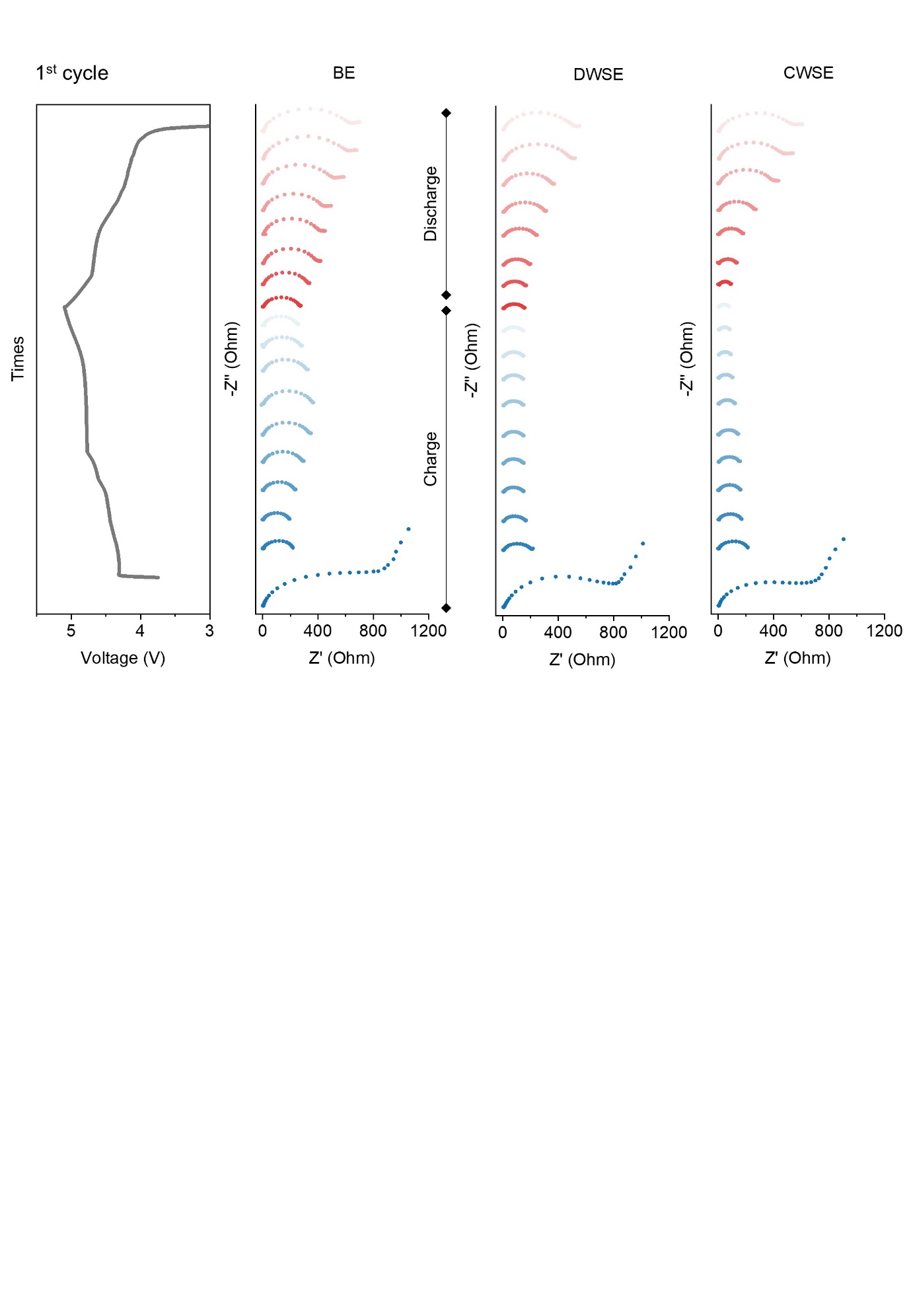


**Figure S31.** In operando EIS spectra measured during the first cycle of DIB cells using the three different electrolytes.


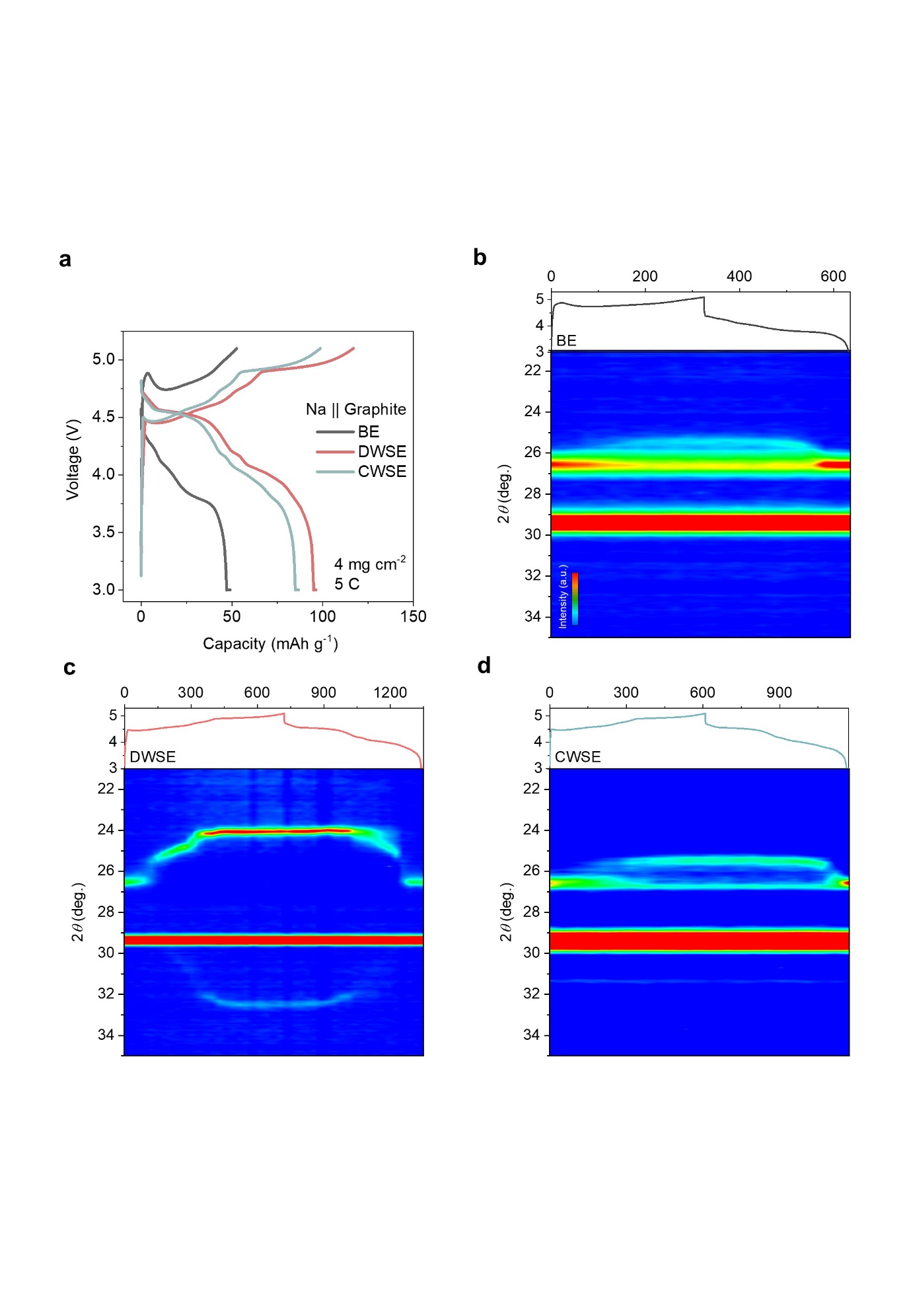


**Figure S32.** In operando synchrotron XRD measurements of the three electrolytes. (a) Voltage profiles of DIB cells assembled with 4 mg cm^-2^ graphite cathodes and operated at 5 C. Time-resolved XRD patterns of graphite cathodes in DIB cells using (b) BE, (c) DWSE, and (d) CWSE. Top panels show galvanostatic voltage profiles, while bottom panels present corresponding XRD patterns during cycling.


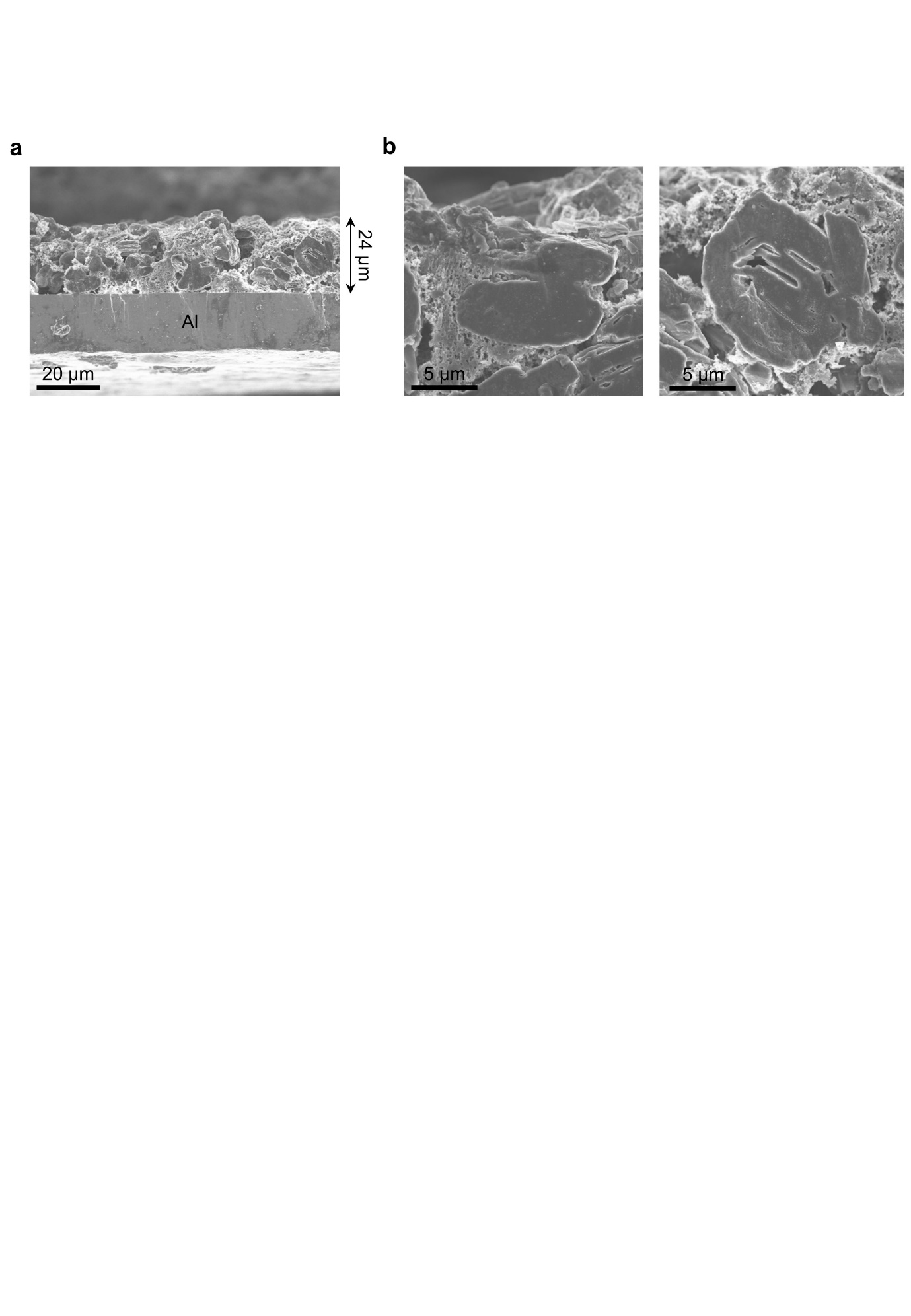


**Figure S33**. SEM image of (a) the pristine graphite cathode and (b) magnified SEM images of pristine graphite particles.


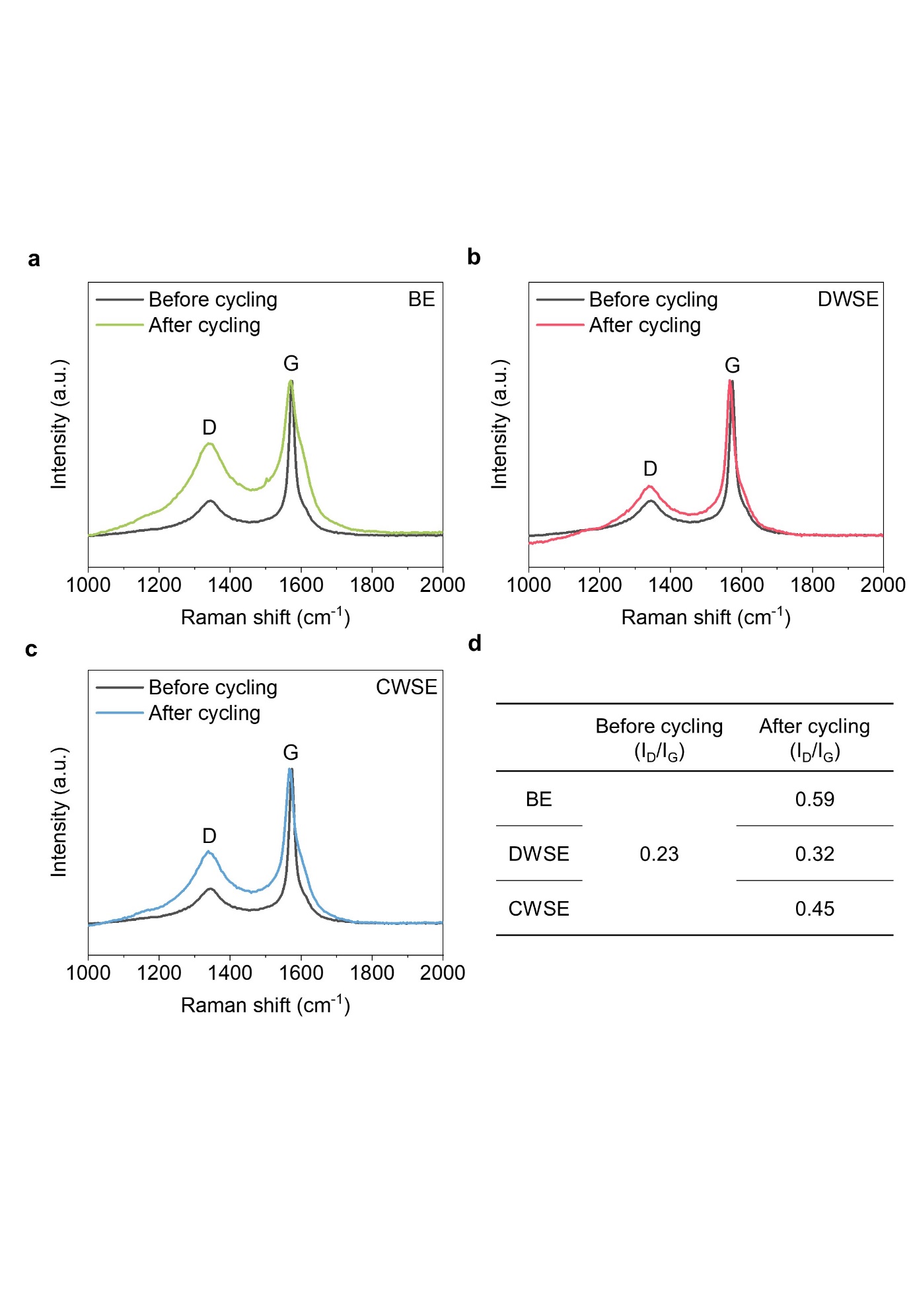


**Figure S34.** Raman spectra of graphite cathodes before and after cycling with (a) BE, (b) DWSE, and (c) CWSE. (d) Comparison of I_D_/I_G_ values obtained from Raman spectra.


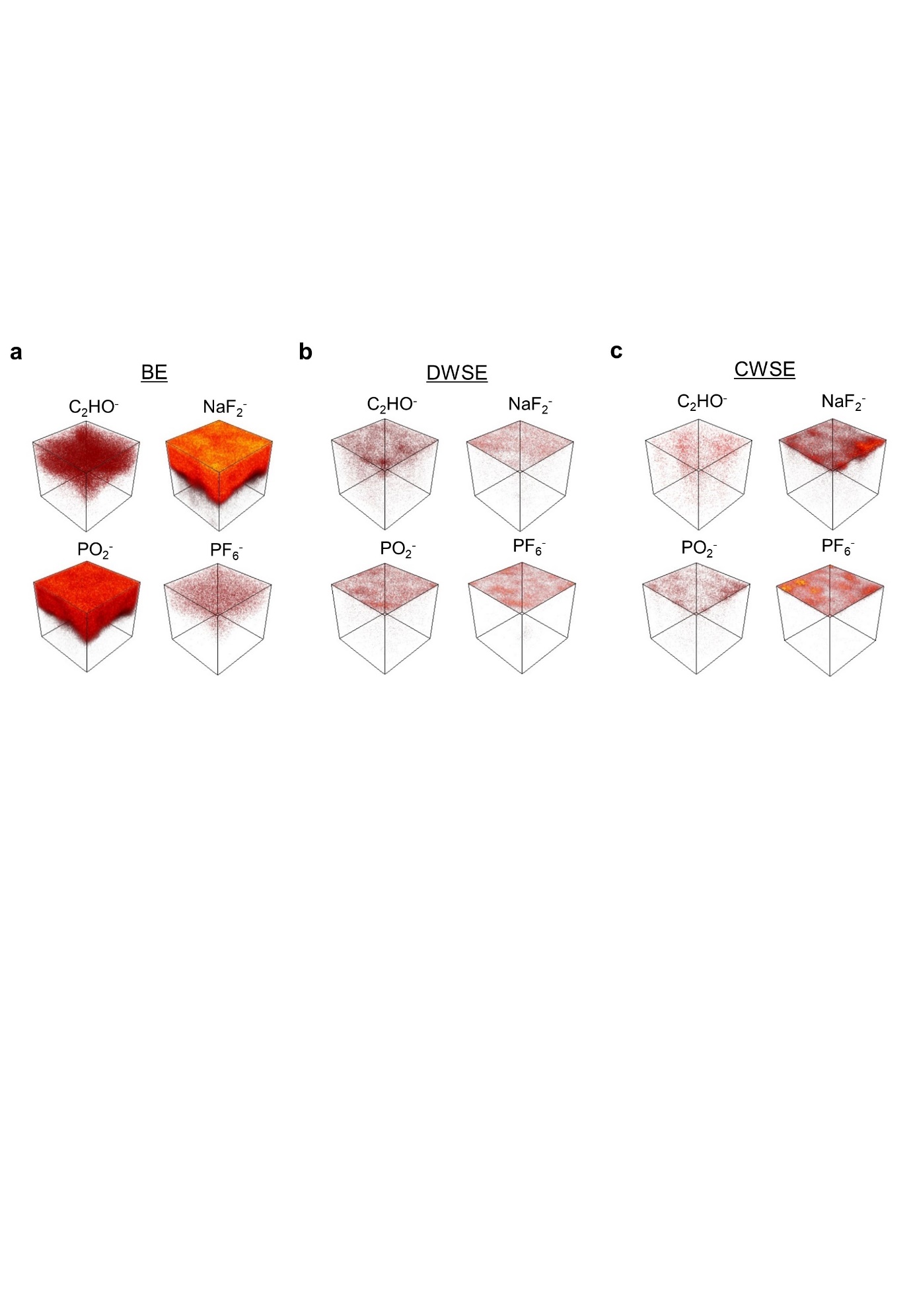


**Figure S35.** TOF-SIMS analysis of graphite cathodes after cycling with (a) BE, (b) DWSE, and (c) CWSE, conducted in negative ion mode to observe electrolyte decomposition products.

**Table S1**. Performance comparison of reported Na-based DIBs.

| Cell configuration  (anode\|\|cathode) | Electrolyte | Discharge capacity (mAh g^‑1^) | Voltage range (V) | Cycle life  (1 C = 100 mA g^-1^) | Coulombic efficiency (%) | Ref. |
| --- | --- | --- | --- | --- | --- | --- |
| Na\|\|Graphite | 1.0 m NaPF_6_ in EMC + 5 wt% FEC + 10 mg/ml CNG | 98.92 at 100 mA g^-1^  97.41 at 500 mA g^-1^  92.68 at 2000 mA g^-1^  82.90 at 5000 mA g^-1^ | 3.0-5.1 | 97.4% after 1500 cycles at 10 C  95.8% after 400 cycles at 20 C (60 ℃) | 92.40 at 1 C  95.36 at 2 C  96.60 at 3 C  97.66 at 5 C  98.75 at 10 C  99.22 at 20 C  99.82 at 30 C | This work |
| Na\|\|Graphite | 1.0 m NaFSI in Cl-EMC  4.0 m NaFSI in Cl-EMC | 104.6 at 100 mA g^-1^  97.6 at 400 mA g^-1^  (4.0 m) | 3.0-5.0 | ~100% after 911 cycles at 1 C (4.0 m) | 79.4 at 1 C (1.0 m)  92.8 at 1 C (4.0 m) | 1 |
| Na\|\|Graphite | NaTFSI:TMP 1:2 | 52.9 at 500 mA g^-1^ | 3.0-4.8 | ~100% after 750 cycles at 5 C | ~99 at 5 C | 2 |
| Na\|\|Graphite | 1.0 M NaPF_6_ in  EC:DMC:EMC (1:1:1,v:v:v)  +10 wt% FEC | ~40 at 500 mA g^-1^ | 2.0-4.8 | 98.7% after 500 cycles at 5 C | ~95 at 2 C | 3 |
| Na\|\|Graphite | 3.0 M NaPF_6_ in EMC:PC (1:1,v:v)+10% FEC | 100 at 100 mA g^-1^  ~94.5 at 1000 mA g^-1^  ~90 at 1600 mA g^-1^ | 3.0-5.0 | 92% after 1000 cycles at 1 C | - | 4 |
| Na\|\|Graphite | 0.5 M NaPF_6_ in PC:EMC:FEC (1:1:1; v:v:v) + 4 wt% PS + 1.5 wt% EPTA + 0.1 wt% AIBN  (GPE) | 84.3 at 500 mA g^-1^ | 3.0-5.0 | 86.7% after 1000 cycles at 1 C | 98.3 at 1 C | 5 |
| Na\|\|PTPAn | 0.5 M NaPF_6_ in DEGDME | 102 at 100 mA g^-1^ | 1.0-3.9 | ~100% after 100 cycles at 100 mA g^-1^ | 99.3 at 10 C | 6 |
| MoS_2_/Carbon fiber@MoS_2_@C  \|\|Graphite | 1.0 M NaPF_6_ in EC/DMC (6:4, v/v) | 101.3 at 500 mA g^-1^  82.5 at 1000 mA g^-1^  63.6 at 2000 mA g^-1^ | 1.0-4.5 | ~86% after 100 cycles at 500 mA g^-1^ | ~95 at 5 C | 7 |
| TiSe_2_\|\|Graphite | 1.0 M NaPF_6_ in EC/EMC/DMC (1:1:1, v/v/v) | 81.8 at 100 mA g^-1^  45.0 at 500 mA g^-1^ | 1.5-3.5 | 83.52% after 200 cycles at 100 mA g^-1^ | ~87 at 1 C | 8 |
| Hard carbon  \|\|Graphite | 1.0 M NaPF_6_ in PC | 62 at 40 mA g^-1^  ~50 at 100 mA g^-1^ | 1.5-4.8 | ~90% after 90 cycles at 100 mA g^-1^ | - | 9 |
| Soft carbon  \|\|Graphite | 1.0 M NaPF_6_ in EC/DMC (6:4, v/v) | 73 at 500 mA g^-1^  56 at 1000 mA g^-1^  40 at 2000 mA g^-1^ | 2.0-4.7 | 97.5% after 600 cycles at 1000 mA g^-1^ | ~90 at 5 C | 10 |
| P-HCN  \|\|Expanded graphite | 1.0 M NaPF_6_ in EC/DEC (4:6, v/v) | 90 at 800 mA g^-1^  80 at 1000 mA g^-1^  45 at 2000 mA g^-1^ | 2.0-4.7 | 90% after 1500 cycles at 500 mA g^-1^ | - | 11 |
| Sn\|\|Expanded graphite | 1.0 M NaPF_6_ in EC/EMC/DMC (1:1:1, v/v/v)  +PVDFHFP-Al_2_O_3_  (ex situ GPE) | 96.8 at 500 mA g^-1^  95.9 at 1000 mA g^-1^  91.6 at 1200 mA g^-1^ | 2.0-4.8 | 97.5% after 600 cycles at 500 mA g^-1^ | ~95 at 5 C | 12 |
| NLHC\|\|FPHC | 1.0 M NaPF_6_ in PC | ~100 at 500 mA g^-1^  ~95 at 1000 mA g^-1^  ~87 at 2000 mA g^-1^  ~80 at 3000 mA g^-1^ | 2.2-4.2 | ~55% after 5000 cycles at 2000 mA g^-1^ | ~100 at 20 C | 13 |
| Na_2_Ti_3_O_7_\|\|Coronene | 1.0 M NaPF_6_ in EC/DEC (1:1, v/v) | ~100 at 500 mA g^-1^  ~60 at 1000 mA g^-1^ | 1.5-3.5 | 80% after 5000 cycles at 500 mA g^-1^ | 98 at 5 C | 14 |

**Supplementary References**

1. Y. Lin, J. Shang, Y. Liu, Z. Wang, Z. Bai, X. Ou and Y. Tang, *Adv. Mater.*, **2024**, 36, 2402702.
2. X. Jiang, X. Liu, Z. Zeng, L. Xiao, X. Ai, H. Yang and Y. Cao, *Adv. Energy Mater.*, **2018**, 8, 1802176.
3. D. Yu, Q. Zhu, L. Cheng, S. Dong, X. Zhang, H. Wang and N. Yang, *ACS Energy Lett.*, **2021**, 6, 949-958.
4. Z. Guo, G. Cheng, Z. Xu, F. Xie, Y.-S. Hu, C. Mattevi, M.-M. Titirici and M. Crespo Ribadeneyra, *ChemSusChem*, **2023**, 16, e202201583.
5. X. Xu, K. Lin, D. Zhou, Q. Liu, X. Qin, S. Wang, S. He, F. Kang, B. Li and G. Wang, *Chem*, **2020**, 6, 902-918.
6. J. Chen, Y. Peng, Y. Yin, Z. Fang, Y. Cao, Y. Wang, X. Dong and Y. Xia, *Angew. Chem. - Int. Ed.*, **2021**, 60, 23858-23862.
7. C. Cui, Z. Wei, J. Xu, Y. Zhang, S. Liu, H. Liu, M. Mao, S. Wang, J. Ma and S. Dou, *Energy Storage Mater.*, **2018**, 15, 22-30.
8. R. Zheng, H. Yu, X. Zhang, Y. Ding, M. Xia, K. Cao, J. Shu, A. Vlad and B.-L. Su, *Angew. Chem. - Int. Ed.*, **2021**, 60, 18430-18437.
9. Z. Hu, Q. Liu, K. Zhang, L. Zhou, L. Li, M. Chen, Z. Tao, Y.-M. Kang, L. Mai, S.-L. Chou, J. Chen and S.-X. Dou, *ACS Appl. Mater. Interfaces.*, **2018**, 10, 35978-35983.
10. L. Fan, Q. Liu, S. Chen, Z. Xu and B. Lu, *Adv. Energy Mater.*, **2017**, 7, 1602778.
11. X. Wang, S. Wang, K. Shen, S. He, X. Hou and F. Chen, *J. Mater. Chem. A*, **2020**, 8, 4007-4016.
12. D. Xie, M. Zhang, Y. Wu, L. Xiang and Y. Tang, *Adv. Funct. Mater.*, **2020**, 30, 1906770.
13. Z. Sun, K. Zhu, P. Liu, X. Chen, H. Li and L. Jiao, *Angew. Chem. - Int. Ed.*, **2022**, 61, e202211866.
14. S. Dong, Z. Li, I. A. Rodríguez-Pérez, H. Jiang, J. Lu, X. Zhang and X. Ji, *Nano Energy*, **2017**, 40, 233-239.
